# Spatial separation between replisome‐ and template‐induced replication stress signaling

**DOI:** 10.15252/embj.201798369

**Published:** 2018-03-26

**Authors:** Néstor García‐Rodríguez, Magdalena Morawska, Ronald P Wong, Yasukazu Daigaku, Helle D Ulrich

**Affiliations:** ^1^ Institute of Molecular Biology (IMB) Mainz Germany; ^2^ Cancer Research UK London Research Institute Clare Hall Laboratories Blanche Lane South Mimms UK; ^3^ Present address: Springer Nature London UK; ^4^ Present address: Frontier Research Institute for Interdisciplinary Sciences Tohoku University Aoba‐ku Sendai Japan

**Keywords:** DNA damage bypass, DNA damage checkpoint, Exo1, postreplication repair, replication stress, DNA Replication, Repair & Recombination

## Abstract

Polymerase‐blocking DNA lesions are thought to elicit a checkpoint response via accumulation of single‐stranded DNA at stalled replication forks. However, as an alternative to persistent fork stalling, re‐priming downstream of lesions can give rise to daughter‐strand gaps behind replication forks. We show here that the processing of such structures by an exonuclease, Exo1, is required for timely checkpoint activation, which in turn prevents further gap erosion in S phase. This Rad9‐dependent mechanism of damage signaling is distinct from the Mrc1‐dependent, fork‐associated response to replication stress induced by conditions such as nucleotide depletion or replisome‐inherent problems, but reminiscent of replication‐independent checkpoint activation by single‐stranded DNA. Our results indicate that while replisome stalling triggers a checkpoint response directly at the stalled replication fork, the response to replication stress elicited by polymerase‐blocking lesions mainly emanates from Exo1‐processed, postreplicative daughter‐strand gaps, thus offering a mechanistic explanation for the dichotomy between replisome‐ versus template‐induced checkpoint signaling.

## Introduction

Genome maintenance relies on checkpoint pathways that perceive DNA damage or replication problems and initiate an appropriate response. In eukaryotic cells, they are mediated by kinase cascades activated by distinct types of abnormal DNA structures (Nyberg *et al*, 2002). In vertebrates, damage signaling by the ATM kinase is initiated by DNA double‐strand breaks (DSBs), whereas the related ATR kinase reacts to a variety of lesions and is activated mainly by single‐stranded DNA (ssDNA). During S phase, cells are particularly vulnerable to conditions that challenge the progression of the replisome. In this situation, ssDNA is thought to accumulate at stalled replication forks by an uncoupling between helicase and polymerase movement or between leading and lagging strand synthesis. In budding yeast, the checkpoint response elicited by these structures is initiated by Mec1, the homologue of vertebrate ATR, which is responsible for activating an effector kinase, Rad53. Via phosphorylation of a large set of substrates, Rad53 mediates most aspects of the checkpoint response, including a stabilization of stalled forks, suppression of late origin firing, control of nucleotide levels, regulation of damage‐induced transcription, and arrest of the cell cycle (Pardo *et al*, 2017). Intriguingly, checkpoint signaling in response to replication stress can be divided into two branches that both initiate from Mec1 and converge on Rad53, but differ in the mediator protein responsible for signal transmission: the DNA replication checkpoint and the DNA damage checkpoint (Pardo *et al*, 2017). Upon inhibition of ribonucleotide reductase by hydroxyurea (HU), Mec1 phosphorylates the replisome component, Mrc1, a homologue of claspin. In response to DNA damage, Mec1 cooperates with the 53BP1 homologue Rad9. This dichotomy has led to the speculation that a replication fork stalled by nucleotide depletion adopts a structure distinct from one that is stalled by a lesion in the template (Alcasabas *et al*, 2001; Nielsen *et al*, 2013). However, the basis for such difference remains unclear.

Outside of S phase, ssDNA as a source of checkpoint activation can arise from nucleotide excision repair (NER) or from the resection of 5′‐termini at DSBs or uncapped telomeres. In both situations, a 5′–3′ exonuclease, Exo1, contributes to Rad53 activation by widening NER gaps or processing DNA termini (Nakada *et al*, 2004; Dewar & Lydall, 2010; Giannattasio *et al*, 2010). At the same time, Exo1 is a downstream target of Rad53, which inhibits the nuclease by phosphorylation (Smolka *et al*, 2007; Morin *et al*, 2008). This results in a negative feedback that prevents excessive Exo1 activity. At stalled replication forks, Exo1 degrades abnormal structures and prevents fork reversal, but it does not contribute to damage signaling or replication restart and may even promote fork breakdown (Cotta‐Ramusino *et al*, 2005; Segurado & Diffley, 2008). At collapsed replication forks, Exo1 activity is deemed to be mostly detrimental and is subject to checkpoint‐mediated inhibition (Tsang *et al*, 2014).

As an alternative to persistent fork stalling, re‐priming of DNA synthesis downstream of a lesion can give rise to daughter‐strand gaps behind the replication fork. This has been studied most extensively in bacterial systems (Heller & Marians, 2006), but there is good evidence for a “skipping” of DNA damage‐induced lesions in eukaryotic cells as well (Lopes *et al*, 2006; Elvers *et al*, 2011). Ultimately, however, cell proliferation requires complete genome replication, necessitating the activity of DNA damage bypass pathways to copy the damaged DNA (Friedberg, 2005; Ulrich, 2009). Importantly, these pathways, initiated by the ubiquitylation of the replication factor PCNA (Hoege *et al*, 2002) and involving either translesion synthesis by specialized, damage‐tolerant polymerases or a recombination‐like process named template switching, are not necessarily coupled to replication fork progression. They can be delayed without major effects on genome stability until bulk genome replication is completed (Ulrich, 2009; Daigaku *et al*, 2010; Karras & Jentsch, 2010), although an impact on the transmission of epigenetic information has been reported (Sarkies *et al*, 2010). Under these conditions, daughter‐strand gaps accumulate and give rise to a damage response, accompanied by a cell cycle arrest in G2/M phase (Lopes *et al*, 2006; Callegari *et al*, 2010; Daigaku *et al*, 2010). When damage bypass is re‐activated at that point, the pathway mediates the filling of these gaps in a postreplicative manner (Daigaku *et al*, 2010; Karras & Jentsch, 2010).

The significance of re‐priming and daughter‐strand gap formation for checkpoint signaling in *WT* cells is not well understood. A postreplication checkpoint that senses unreplicated DNA has been postulated (Callegari & Kelly, 2006), and Balint *et al* (2015) have described the assembly of a Mec1‐activating complex distal to replication forks in response to DNA damage induced by the alkylating agent methyl methanesulfonate (MMS). However, the notion of postreplicative checkpoint activation contradicts the established concept of fork uncoupling, which invokes the stalled replication fork as the source of ssDNA that activates checkpoint signaling (Walter & Newport, 2000; Byun *et al*, 2005). In order to resolve this conflict, we made use of a genetic tool to delay damage bypass, thus causing a damage‐dependent hyper‐accumulation of daughter‐strand gaps (Daigaku *et al*, 2010). In this setting, we identified an Exo1‐dependent mechanism of Rad53 activation that in turn prevents erosion of gaps and an irreversible loss of viability largely attributable to the unrestrained activities of Exo1 and Pif1. Although reminiscent of the replication‐independent action of Exo1 at DNA termini and NER gaps, this process required entry into S phase. Importantly, the same Exo1‐dependent mechanism of Rad53 activation was observed in damage bypass‐competent cells specifically during replication of damaged DNA, but not in response to nucleotide depletion or replisome problems. These findings explain the dichotomy between Mrc1‐ and Rad9‐dependent Rad53 activation and suggest two distinct, spatially segregated mechanisms of how replication stress causes checkpoint activation: a fork‐associated, Mrc1‐dependent, Exo1‐independent reaction in response to replisome‐inherent problems and a gap‐associated, Rad9‐ and Exo1‐dependent process that predominates under conditions of template‐induced polymerase stalling. We conclude that even in bypass‐competent cells, regions of ssDNA left behind in the wake of replication forks and expanded by the action of processing factors such as Exo1, rather than stalled replication forks per se, constitute the predominant signal that leads to checkpoint activation in response to polymerase‐stalling DNA lesions during S phase.

## Results

### Rad9‐mediated checkpoint signaling is essential for damage resistance in the absence of damage bypass

**Figure 1:**
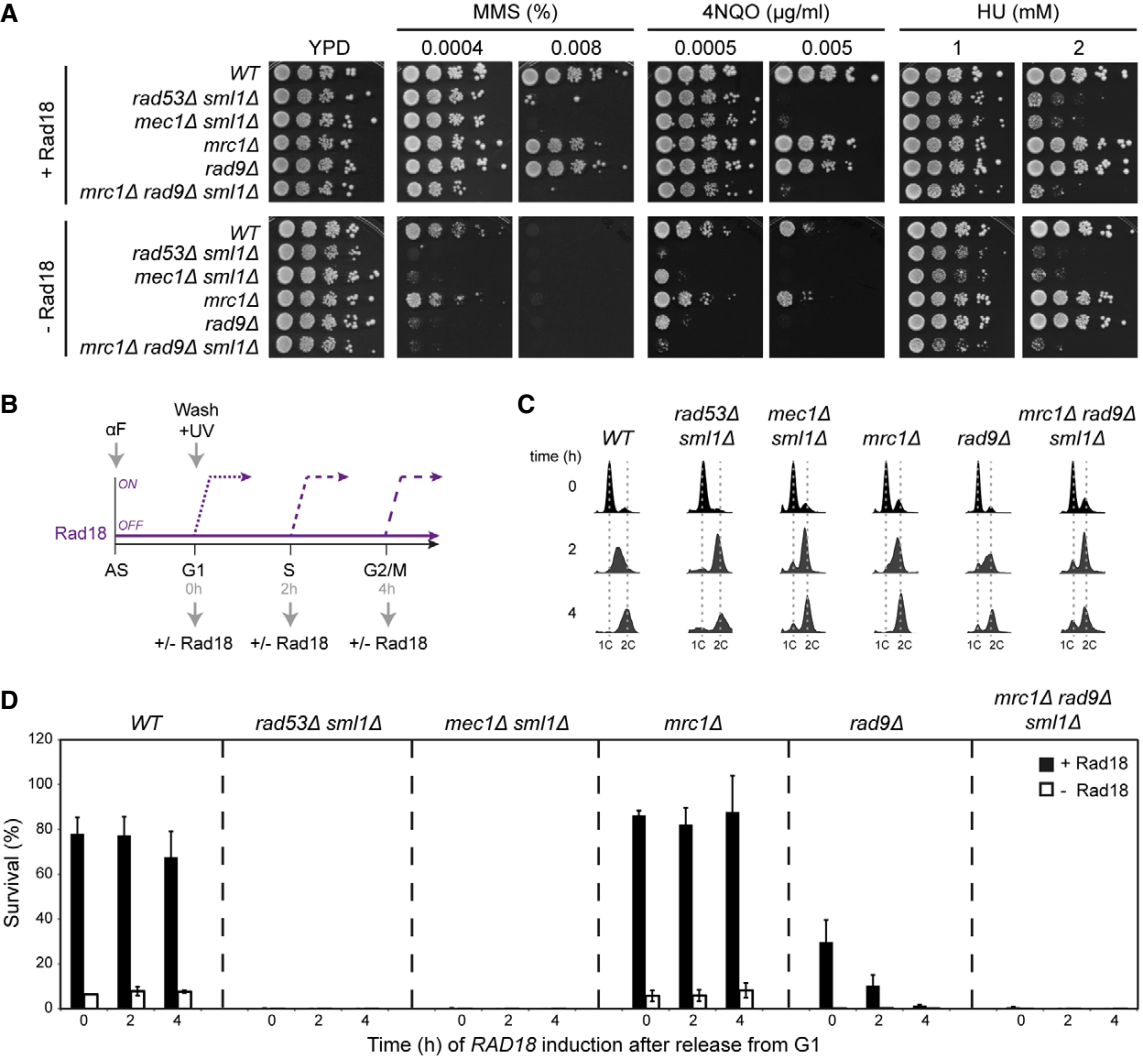
Contribution of checkpoint factors to DNA damage bypass DNA damage sensitivities of *Tet‐RAD18* strains carrying the indicated gene deletions, determined by growth assays in the presence (top) or absence (bottom) of Rad18.Experimental scheme for measuring recovery of viability after UV irradiation (20 J/m^2^) upon *Tet‐RAD18* induction at the indicated times after release into S phase (AS: asynchronous; αF: alpha‐factor). For details, see Materials and Methods.Cell cycle profiles of the indicated strains at the time of plating.Survival of the indicated strains, relative to unirradiated controls. Error bars indicate SD derived from three independent experiments.

**Figure EV1:**
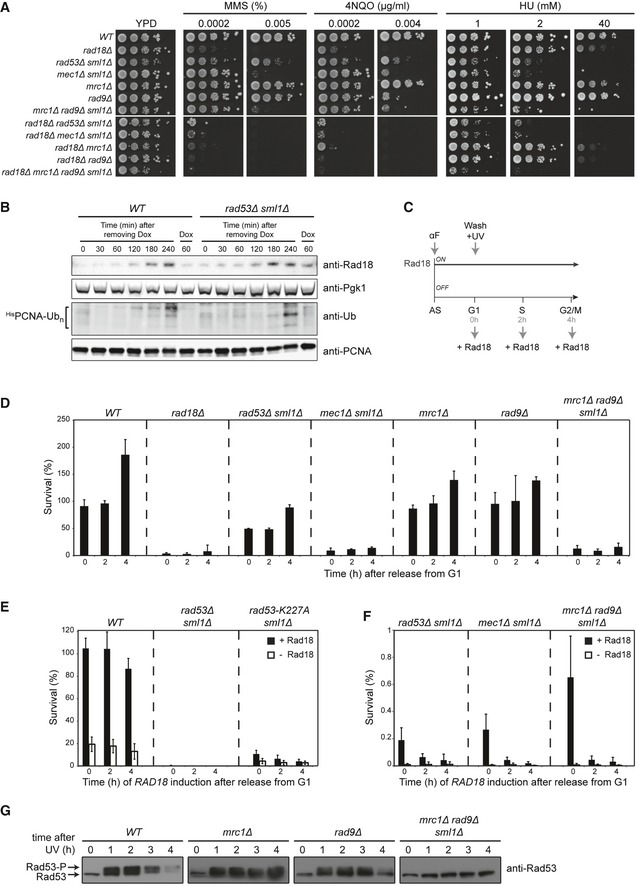
Contribution of checkpoint factors to DNA damage bypass DNA damage sensitivities of yeast strains carrying the indicated gene deletions, determined by growth assays.Re‐expression of Rad18 (top) and ^His^PCNA ubiquitylation (bottom) after removing doxycycline in UV‐irradiated cells of the indicated strains. Ubiquitylation of His_6_‐tagged PCNA was detected as described previously (Daigaku *et al*, 2010).Experimental scheme for measuring viability after UV irradiation (20 J/m^2^) at the indicated times after release into S phase, performed as described in Fig 1B, but under conditions of continuous *Tet‐RAD18* expression (AS: asynchronous; αF: alpha‐factor).Survival of the indicated strains, relative to unirradiated controls. Survival above 100% reflects cell division within 4 h.Recovery of viability is abolished in a catalytically inactive *rad53* mutant.Magnification of selected panels from Fig 1D.Rad53 phosphorylation in the specified strains upon release into S phase after UV irradiation in the absence of Rad18, monitored by Western blotting.Data information: (D–F) Error bars indicate SD derived from at least three independent experiments.Source data are available online for this figure.

In order to systematically explore the relationship between checkpoint activation and damage bypass, we depleted Rad18, the ubiquitin ligase responsible for initiating the pathway (Hoege *et al*, 2002), thus enforcing hyper‐accumulation of daughter‐strand gaps during replication over lesions (Daigaku *et al*, 2010; Karras & Jentsch, 2010). In order to avoid the accumulation of suppressors, we used a regulable allele, *Tet‐RAD18*, which conveys a *rad18Δ*‐like phenotype only in the presence of doxycycline (Daigaku *et al*, 2010). We monitored the effects of Rad18 loss on defined checkpoint mutants with respect to three different types of genotoxic stress: the methylating agent MMS, which elicits a damage response primarily during replication, 4‐nitroquinoline oxide (4NQO), which forms bulky adducts that are perceived in a replication‐independent manner, and HU, which causes replication fork stalling by means of nucleotide depletion without inducing lesions in the replication template. Depletion of Rad18 strongly sensitized the checkpoint mutants *mec1Δ*,* rad53Δ,* and *mrc1Δ rad9Δ* toward MMS and 4NQO, confirming the importance of checkpoint signaling in the absence of damage bypass (Fig 1A). Synergism was also observable with *rad9Δ* alone, but not with *mrc1Δ*, suggesting that the DNA damage checkpoint—as opposed to the replication checkpoint—was responsible for the effect. In support of this model, depletion of Rad18 only mildly enhanced the sensitivity of any of the strains toward moderate concentrations of HU, indicating that replication fork stalling per se is not particularly detrimental in the absence of Rad18. A *RAD18* deletion yielded comparable results (Fig EV1A). These findings imply a synergistic impact of damage bypass and specifically Rad9‐dependent checkpoint signaling on the processing of DNA lesions.

### Rad9‐mediated checkpoint signaling maintains damage bypass competence during S phase

Rad18 is a rate‐limiting factor for PCNA ubiquitylation. Hence, the *Tet‐RAD18* allele allows us to modulate the activation of the damage bypass pathway at will in the course of a cell cycle. In this manner, we had previously shown that synchronized cells, treated in the G1 phase with low doses of ultraviolet (UV) radiation in the absence of Rad18, replicate the bulk of their genomes, but stall in G2/M phase with an activated checkpoint due to the hyper‐accumulation of daughter‐strand gaps (Daigaku *et al*, 2010). *RAD18* re‐expression at any time during or after genome replication allows them to recover, indicating that postreplicative gap filling can substitute for replication‐associated damage bypass. We now used this approach to examine the mechanism of checkpoint activation under conditions of daughter‐strand gap hyper‐accumulation (Fig 1B): Alpha‐factor (αF)‐arrested G1 cells were UV‐irradiated in the absence of Rad18 and subsequently released into S phase. *RAD18* expression was then induced either immediately upon release, in mid‐S phase, or after cells had reached G2/M phase (Fig 1C), and survival was determined by plating of aliquots. As previously reported, checkpoint‐proficient (*WT*) cells recovered viability independently of the timing of *RAD18* induction (Daigaku *et al*, 2010). In contrast, *mec1Δ* and *rad53Δ* mutants were completely unable to recover (Fig 1D). In the case of *mec1Δ*, the defect might be ascribed to a direct participation of the kinase in translesion synthesis by phosphorylation of Rev1 (Pages *et al*, 2009), but for *rad53Δ* this does not apply. Here, the defect was neither due to a failure to re‐express *RAD18* (Fig EV1B) nor caused by the UV sensitivity conferred by the *rad53Δ* mutation itself, as viability remained consistently higher when the assay was performed in the continuous presence of Rad18 (Fig EV1C and D). Hence, in *rad53Δ* cells even a temporary absence of Rad18 appears to cause a complete and irreversible loss of the capacity to productively use damage bypass. The ability to recover by *RAD18* induction depended on the kinase activity of Rad53, as a catalytically deficient mutant, *rad53‐K227A*, did not regain viability (Fig EV1E). As Rad53 can be activated either via the Rad9‐dependent damage checkpoint or the Mrc1‐dependent replication checkpoint (Pardo *et al*, 2017), we examined recovery of viability in *rad9Δ* and *mrc1Δ* mutants. As shown in Fig 1D, the *mrc1Δ* mutant fully recovered upon *RAD18* re‐expression, whereas deletion of *RAD9* caused a significant loss of viability that grew successively more severe with a prolonged delay of *RAD18* induction. In contrast, deletion of *RAD9* or *MRC1* in the presence of *RAD18* had little effect on viability (Fig EV1D). This observation suggests that the Rad9‐mediated damage checkpoint, rather than the Mrc1‐dependent replication checkpoint, is essential to maintain damage bypass competence as cells progress through S phase. An *mrc1Δ rad9Δ* double mutant phenocopied *rad53Δ* mutants (Figs 1D and EV1F), indicating that Mrc1‐mediated checkpoint signaling may partially compensate for the loss of Rad9 during early S phase. Consistent with this model, Rad53 phosphorylation was severely reduced upon *RAD9* deletion, but completely abolished in the *mrc1Δ rad9Δ* double mutant (Fig EV1G). Hence, our findings suggest that Rad9‐mediated activation of the Rad53 kinase becomes essential when damage bypass is delayed.

### Delay of damage bypass in *rad53Δ* mutants causes elevated homologous recombination and catastrophic chromosome fragmentation

**Figure 2:**
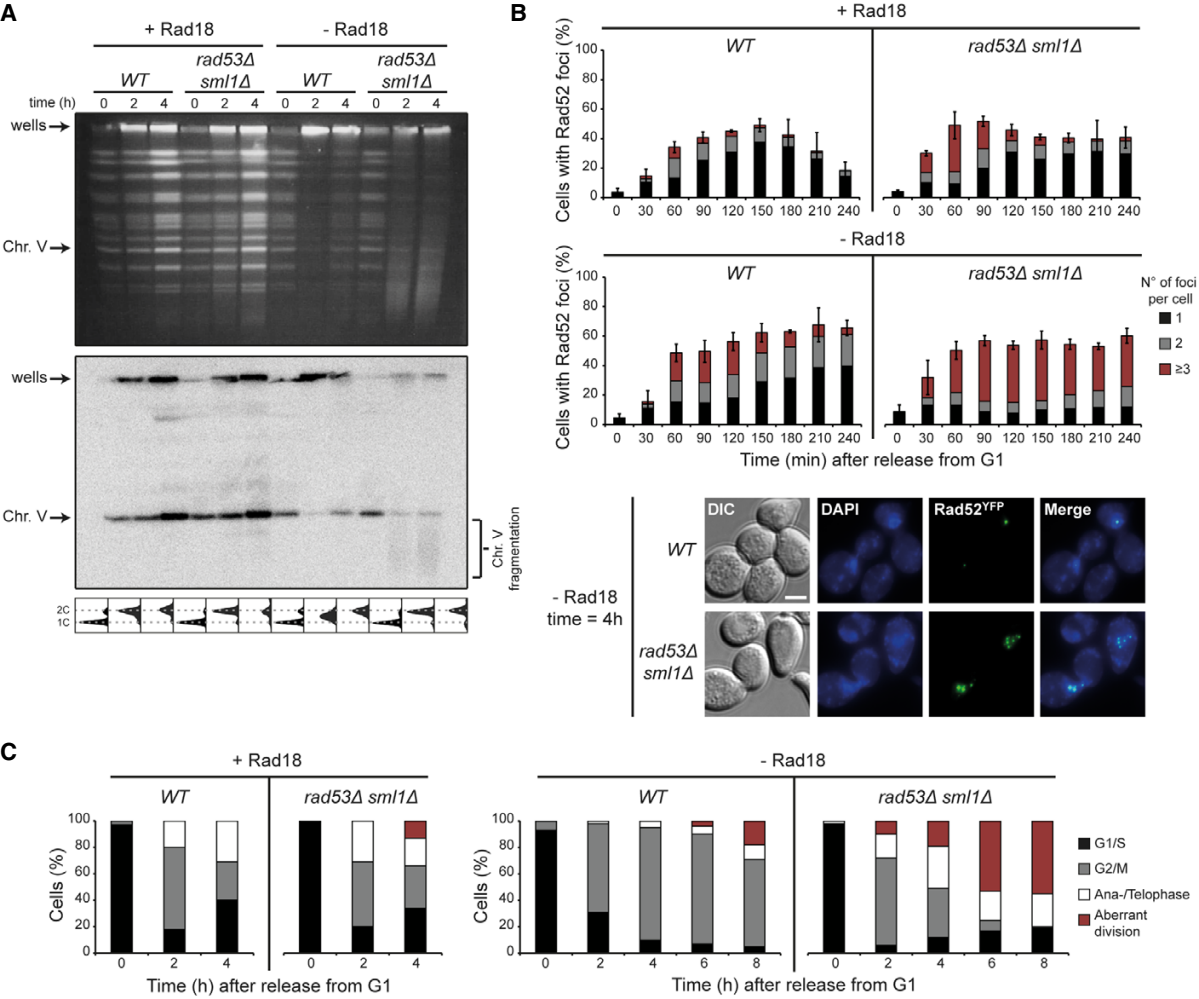
Delay of damage bypass in *rad53Δ* mutants causes chromosome fragmentation, excessive recombination, and aberrant division *WT* and *rad53Δ* cells were grown in the presence or absence of Rad18, synchronized in G1, UV‐irradiated, and released into S phase. 
Yeast chromosomes, analyzed by pulsed‐field gel electrophoresis and ethidium bromide staining (top) or Southern blotting for chromosome V (middle). Replication intermediates accumulate in the wells. Cell cycle profiles are shown below the respective strains.Quantification of Rad52^YFP^ recombination foci and representative images. Error bars indicate SD derived from three independent experiments. Scale bar = 5 μm.Analysis of mitotic aberrations. Cells were classified into cell cycle stages according to spindle morphology (see Fig EV2C and D for examples).

**Figure EV2:**
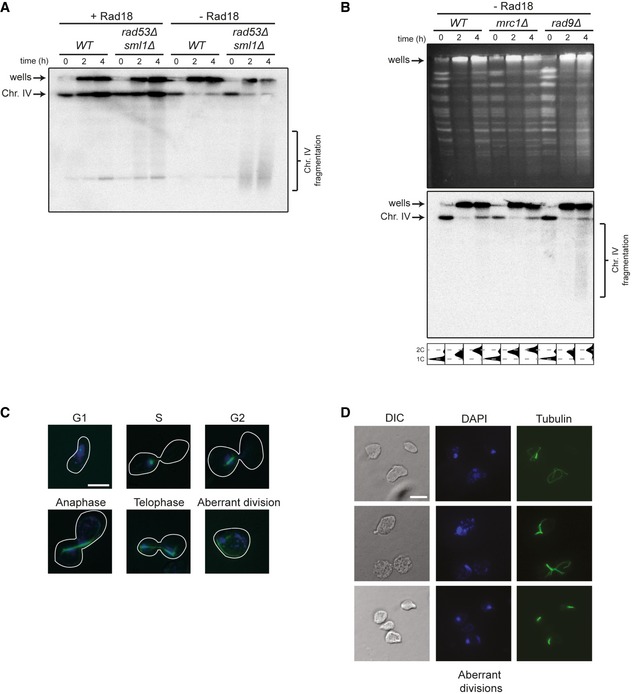
Consequences of delaying damage bypass in checkpoint mutants Pulsed‐field gel electrophoresis and Southern blotting analysis of chromosome IV in *WT* and *rad53Δ* released into S phase after UV irradiation in the presence or absence of Rad18. Replication intermediates accumulate in the wells.Pulsed‐field gel electrophoresis analyzed by ethidium bromide staining (top) and Southern blotting (middle) for chromosome IV in *WT*,* mrc1Δ,* and *rad9Δ* in the absence of Rad18, treated as above. Cell cycle profiles are shown at the bottom.Classification of cells into cell cycle stages according to spindle morphology (blue: DAPI: green: tubulin). Scale bar = 5 μm.Examples of cells undergoing aberrant divisions.

We next sought to elucidate how checkpoint mutants lost viability upon inhibition of damage bypass. Using pulsed‐field gel electrophoresis (PFGE), we found that UV‐irradiated *rad53Δ* cells grown in the absence of Rad18 failed to restore the pattern of intact chromosomes indicative of successful completion of genome replication (Fig 2A). Instead, we observed substantial chromosome fragmentation in the course of S phase (Figs 2A and EV2A). This was also observed in *rad9Δ*, but not in *mrc1Δ* cells (Fig EV2B). Consistent with the fragmentation pattern, *rad53Δ* cells in the absence of Rad18 accumulated strongly elevated numbers of recombination foci and exhibited aberrant chromosome segregation patterns (Figs 2B and C, and EV2C and D). Importantly, both chromosome breaks and hyper‐accumulation of Rad52^YFP^ foci were only observed in the absence of Rad18. From these observations, we conclude that when damage bypass fails, Rad9‐mediated checkpoint signaling is essential to prevent massive chromosome fragmentation during S phase and—likely as a consequence of this—an elevated frequency of aberrant or failed divisions. Similar defects had been reported in *rad18Δ* cells in response to low doses of chronic damage (Hishida *et al*, 2009).

### Rad53 is required during S phase, but not for postreplicative gap filling

**Figure 3:**
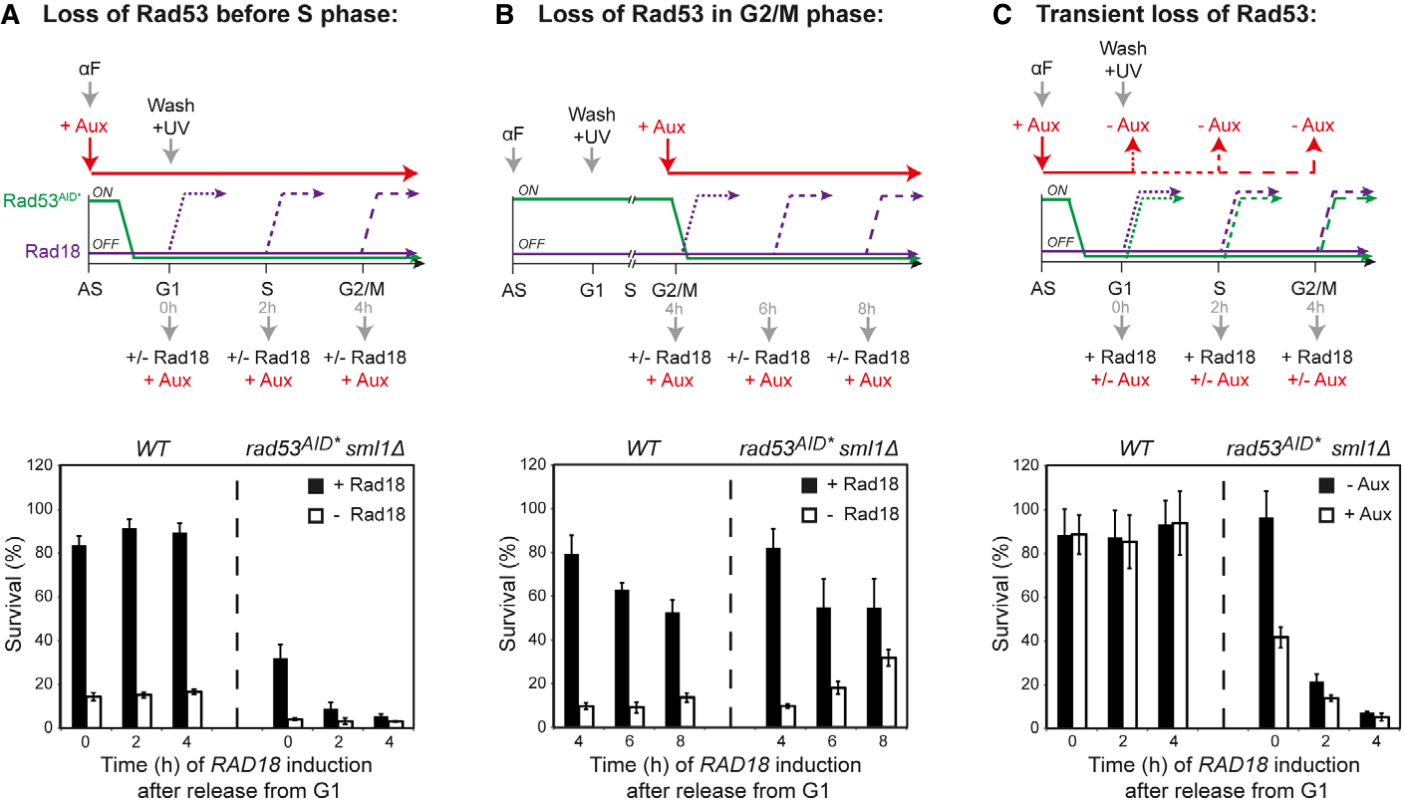
Rad53 is required during the S phase that precedes DNA damage bypass Loss of Rad53 before S phase: recovery assays upon *RAD18* induction were performed as described in Fig 1B, but Rad53^AID*−9myc^ degradation was induced by adding auxin during synchronization.Loss of Rad53 in G2/M phase: assays were performed as above, but Rad53^AID*−9myc^ degradation was induced 4 h after release into S phase.Transient loss of Rad53: recovery was measured after Rad53^AID*−9myc^ degradation during synchronization and re‐expression together with *RAD18* at the indicated times during the cell cycle.Data information: (A–C) Error bars indicate SD derived from at least three independent experiments.

The failure of *rad53Δ* mutants to reactivate damage bypass might be due to a direct requirement of Rad53 for the filling of daughter‐strand gaps. However, the successive loss of chromosome integrity in the course of S phase suggested an essential function of checkpoint signaling already at the stage where the gaps emerge. In order to distinguish between these models, we used a previously characterized allele, *rad53* ^AID*−9myc^, that encodes the kinase as a fusion with an auxin‐inducible degron (Morawska & Ulrich, 2013). This allows depletion of the protein within < 1 h and confers a *rad53Δ*‐like phenotype in the presence, but *WT* behavior in the absence of auxin (Morawska & Ulrich, 2013; Appendix Fig S1A). With this allele, we re‐examined the recovery of viability under conditions where Rad53^AID*−9myc^ was depleted either prior to the start of S phase (Fig 3A) or after passage through S phase, but before reactivation of Rad18 (Fig 3B). As expected, when Rad53^AID*−9myc^ was removed prior to UV treatment, recovery was strongly compromised (Fig 3A). The defect was not as severe as in a *rad53Δ* strain, but this may have been due to residual protein even in the presence of auxin. Recovery was normal in the absence of auxin (Appendix Fig S1B), indicating that the failure to restore viability was indeed a consequence of the degradation of Rad53, and the AID*‐tagged protein was functional under stabilizing conditions. Degradation of Rad53^AID*−9myc^ after completion of S phase had no detrimental effect on viability, suggesting that Rad53 function is dispensable for damage bypass in G2/M (Fig 3B and Appendix Fig S1C and D).

We then set up an experiment where Rad53^AID*−9myc^ was temporarily degraded before release from G1 but re‐expressed together with Rad18 at different times during the cell cycle (Fig 3C, ‐Aux). When *rad53* ^AID*−9myc^ was re‐expressed before entry into S phase (0 h), cells recovered viability. However, if re‐expression was postponed to mid‐S (2 h) or G2 phase (4 h), loss of viability became irreversible, even though recovery of Rad53^AID*−9myc^ protein levels resulted in a restoration of checkpoint signaling (assessed by the upregulation of the ribonucleotide reductase subunit, Rnr4; Appendix Fig S1E). Taken together, these data indicate that a transient loss of Rad53 during replication is sufficient to irreversibly prevent productive damage bypass.

### Rad53‐mediated inhibition of Exo1 and Pif1 maintains bypass competence during S phase

**Figure EV3:**
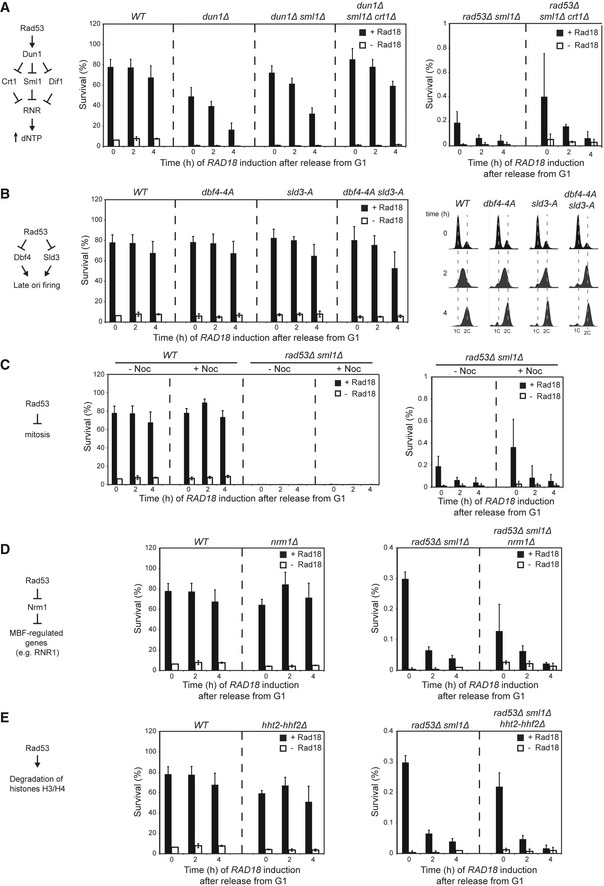
Analysis of downstream targets of Rad53 Recovery assays were performed according to the scheme in Fig 1B, by releasing UV‐irradiated G1 cells into S phase in the absence of Rad18 and inducing *Tet‐RAD18* expression at the indicated times. 
In response to replication stress, Rad53 upregulates dNTP production through phosphorylation of Dun1, which in turn targets the transcriptional repressor Crt1 and the protein inhibitors Sml1 and Dif1, thereby relieving ribonucleotide reductase (RNR) inhibition and boosting dNTP levels (Hustedt *et al*, 2013) (left). Deletion of *DUN1* reduces the capacity of cells to recover, and this effect is suppressed partially by deletion of *SML1* and almost completely by a concomitant deletion of *SML1* and *CRT1* (middle). Hence, upregulation of dNTP levels is required for efficient damage bypass. However, *sml1Δ* and *crt1Δ* do not compensate for loss of *RAD53* (right). Therefore, upregulation of dNTP levels is required, but not sufficient to maintain bypass competence.In response to replication stress, Rad53 inhibits late origin firing through phosphorylation of Dbf4 and Sld3 (Lopez‐Mosqueda *et al*, 2010; Zegerman & Diffley, 2010) (left). Cells expressing non‐phosphorylatable alleles of *DBF4* and *SLD3* (*dbf4‐4A sld3‐A*) (Zegerman & Diffley, 2010) recover virtually as efficiently as *WT* cells (middle), even though they progress through S phase as rapidly as the *rad53Δ* mutant (right). Hence, accelerated progression through S phase or firing of late origins does not preclude efficient postreplicative gap filling.Delay of mitosis by nocodazole treatment is insufficient to restore viability in *rad53Δ*. Middle: recovery assays performed with or without addition of nocodazole (added twice: 15 μg/ml at 0 h and 10 μg/ml at 2 h). Right: magnification of the graphs showing *rad53Δ*.Rad53 is responsible for induction of a set of MBF‐regulated genes during S phase in response to DNA damage, mediated via inactivation of the transcriptional co‐repressor Nrm1 (left) (Travesa *et al*, 2012). Hence, *NRM1* deletion suppresses *rad53Δ*‐associated lethality (de Bruin *et al*, 2006). However, *nrm1Δ* does not restore Rad18‐dependent recovery of viability in *rad53Δ* (right), indicating that Nrm1 is not a relevant target of Rad53 in this context.Rad53 is required for degradation of excess histones upon DNA damage (Gunjan & Verreault, 2003) (left). Reduction in histone gene dosage therefore suppresses HU and MMS sensitivities of *rad53Δ* (Gunjan & Verreault, 2003). However, reducing histone dosage by deletion of *HHT2* and *HHF2* does not restore damage bypass competence in a *rad53Δ* background (right), thus excluding histone dosage as a critical factor.Data information: (A–E) Error bars indicate SD derived from three independent experiments.

**Figure EV4:**
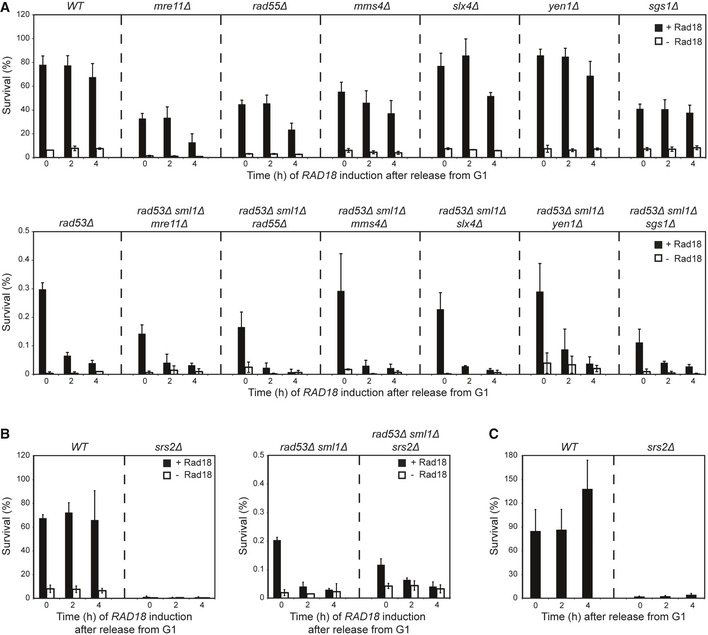
Loss of DNA damage bypass competence in *rad53Δ* is not due to excessive homologous recombination Rad18‐dependent recovery assays were performed according to the scheme in Fig 1B. 
Recovery of viability in strains defective in distinct stages of homologous recombination: *mre11Δ* (affecting DNA resection), *rad55Δ* (disabled in the formation of recombinogenic filaments), *mms4Δ*,* slx4Δ*,* yen1Δ,* and *sgs1Δ* (defective in the resolution or dissolution of joint molecules). Top: control assays performed in a *RAD53* background. Bottom: recovery assays of recombination mutants in combination with *rad53Δ*.Recovery assays of *srs2Δ* (left) and *srs2Δ rad53Δ* (right) indicate that elevated homologous recombination in the *srs2Δ* mutant (Aguilera & Klein, 1988) does not rescue the recovery defect of *rad53Δ*.Poor survival of UV‐irradiated *srs2Δ* cells grown in the continuous presence of Rad18 indicates that the recovery defect in the *srs2Δ* background is independent of Rad18.Data information: (A–C) Error bars indicate SD derived from three independent experiments.

In order to identify the mechanism(s) by which Rad53 maintains damage bypass competence, we systematically examined possible contributions of Rad53's downstream targets. We found that upregulation of dNTP levels was required for efficient damage bypass, but not sufficient to restore viability in *rad53Δ* cells (Fig EV3A). Abolishing the suppression of late origin firing (Lopez‐Mosqueda *et al*, 2010; Zegerman & Diffley, 2010) in a Rad53‐proficient background strongly accelerated progression through S phase, but did not interfere with viability (Fig EV3B). *Vice versa*, delay of mitosis by nocodazole treatment did not rescue viability in the absence of Rad53 (Fig EV3C). We were also able to exclude a contribution of Rad53‐induced gene expression controlled by the transcriptional co‐repressor Nrm1 (de Bruin *et al*, 2006; Travesa *et al*, 2012; Fig EV3D) and an influence of histone gene dosage, which had also been shown to affect the damage sensitivity of *rad53Δ* mutants (Gunjan & Verreault, 2003; Fig EV3E). Finally, in order to assess whether elevated homologous recombination was the underlying cause of the problems or rather a reflection of (unsuccessful) attempts at repair, we analyzed the effects of various mutants defective in distinct stages of homologous recombination, such as *mre11Δ*,* rad55Δ*,* mms4Δ*,* slx4Δ*,* yen1Δ, sgs1Δ,* and *srs2Δ*. However, none of them restored Rad18‐mediated survival in a *rad53Δ* background (Fig EV4).

**Figure 4:**
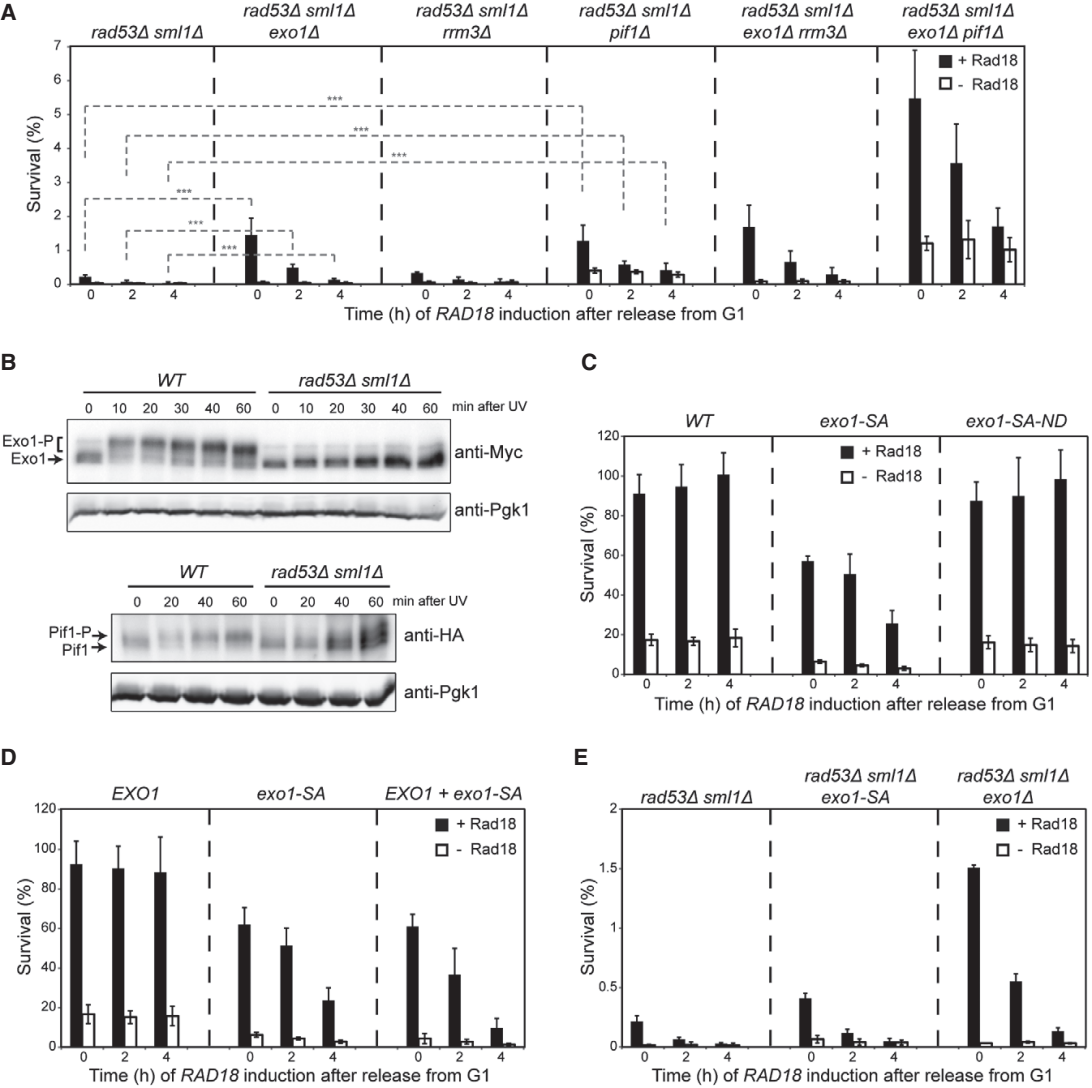
Checkpoint‐mediated inhibition of Exo1 and Pif1 activity is required to maintain bypass competence Recovery of viability upon *RAD18* induction in the indicated strains, measured as described in Fig 1B (***P < 0.001).Exo1 and Pif1 phosphorylation in the indicated strains, released into S phase after UV irradiation in the absence of Rad18. Exo1^9myc^ and Pif1^6HA^ were detected by Western blotting. Pgk1 served as loading control.Recovery of viability in *exo1* mutants (SA: dephospho‐mimicking; ND: nuclease‐dead).Recovery of viability in strains harboring wild‐type *EXO1*,* exo1‐SA* mutant or both alleles (*EXO1 *+* exo1‐SA*).Recovery of viability in *rad53Δ exo1‐SA*.Data information: (A, C–E) Error bars indicate SD derived from at least three independent experiments. Significance in panel (A) was calculated by the Student's *t‐*test.Source data are available online for this figure.

**Figure EV5:**
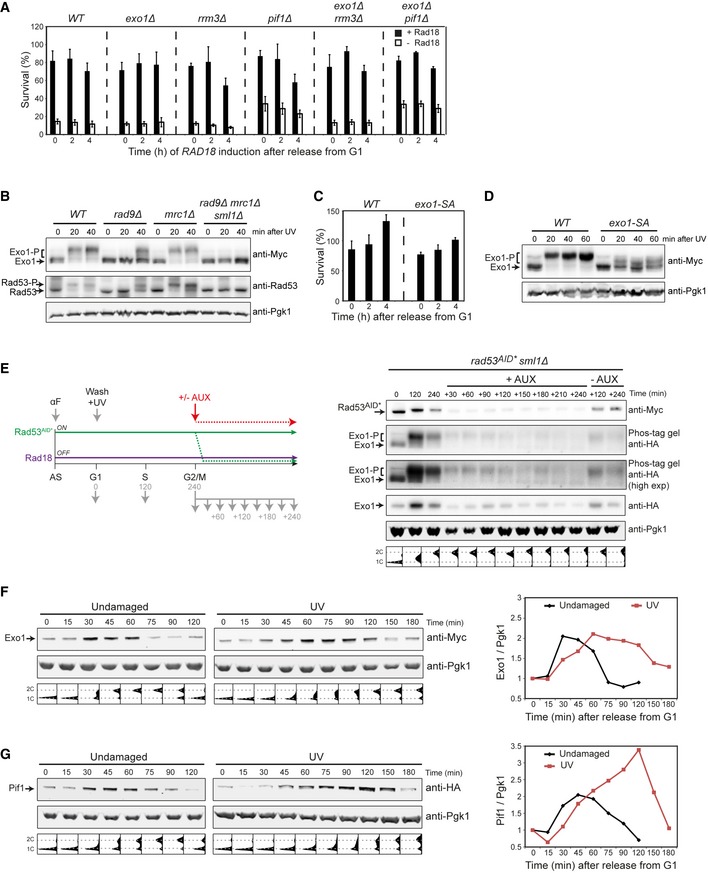
Exo1 and Pif1 are controlled by the DNA damage checkpoint and the cell cycle As a control for the experiment shown in Fig 4A, recovery of viability upon *RAD18* induction was measured in the indicated mutants in a *RAD53* background.Exo1 and Rad53 phosphorylation is largely dependent on Rad9, with Mrc1 acting as a backup. Phosphorylation was assayed in the indicated strains upon release into S phase after UV irradiation (20 J/m^2^) in the absence of Rad18. Exo1^9myc^ and Rad53 were detected by Western blotting. Pgk1 served as loading control.As a control for the experiment shown in Fig 4C, survival of UV‐irradiated *exo1‐SA* was measured in the continuous presence of Rad18.Exo1 phosphorylation is not completely abolished in *exo1‐SA*. Exo1 phosphorylation was assayed in *WT* and *exo1‐SA* cells released into S phase after UV irradiation in the absence of Rad18.Exo1 levels decline in a Rad53‐independent manner in G2/M, but without significant dephosphorylation. Left: experimental scheme; right: time course of Rad53^AID*−9myc^ and Exo1^6HA^ phosphorylation and protein levels in *rad53* ^AID*−9myc^ cells released into S phase after UV irradiation in the absence of Rad18. At 4 h after release, Rad53^AID*−9myc^ degradation was induced by adding auxin to part of the culture. Pgk1 was used as loading control. Cell cycle profiles are shown at the bottom.Time course of Exo1^9myc^ levels in *WT* cells released into S phase in the absence of damage or after UV irradiation. Re‐entry into the next S phase was prevented by adding αF after 60 or 90 min, respectively. Exo1^9myc^ protein levels, relative to G1 (0 min) and normalized to the Pgk1 signal, are plotted on the right.Time course of Pif1^6HA^ levels in *WT* cells performed as in panel (F). Pif1^6HA^ protein levels, relative to G1 (0 min) and normalized to the Pgk1 signal, are plotted on the right.Data information: (A, C) Error bars indicate SD derived from three independent experiments.Source data are available online for this figure.

Having excluded recombination as a source of genome instability, we considered pathological expansion of ssDNA as a cause of the observed chromosome damage. At HU‐stalled replication forks, the excessive formation of ssDNA and fork breakdown that is observable in checkpoint mutants is mainly promoted by Exo1, Pif1, and Rrm3 (Cotta‐Ramusino *et al*, 2005; Rossi *et al*, 2015). The latter two also contribute to unperturbed replication by resolution of problematic sequences and DNA–protein complexes, respectively (Ivessa *et al*, 2003; Paeschke *et al*, 2011; Sabouri *et al*, 2012), and all of them are inhibited by Rad53‐mediated phosphorylation (Smolka *et al*, 2007; Morin *et al*, 2008; Rossi *et al*, 2015). Consistent with a contribution to daughter‐strand gap erosion, deletion of *EXO1* or *PIF1* significantly improved viability of *rad53Δ* mutants in our recovery assay (Fig 4A), while they had little influence in a checkpoint‐proficient background (Fig EV5A). A combination of *exo1Δ* and *pif1Δ* was even more effective, although it should be noted that recovery was still far from complete. In contrast, *rrm3Δ* did not affect recovery alone or in combination with *exo1Δ* (Fig 4A). Accordingly, we found Exo1 and Pif1 to be phosphorylated in a Rad53‐dependent manner after UV irradiation and release into S phase (Fig 4B). Phosphorylation of Exo1 followed the pattern observed for Rad53 itself under these conditions, that is, it was mediated mainly via Rad9, with Mrc1 playing a backup role only in the absence of Rad9 (Fig EV5B).

If Rad53's predominant role in maintaining damage bypass competence were indeed the suppression of Exo1 and Pif1, a Rad53‐insensitive Exo1 should confer a recovery defect comparable to a checkpoint mutant in our assay. Consistent with this prediction, replacement of *EXO1* by a previously described allele, *exo1‐SA*, where the four major phosphorylation sites were mutated to alanine (Morin *et al*, 2008; Doerfler & Schmidt, 2014), considerably reduced the capacity to restore viability (Fig 4C). This effect was not primarily a consequence of a general damage sensitivity of the *exo1‐SA* mutant because in the presence of Rad18, survival was similar to *WT* (Fig EV5C). The defect conferred by *exo1‐SA* was dependent on the protein's nuclease activity, since its inactivation (in *exo1‐SA‐ND*) restored *WT* levels of survival (Fig 4C). Moreover, the Exo1‐SA protein had a dominant effect (Fig 4D), and combination with *rad53Δ* did not exacerbate the situation, indicating an epistatic relationship (Fig 4E). However, the phenotype of the *exo1‐SA* mutant was significantly milder than that of *rad53Δ*. While this may imply additional Rad53 targets involved in controlling the stability of postreplicative gaps, for example, Pif1, part of the effect could also be due to a phosphorylation of Exo1 at additional sites not covered by the *exo1‐SA* allele (Morin *et al*, 2008). Indeed, analysis of the mutant protein revealed residual phosphorylation (Fig EV5D).

The notion that Rad53 restricts Exo1 and Pif1 activity can explain why checkpoint signaling is essential during replication when damage bypass is delayed, but it does not account for the observation that daughter‐strand gaps remain bypass‐competent for extended periods if Rad53 is depleted after cells have reached G2/M phase (Fig 3B, 6 h and 8 h). When we monitored the state of Exo1 tagged with an HA‐epitope in *rad53* ^AID*−9myc^, we noted that—independent of the presence or absence of Rad53—protein levels rapidly declined at the end of S phase (Fig EV5E). This was neither due to our experimental set‐up nor a consequence of the HA‐tag, as an Exo1^9myc^ protein exhibited similar, damage‐independent fluctuation along the cell cycle in an otherwise unmodified strain, with protein levels peaking in S phase and declining in G2/M (Fig EV5F). The same pattern was observed for Pif1 (Fig EV5G). Thus, the cell cycle regulation of Exo1 and Pif1 apparently obviates the need for Rad53‐mediated inhibition in G2/M, and inactivation of Rad53‐dependent damage signaling at this stage can therefore no longer interfere with productive damage bypass (Fig 3B). In addition, Exo1 dephosphorylation after degradation of Rad53 proceeded very slowly (Fig EV5E), which might contribute to a sustained repression of any Exo1 activity remaining in G2/M.

### Damage signaling during S phase requires Exo1 activity at daughter‐strand gaps

**Figure 5:**
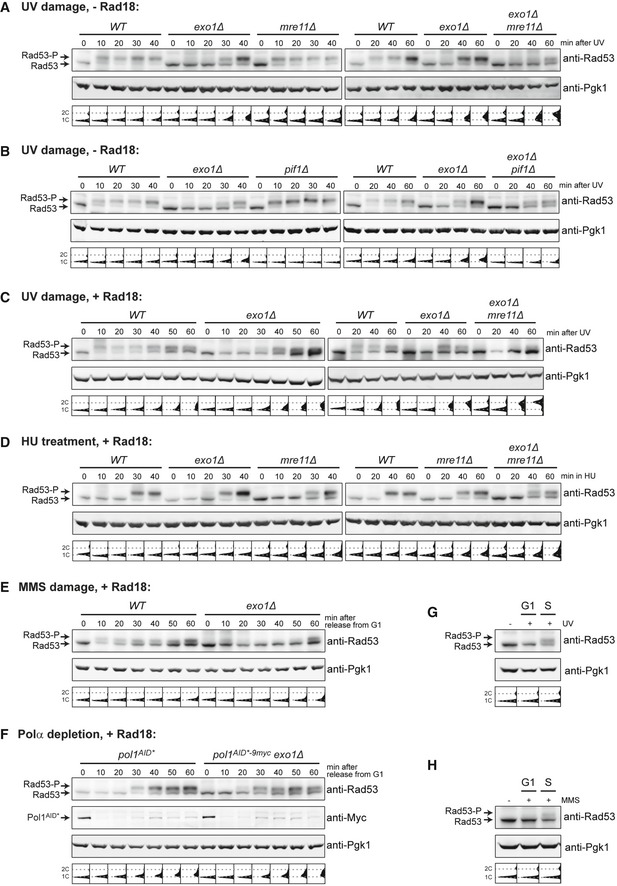
Exo1 is required for robust checkpoint activation in response to polymerase‐blocking lesions but not replisome‐induced fork stalling Rad53 phosphorylation in the indicated strains, synchronized in G1, UV‐irradiated (20 J/m^2^), and released into S phase in the absence of Rad18. Pgk1 served as loading control. Cell cycle profiles are shown below the blots.Rad53 phosphorylation in the indicated strains, treated as in panel (A).Rad53 phosphorylation in the indicated strains, treated as in panel (A) but grown in the presence of Rad18.Rad53 phosphorylation in the indicated strains, synchronized in G1, and released into S phase in medium containing HU (120 mM).Rad53 phosphorylation in the indicated strains, synchronized in G1, treated with MMS (0.08%) for 30 min, and released into S phase.Rad53 phosphorylation in the indicated strains, synchronized in G1, and released into S phase. Auxin was added 30 min prior to release for induction of Pol1^AID*−9myc^ degradation.Rad53 phosphorylation in *WT* cells, synchronized in G1, undamaged, or UV‐irradiated (20 J/m^2^) and either maintained in G1 or released into S phase for 20 min.Rad53 phosphorylation in *WT* cells, synchronized in G1, untreated or treated with 0.08% MMS for 30 min, and either maintained in G1 or released into S phase for 10 min. Source data are available online for this figure.

At DSBs and NER gaps outside of S phase, Exo1 is subject to a Rad53‐dependent feedback regulation where Exo1 itself generates the checkpoint signal that ultimately restricts its own activity (Nakada *et al*, 2004; Dewar & Lydall, 2010; Giannattasio *et al*, 2010). If such phenomenon also applied at daughter‐strand gaps during S phase, deletion of *EXO1* should interfere with Rad53 activation in our assay. Indeed, when cells were synchronized, UV‐irradiated, and released into S phase in the absence of Rad18, phosphorylation of Rad53 was significantly delayed in *exo1Δ* cells, resulting in faster cell cycle progression (Fig 5A). Under these conditions, Mre11 can apparently compensate for the lack of Exo1 to some extent, as *mre11Δ* cells activated Rad53 like *WT*, but the *exo1Δ mre11Δ* double mutant exhibited a further delay in Rad53 phosphorylation and a severely accelerated S phase. Compared to Exo1, Pif1 contributed much less to checkpoint activation in S phase (Fig 5B). These observations strongly suggest that Exo1‐mediated resection generates the signal for timely activation of the checkpoint at daughter‐strand gaps accumulating in the absence of Rad18.

The observed relationship between Exo1 and Rad53 at daughter‐strand gaps prompted us to examine the mechanism of checkpoint activation in damage bypass‐competent cells. As shown in Fig 5C, despite an overall reduced signal, Rad53 activation after UV irradiation followed the same pattern in the presence of Rad18 as in its absence, with a strong dependence on Exo1 and a minor contribution of Mre11 at later time points. Notably, such relationship did not apply when replication fork stalling was induced by HU treatment, which presumably impinges on replisome progression without generating lesions (Fig 5D). In this situation, Rad53 was efficiently activated in an Exo1‐independent, partially Mre11‐dependent manner, consistent with previous reports (Nakada *et al*, 2004). We therefore asked under which conditions of replication stress Exo1 would contribute to Rad53 activation. MMS, which causes polymerase stalling due to lesions in the replication template, induced Rad53 phosphorylation in an Exo1‐dependent manner, as observed with UV (Fig 5E, Appendix Fig S2). As an alternative, template‐independent source of replication stress, we depleted polymerase α (Pol1) in G1‐arrested cells by means of an auxin‐inducible degron tag (Morawska & Ulrich, 2013). Subsequent release into S phase caused a strong spontaneous checkpoint response in *pol1* ^AID*−9myc^ cells, most likely because of a problem with initiating lagging strand synthesis (Fig 5F). In this situation, Rad53 phosphorylation was independent of Exo1, as observed in response to HU. A replication‐independent checkpoint activation, for example, by NER gaps, was ruled out by the lack of UV‐ or MMS‐induced Rad53 phosphorylation in cells maintained in G1 phase (Fig 5G and H).

### DNA lesions and replisome problems activate checkpoint signaling via distinct structures

**Figure 6:**
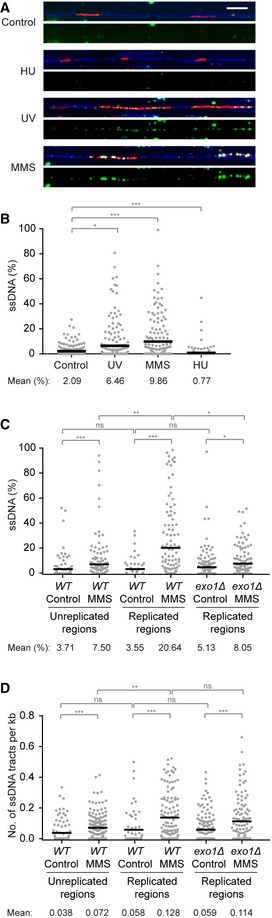
ssDNA accumulates within tracts of newly synthesized DNA in response to polymerase‐blocking lesions DNA fibers, prepared by combing of genomic DNA isolated from cells synchronized in G1 and released into S phase in the presence of EdU (added 15 min before release). Cells were harvested 20 min after release for control; 30 min after UV irradiation (20 J/m^2^); 30 min after treatment with 0.04% MMS for 30 min prior to release; and 60 min after release into 120 mM HU. Fibers were stained with YOYO‐1 for total DNA (blue). EdU incorporation was visualized by a click reaction with Alexa Fluor 647 (red), and ssDNA was detected by means of an antibody (green). Scale bar = 10 kbp.Quantification of the fraction of ssDNA within newly replicated DNA, determined for individual EdU‐stained tracts by measuring total tract length and total length of ssDNA within that tract. Evidence for EdU‐stained regions representing replication tracts is shown in Appendix Fig S3D and E. Number of replication tracts analyzed: Control = 186; UV = 204; MMS = 168; HU = 198.Quantification of the fraction of ssDNA within or outside of EdU‐stained replication tracts derived from *WT* or *exo1Δ* cells, determined as above. G1‐arrested cells were incubated with or without 0.02% MMS for 30 min and released into EdU for 30 min. Number of replication tracts analyzed: *WT* control = 63; *exo1Δ* control = 151; *WT* MMS = 124; *exo1Δ* MMS = 122. Number of EdU‐negative tracts analyzed: *WT* control = 96; *WT* MMS = 171.Density of ssDNA tracts within or outside of individual replication tracts from the experiment shown in panel (C), calculated by dividing the number of ssDNA tracts by the length (in kb) of the corresponding EdU‐stained or EdU‐negative region.Data information: (B–D) Significance was calculated by the Mann–Whitney test (ns: not significant; *P < 0.05; **P < 0.01; ***P < 0.001). Black bar = mean.

Our observations imply that replication‐dependent, lesion‐induced Rad53 activation follows a similar Exo1‐mediated mechanism as the replication‐independent process at NER gaps. This strongly suggests that even in damage bypass‐competent cells the signal that activates Rad53 in response to DNA damage emanates from daughter‐strand gaps and not from stalled replication forks. In order to obtain direct evidence for this model, we visualized the distribution of ssDNA relative to regions of newly replicated DNA on fibers isolated from early S phase cells under conditions of replication stress and calculated the total percentage of ssDNA along each replication tract (Fig 6A and B). Consistent with an accumulation of daughter‐strand gaps, we found elevated levels of ssDNA along the lengths of many replication tracts on DNA fibers from UV‐ or MMS‐treated cells. This pattern was strikingly different when fork stalling was induced by nucleotide depletion, irrespective of HU concentration (Fig 6A and B, Appendix Fig S3A–C). In a second experiment, we compared the MMS‐induced enrichment of ssDNA within replicated regions to the corresponding enrichment in the EdU‐negative, that is, unreplicated areas in the same set of fibers. Outside the replication tracts, MMS treatment caused an approximately twofold increase in the total percentage of ssDNA, largely reflecting a higher density of tracts (Fig 6C and D). Within the EdU‐labeled regions, tract density was also twofold higher, but the total amount of ssDNA was enriched by almost sixfold. Hence, the accumulation of ssDNA in the newly replicated DNA is mainly attributable to an increase in tract length (Fig 6C and D). These results suggest that the majority of ssDNA within replicated areas corresponds to postreplicative daughter‐strand gaps, and only a minority may have resulted from replication‐independent processes such as an expansion of NER gaps.

In an *exo1Δ* mutant, the fraction of ssDNA upon MMS treatment was more than twofold reduced compared to *WT* cells, while the number of tracts per unit length was similar, indicating that Exo1 indeed influences the length of the tracts, but not their incidence (Fig 6C and D). Taken together, our data reveal two distinct mechanisms by which replication stress can generate a checkpoint signal: a fork‐associated, Exo1‐independent mechanism that responds to replisome problems or nucleotide depletion, and an Exo1‐and Rad9‐dependent process that is induced by lesions in the replication template and emanates from postreplicative daughter‐strand gaps.

## Discussion

### Lesion‐induced replication stress is perceived behind the fork

Numerous studies have addressed the question of how replication stress is sensed and converted to a global checkpoint signal (Branzei & Foiani, 2009). The general concept that has emerged from these studies postulates that checkpoint kinases are activated by extended regions of ssDNA arising at stalled forks from an uncoupling between replicative helicase and DNA polymerase movement or between leading and lagging strand synthesis, and that checkpoint signaling maintains genome stability by stabilizing such structures (Byun *et al*, 2005; Branzei & Foiani, 2009). A number of reports have highlighted important differences in the mechanism by which different agents activate replication‐specific checkpoint signaling. For example, Segurado and Diffley (Segurado & Diffley, 2008) have described two ways of replication fork stabilization by Rad53: an Exo1‐dependent pathway that mainly reacts to MMS, UV or ionizing radiation damage, and an Exo1‐independent mechanism that is more important after exposure to HU. Similarly, Nielsen *et al* (2013) have proposed that an HU‐stalled fork adopts a structure distinct from one that is stalled by MMS, consistent with the notion that the replisome component Mrc1 dominates HU‐induced checkpoint signaling, whereas the response to MMS depends on Rad9 (Alcasabas *et al*, 2001; Crabbe *et al*, 2010; Nielsen *et al*, 2013). Yet, the stalled replication fork as the origin of the checkpoint signal in response to replication stress has not been called into question.

**Figure 7:**
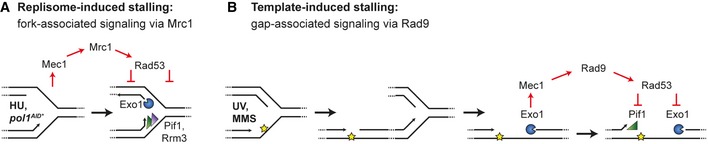
Model for differential checkpoint activation in response to replisome problems and DNA lesions Upon replisome stalling—after HU treatment or Pol1 degradation—an excess of ssDNA at arrested forks activates Rad53 via Mrc1, which prevents replication fork breakdown by inhibition of Exo1, Pif1 and Rrm3 activities.Replication through damaged DNA—after UV irradiation or MMS treatment—generates daughter‐strand gaps behind replication forks due to re‐priming events, thereby reducing the exposure of ssDNA at forks. Exo1‐mediated expansion of daughter‐strand gaps is then required for robust Rad9‐dependent Rad53 activation, which in turn leads to Exo1 and Pif1 inhibition by phosphorylation.

We now suggest that the two pathways of sensing replication stress not only differ in their dependence on Mrc1 versus Rad9, but also with respect to their origin (Fig 7). In line with previous models, the HU‐responsive, Mrc1‐dependent pathway primarily monitors the state of the replisome and is thus closely associated with the fork structure itself (Fig 7A). However, we propose that the Rad9‐dependent pathway that mediates the response to lesions in the replication template originates not from the fork, but rather from daughter‐strand gaps, left behind after passage of the replisome and expanded by the action of Exo1 in order to generate a sufficient checkpoint signal (Fig 7B). The notion that an establishment of active replication forks is required for Rad53 activation in response to damaging agents such as MMS (Tercero *et al*, 2003) does not contradict this concept, as passage of a replication fork would be a prerequisite for the accumulation of gaps. Moreover, although our model originates from observations made under conditions where a delay of damage bypass exacerbates the consequences of checkpoint failure, we found that the same mechanism applies in damage bypass‐competent cells. Finally, the idea of Rad9‐dependent checkpoint activation by regions of ssDNA embedded in a chromatinized environment at some distance from the replication fork is consistent with the mechanism of Rad9 recruitment by binding to Lys79‐methylated histone H3 (Wysocki *et al*, 2005). Brown and coworkers have recently described an assembly of a signaling complex in discrete domains behind replication forks that leads to activation of Mec1 in response to MMS (Balint *et al*, 2015), and our recent observation that ubiquitylation of histone H2B at Lys123, a prerequisite for efficient H3 methylation, contributes to postreplicative damage bypass, also supports this notion (Hung *et al*, 2017). Thus, our results complement published data and offer a satisfying mechanistic explanation for the differences between replisome‐ and template‐induced checkpoint activation.

### Re‐priming as a means to resolve replication fork uncoupling

The model posits that lesions in the template strand do not pose a significant block to the overall progression of the replisome, but rather induce downstream re‐priming of DNA synthesis, thus giving rise to daughter‐strand gaps. Indeed, re‐priming has been well documented in various experimental systems (Heller & Marians, 2006; Lopes *et al*, 2006; Callegari *et al*, 2010; Hashimoto *et al*, 2010; Elvers *et al*, 2011). Thus, if damage signaling were responsive only to fork‐associated ssDNA, efficient re‐priming should work against activation of the Mrc1‐mediated checkpoint, which requires a critical number of arrested forks (Shimada *et al*, 2002; Tercero *et al*, 2003). Gap‐associated checkpoint activation via Rad9 would overcome this problem. Our model is therefore at odds with the established concept of replication fork uncoupling that has been proposed to explain the origin of the ssDNA required for checkpoint activation in response to both polymerase inhibition and UV‐ or cis‐platinum‐induced lesions (Walter & Newport, 2000; Byun *et al*, 2005). It has to be noted that that model, derived from experiments in *X. laevis* egg extracts, relied on the detection of ssDNA in a plasmid template as a readout without specifying whether the ssDNA accumulated at or behind the fork. Indeed, in a similar set‐up Hashimoto *et al* (2010) readily observed daughter‐strand gaps, but very little strand uncoupling in replicating MMS‐damaged sperm DNA by means of electron microscopy. We would like to point out, however, that at severe damage load, re‐priming may eventually become inefficient due to an extremely high density of lesions, such that checkpoint signaling could then become fork‐associated even in response to UV or MMS. In support of this view, even moderate doses of MMS have been reported to inhibit late origin firing in an Mrc1‐dependent manner, suggesting that the replisome is affected to some extent by damaged templates, even though this may not compromise viability (Hang *et al*, 2015).

### Exo1 contributes to checkpoint activation in response to damage‐induced replication stress

Exo1 is well known for its contribution to checkpoint activation by mediating resection of 5′‐termini or widening of NER gaps outside of S phase (Dewar & Lydall, 2010; Giannattasio *et al*, 2010). In both cases, Rad53 limits Exo1 activity by a negative feedback control. We now show that a comparable situation applies to UV‐ or MMS‐induced checkpoint signaling in S phase cells, raising the question whether the phenomenon observed here is actually replication‐dependent. However, as also reported by others (Tercero *et al*, 2003), at the low doses of MMS applied here, Rad53 phosphorylation strictly depended on the initiation of S phase, thus ruling out a replication‐independent mechanism. The same had previously been shown for both *WT* and *rad14Δ* cells in response to UV, arguing against an involvement of NER (Neecke *et al*, 1999). Finally, we observed DSBs only under conditions where both damage bypass and checkpoint signaling were compromised (Fig 2A). Hence, we conclude that the features giving rise to damage signaling in our system are neither DSBs nor NER gaps, but replication‐dependent structures.

Yet, the function of Exo1 observed here appears to be distinct from its fork‐associated roles: at HU‐stalled or collapsed forks, Exo1 does not contribute to damage signaling or replication restart and may even cause fork breakdown (Cotta‐Ramusino *et al*, 2005; Segurado & Diffley, 2008; Tsang *et al*, 2014). In contrast, in response to UV or MMS damage, Exo1 mediates Rad53 activation. Moreover, previous reports indicate that DNA damage bypass by template switching initiates from internal tracts of ssDNA rather than free termini, and Exo1 itself participates in this process, likely by expanding them in preparation for strand invasion (Vanoli *et al*, 2010; Karras *et al*, 2013; Giannattasio *et al*, 2014). These findings support our model, and they also offer an explanation for why deletion of *EXO1* in a checkpoint‐deficient background did not result in full recovery of damage bypass competence in our system.

### Controlling nuclease activity during replication

Our data suggest that uncontrolled Exo1 and to some extent Pif1 activity is largely responsible for the catastrophic loss of viability upon entry into S phase when both checkpoint signaling and damage bypass are inactivated. This raises the question of what structures need to be protected by the checkpoint in order to prevent chromosome fragmentation and lethality during replication of damaged templates. We cannot formally exclude collapsed forks as the origin of lethality, as Rad18 may normally operate both at daughter‐strand gaps and “on‐the‐fly”, that is, directly at the replication fork. However, we favor a scenario where checkpoint signaling is required primarily to maintain the integrity of daughter‐strand gaps by preventing excessive resection (Fig 7B), because the synthetic lethality between checkpoint and damage bypass defects applies only to DNA damage, but not to HU‐mediated fork problems and is observable only with *rad9Δ*, but not with *mrc1Δ* mutants. Moreover, daughter‐strand gaps are the structures known to hyper‐accumulate in the absence of damage bypass. Hence, it appears likely that the erosion of such structures is responsible for the loss of viability in our assay. A possible mechanism by which daughter‐strand gaps could give rise to DSBs would be the merger of an expanding gap with a nick on the parental strand, possibly in the context of a nucleotide or base excision repair intermediate. Both damage bypass (via gap filling) and checkpoint signaling (via inhibition of gap expansion) are expected to counteract such events, which would explain why we observed DSBs only under conditions where both pathways are inactive. Alternatively, extended regions of ssDNA may simply be more vulnerable to attack by endonucleases or other endogenous sources of damage.

A dependence of viability on Rad9‐mediated checkpoint signaling in damage bypass‐deficient cells has also been noted in response to chronic low‐dose exposure to genotoxic agents (Hishida *et al*, 2009; Huang *et al*, 2013). This phenomenon was attributed to an accumulation of daughter‐strand gaps and even checkpoint‐proficient cells gradually lost viability over time after such treatment. Deletion of *EXO1* mitigated the effect to some extent, again arguing that control over Exo1 activity is particularly important to prevent erosion of daughter‐strand gaps.

Intriguingly, another nuclease—Mre11—has been implicated in the degradation of nascent DNA in vertebrate systems (Hashimoto *et al*, 2010; Schlacher *et al*, 2011). Here, protection of regressed forks or gap structures from Mre11‐mediated resection was found to depend on homologous recombination factors such as BRCA1 and Rad51. While fork regression has not been observed in checkpoint‐competent yeast cells, a spontaneous accumulation of daughter‐strand gaps was also found in budding yeast *rad52Δ* and *rad51Δ* mutants (Hashimoto *et al*, 2010). It is possible that the aggravation of the *exo1Δ* checkpoint defect that we observed upon deletion of *MRE11* is attributable to the same phenomenon.

In addition to Rad53‐mediated inhibition of its activity, we found Exo1—like Pif1—to be strongly cell cycle‐regulated. Thus, the nuclease apparently needs to be carefully controlled in order to balance its beneficial versus potentially detrimental actions. This may reflect the necessity to prevent homologous recombination in G1, but another intriguing possibility is that Exo1 regulation could contribute to controlling the balance between damage processing via template switching versus translesion synthesis: its downregulation at the end of S phase might inhibit template switching by preventing gap expansion, thus further promoting the temporal separation of the two pathways that is already suggested by the staggered expression patterns of Rad5 and Rev1 (Waters & Walker, 2006; Ortiz‐Bazan *et al*, 2014).

Our observations demonstrate that the interplay between Rad53 and Exo1, ensuring robust checkpoint activation while at the same time protecting regions of ssDNA from harmful expansion, constitutes an important and very general aspect of the cellular damage response, promoting genome maintenance by such diverse pathways as homologous recombination at DSBs, long‐patch NER and postreplicative damage bypass.

## Materials and Methods

### Yeast strains and growth conditions

All yeast strains are listed in Appendix Table S1. The doxycycline‐repressible *Tet‐RAD18* construct has been described previously (Daigaku *et al*, 2010). Strains carrying gene deletions or epitope‐tagged alleles were created by PCR‐based methods or by mating and tetrad dissection. Degron‐tagged alleles of *RAD53* and *POL1* were constructed as described (Morawska & Ulrich, 2013). All strains carrying a deletion or degron‐tagged allele of *RAD53,* deletion of *MEC1* or deletion of both *RAD9* and *MRC1* were constructed in an *sml1Δ* background. Strains carrying *exo1‐SA* and *exo1‐SA‐ND* alleles were created as described previously (Doerfler & Schmidt, 2014). To test for dominance, the *exo1‐SA* allele containing its own promoter region was introduced into the *Tet‐RAD18* strain on an integrative plasmid inserted at the *URA3* locus. All strains were grown at 30°C in YPD or synthetic complete (SC) medium supplemented with the appropriate amino acids. Cells were synchronized in G1 using 10 μg/ml α‐factor for 2 h. Auxin was used at 1 mM, doxycycline at 2 μg/ml.

### Analysis of DNA damage sensitivities and recovery of viability

Sensitivity to HU, MMS, and 4NQO was determined by spotting 10‐fold serial dilutions of exponentially growing cultures onto YPD plates containing the indicated concentrations of damaging agents. Plates were incubated at 30°C for 3 days before imaging. Recovery upon Rad18 induction was measured as described previously (Daigaku *et al*, 2010). Briefly, *Tet*‐*RAD18* cells were pre‐grown in YPD containing doxycycline, that is, under *RAD18*‐repressing conditions. They were then synchronized in G1, UV‐irradiated (20 J/m^2^) and released in the presence of doxycycline to maintain *RAD18* repression. At the indicated times, aliquots at appropriate dilutions were plated onto medium with or without doxycycline to either maintain repression (−Rad18) or induce expression of *RAD18* (+Rad18), respectively. After 2–3 days, colonies were counted and survival was determined relative to unirradiated cultures plated without doxycycline. Standard deviations (SD) were calculated from at least three independent experiments with three technical replicates each. For most recovery experiments involving degron‐tagged alleles, protein degradation was induced by adding auxin (Sigma) to the culture at the indicated times. Dilutions were then plated at the indicated times onto auxin‐containing medium (+Aux) to maintain protein degradation with (−Rad18) or without doxycycline (+Rad18). For the experiment shown in Fig 3C, Rad53 degradation was induced by adding auxin to the culture at the time of α‐factor addition. At the indicated times, dilutions were plated onto medium with or without auxin in the absence of doxycycline to induce *RAD18* expression (+Rad18).

### Pulsed‐field gel electrophoresis

As described above, G1‐synchronized cultures of the indicated strains were UV‐irradiated (20 J/m^2^) and released into S phase in the presence (−Rad18) or absence (+Rad18) of doxycycline, and samples were collected at the indicated times after release. Total DNA from 2 × 10^7^ cells was then extracted in low‐melting agarose plugs as described previously (Bianco *et al*, 2012), and chromosomes were resolved by PFGE in 1% agarose gels at 8°C using CHEF DR III (Bio‐Rad) under the following conditions: 175 V with 80 s pulse time for 12 h and 110 s pulse time for 12 h. Chromosomes V and IV were detected by Southern blots using specific probes.

### Detection of proteins

Total yeast protein extracts were prepared by trichloroacetic acid (TCA) precipitation as described (Morawska & Ulrich, 2013). Following SDS/PAGE and Western blotting, Rad53 phosphorylation was detected using monoclonal anti‐Rad53 antibody (kindly provided by S. Gasser). Monoclonal anti‐tubulin antibody YL1/2 (Sigma) was used to detect Rnr4 as described (Tsaponina *et al*, 2011). 9Myc‐ and 6HA‐tagged proteins were detected using monoclonal antibodies 9E10 (Enzo) and F7 (Santa Cruz), respectively. Monoclonal anti‐Pgk1 antibody 22C5D8 (Invitrogen) was used for loading controls. For visualization of Exo1^9myc^, Exo1^6HA^ and Pif1^6HA^ phosphorylation, proteins were resolved in Phos‐tag gels [8% polyacrylamide (29:1), containing 50 μM Phos‐tag reagent] prepared as described previously (Kinoshita *et al*, 2006).

### Fluorescence microscopy

In order to visualize Rad52^YFP^, cells carrying plasmid pWJ1213 were fixed with 2.5% formaldehyde in potassium phosphate at pH 6.4 for 10 min, washed twice with potassium phosphate at pH 6.6, and stored in potassium phosphate at pH 7.4. Cells were permeabilized with 80% ethanol for 10 min and resuspended in 0.5 μg/ml 4′,6‐diamidino‐2‐phenylindole (DAPI). At least 250 cells derived from three independent experiments were analyzed for each time point. Images were acquired with a 63× objective on a wide‐field fluorescence microscope (AF7000, Leica) equipped with an ORCA‐Flash 4.0 V2 digital CMOS camera (Hamamatsu) under the control of LAS AF software (Leica). Images were processed with ImageJ software (https://imagej.nih.gov/ij/). To analyze spindle formation, immunofluorescence of tubulin was performed as described (Matos *et al*, 2013), and images were acquired with a 63× objective on an Axio Imager (ZEISS) equipped with a Hamamatsu CCD Camera under the control of Volocity software.

### DNA fiber analysis

BrdU×7 cells (a kind gift from Philippe Pasero) were harvested in S phase after relevant treatments. Genomic DNA was prepared in agarose plugs as described previously (Bianco *et al*, 2012; Kaykov *et al*, 2016). Following proteinase K digestion and extensive washing in 1× TE with 100 mM NaCl, genomic DNA was extracted from plugs by melting in 50 mM MES, pH 6.0, with 100 mM NaCl. Combing was performed on vinylsilane‐coated coverslips with a molecular combing system (Genomic Vision). ssDNA was detected by immunostaining with mouse anti‐ssDNA antibody (MAB3034, Millipore) and Cy3‐labeled goat anti‐mouse secondary antibody (AB6946, Abcam). EdU tracts were labeled with Alexa Fluor 647 via Click reaction (Click‐iT Plus Imaging Kit, Invitrogen). DNA fibers were counterstained with YOYO‐1 and imaged with a Deltavision Elite System (GE Healthcare) equipped with a FITC/TRITC/Cy5 filter set. For analysis, binary images were obtained by applying absolute thresholds that were adjusted so as to eliminate non‐specific signals within unreplicated (YOYO‐1‐positive), undamaged DNA. Identical parameters were chosen for each set of samples processed together, but parameters were adjusted individually for different sets of experiments. The lengths of individual EdU tracts as well as the number of ssDNA tracts and the total length of ssDNA within each of these regions were determined using the ImageJ FIJI software. The same analysis was performed on EdU‐negative regions in the same set of fibers. The total percentage of ssDNA and the number of ssDNA tracts per unit length of DNA was then calculated and plotted for each tract. Statistical analysis was performed with GraphPad Prism v7.0.

## Author contributions

NG‐R, MM, YD, and HDU conceived the study; NG‐R, MM, RPW, and YD performed and analyzed experiments; NG‐R and HDU wrote the manuscript; and all authors discussed the data and commented on the manuscript.

## Conflict of interest

The authors declare that they have no conflict of interest.

## Supporting information



Appendix

Expanded View Figures PDF

Source Data for Expanded View

Review Process File

Source Data for Figure 4

Source Data for Figure 5

## References

[embj201798369-bib-0001] Aguilera A , Klein HL (1988) Genetic control of intrachromosomal recombination in *Saccharomyces cerevisiae*. I. Isolation and genetic characterization of hyper‐recombination mutations. Genetics 119: 779–790 3044923 10.1093/genetics/119.4.779 PMC1203464

[embj201798369-bib-0002] Alcasabas AA , Osborn AJ , Bachant J , Hu F , Werler PJ , Bousset K , Furuya K , Diffley JF , Carr AM , Elledge SJ (2001) Mrc1 transduces signals of DNA replication stress to activate Rad53. Nat Cell Biol 3: 958–965 11715016 10.1038/ncb1101-958

[embj201798369-bib-0003] Balint A , Kim T , Gallo D , Cussiol JR , Bastos de Oliveira FM , Yimit A , Ou J , Nakato R , Gurevich A , Shirahige K , Smolka MB , Zhang Z , Brown GW (2015) Assembly of Slx4 signaling complexes behind DNA replication forks. EMBO J 34: 2182–2197 26113155 10.15252/embj.201591190 PMC4557669

[embj201798369-bib-0004] Bianco JN , Poli J , Saksouk J , Bacal J , Silva MJ , Yoshida K , Lin YL , Tourriere H , Lengronne A , Pasero P (2012) Analysis of DNA replication profiles in budding yeast and mammalian cells using DNA combing. Methods 57: 149–157 22579803 10.1016/j.ymeth.2012.04.007

[embj201798369-bib-0005] Branzei D , Foiani M (2009) The checkpoint response to replication stress. DNA Rep 8: 1038–1046 10.1016/j.dnarep.2009.04.014 19482564

[embj201798369-bib-0006] de Bruin RA , Kalashnikova TI , Chahwan C , McDonald WH , Wohlschlegel J , Yates J III , Russell P , Wittenberg C (2006) Constraining G1‐specific transcription to late G1 phase: the MBF‐associated corepressor Nrm1 acts via negative feedback. Mol Cell 23: 483–496 16916637 10.1016/j.molcel.2006.06.025

[embj201798369-bib-0007] Byun TS , Pacek M , Yee MC , Walter JC , Cimprich KA (2005) Functional uncoupling of MCM helicase and DNA polymerase activities activates the ATR‐dependent checkpoint. Genes Dev 19: 1040–1052 15833913 10.1101/gad.1301205 PMC1091739

[embj201798369-bib-0008] Callegari AJ , Kelly TJ (2006) UV irradiation induces a postreplication DNA damage checkpoint. Proc Natl Acad Sci USA 103: 15877–15882 17043220 10.1073/pnas.0607343103 PMC1613229

[embj201798369-bib-0009] Callegari AJ , Clark E , Pneuman A , Kelly TJ (2010) Postreplication gaps at UV lesions are signals for checkpoint activation. Proc Natl Acad Sci USA 107: 8219–8224 20404181 10.1073/pnas.1003449107 PMC2889594

[embj201798369-bib-0010] Cotta‐Ramusino C , Fachinetti D , Lucca C , Doksani Y , Lopes M , Sogo J , Foiani M (2005) Exo1 processes stalled replication forks and counteracts fork reversal in checkpoint‐defective cells. Mol Cell 17: 153–159 15629726 10.1016/j.molcel.2004.11.032

[embj201798369-bib-0011] Crabbe L , Thomas A , Pantesco V , De Vos J , Pasero P , Lengronne A (2010) Analysis of replication profiles reveals key role of RFC‐Ctf18 in yeast replication stress response. Nat Struct Mol Biol 17: 1391–1397 20972444 10.1038/nsmb.1932

[embj201798369-bib-0012] Daigaku Y , Davies AA , Ulrich HD (2010) Ubiquitin‐dependent DNA damage bypass is separable from genome replication. Nature 465: 951–955 20453836 10.1038/nature09097 PMC2888004

[embj201798369-bib-0013] Dewar JM , Lydall D (2010) Pif1‐ and Exo1‐dependent nucleases coordinate checkpoint activation following telomere uncapping. EMBO J 29: 4020–4034 21045806 10.1038/emboj.2010.267 PMC3020640

[embj201798369-bib-0014] Doerfler L , Schmidt KH (2014) Exo1 phosphorylation status controls the hydroxyurea sensitivity of cells lacking the Pol32 subunit of DNA polymerases delta and zeta. DNA Rep 24: 26–36 10.1016/j.dnarep.2014.10.004 PMC4418189 25457771

[embj201798369-bib-0015] Elvers I , Johansson F , Groth P , Erixon K , Helleday T (2011) UV stalled replication forks restart by re‐priming in human fibroblasts. Nucleic Acids Res 39: 7049–7057 21646340 10.1093/nar/gkr420 PMC3167624

[embj201798369-bib-0016] Friedberg EC (2005) Suffering in silence: the tolerance of DNA damage. Nat Rev Mol Cell Biol 6: 943–953 16341080 10.1038/nrm1781

[embj201798369-bib-0017] Giannattasio M , Follonier C , Tourriere H , Puddu F , Lazzaro F , Pasero P , Lopes M , Plevani P , Muzi‐Falconi M (2010) Exo1 competes with repair synthesis, converts NER intermediates to long ssDNA gaps, and promotes checkpoint activation. Mol Cell 40: 50–62 20932474 10.1016/j.molcel.2010.09.004

[embj201798369-bib-0018] Giannattasio M , Zwicky K , Follonier C , Foiani M , Lopes M , Branzei D (2014) Visualization of recombination‐mediated damage bypass by template switching. Nat Struct Mol Biol 21: 884–892 25195051 10.1038/nsmb.2888 PMC4189914

[embj201798369-bib-0019] Gunjan A , Verreault A (2003) A Rad53 kinase‐dependent surveillance mechanism that regulates histone protein levels in *S. cerevisiae* . Cell 115: 537–549 14651846 10.1016/s0092-8674(03)00896-1

[embj201798369-bib-0020] Hang LE , Peng J , Tan W , Szakal B , Menolfi D , Sheng Z , Lobachev K , Branzei D , Feng W , Zhao X (2015) Rtt107 Is a multi‐functional scaffold supporting replication progression with partner SUMO and ubiquitin ligases. Mol Cell 60: 268–279 26439300 10.1016/j.molcel.2015.08.023 PMC4609303

[embj201798369-bib-0021] Hashimoto Y , Ray Chaudhuri A , Lopes M , Costanzo V (2010) Rad51 protects nascent DNA from Mre11‐dependent degradation and promotes continuous DNA synthesis. Nat Struct Mol Biol 17: 1305–1311 20935632 10.1038/nsmb.1927 PMC4306207

[embj201798369-bib-0022] Heller RC , Marians KJ (2006) Replication fork reactivation downstream of a blocked nascent leading strand. Nature 439: 557–562 16452972 10.1038/nature04329

[embj201798369-bib-0023] Hishida T , Kubota Y , Carr AM , Iwasaki H (2009) RAD6‐RAD18‐RAD5‐pathway‐dependent tolerance to chronic low‐dose ultraviolet light. Nature 457: 612–615 19079240 10.1038/nature07580

[embj201798369-bib-0024] Hoege C , Pfander B , Moldovan GL , Pyrowolakis G , Jentsch S (2002) RAD6‐dependent DNA repair is linked to modification of PCNA by ubiquitin and SUMO. Nature 419: 135–141 12226657 10.1038/nature00991

[embj201798369-bib-0025] Huang D , Piening BD , Paulovich AG (2013) The preference for error‐free or error‐prone postreplication repair in *Saccharomyces cerevisiae* exposed to low‐dose methyl methanesulfonate is cell cycle dependent. Mol Cell Biol 33: 1515–1527 23382077 10.1128/MCB.01392-12 PMC3624245

[embj201798369-bib-0026] Hung SH , Wong RP , Ulrich HD , Kao CF (2017) Monoubiquitylation of histone H2B contributes to the bypass of DNA damage during and after DNA replication. Proc Natl Acad Sci USA 114: E2205–E2214 28246327 10.1073/pnas.1612633114 PMC5358361

[embj201798369-bib-0027] Hustedt N , Gasser SM , Shimada K (2013) Replication checkpoint: tuning and coordination of replication forks in s phase. Genes 4: 388–434 24705211 10.3390/genes4030388 PMC3924824

[embj201798369-bib-0028] Ivessa AS , Lenzmeier BA , Bessler JB , Goudsouzian LK , Schnakenberg SL , Zakian VA (2003) The *Saccharomyces cerevisiae* helicase Rrm3p facilitates replication past nonhistone protein‐DNA complexes. Mol Cell 12: 1525–1536 14690605 10.1016/s1097-2765(03)00456-8

[embj201798369-bib-0029] Karras GI , Jentsch S (2010) The RAD6 DNA damage tolerance pathway operates uncoupled from the replication fork and is functional beyond S phase. Cell 141: 255–267 20403322 10.1016/j.cell.2010.02.028

[embj201798369-bib-0030] Karras GI , Fumasoni M , Sienski G , Vanoli F , Branzei D , Jentsch S (2013) Noncanonical role of the 9‐1‐1 clamp in the error‐free DNA damage tolerance pathway. Mol Cell 49: 536–546 23260657 10.1016/j.molcel.2012.11.016

[embj201798369-bib-0031] Kaykov A , Taillefumier T , Bensimon A , Nurse P (2016) Molecular combing of single DNA molecules on the 10 megabase scale. Sci Rep 6: 19636 26781994 10.1038/srep19636 PMC4726065

[embj201798369-bib-0032] Kinoshita E , Kinoshita‐Kikuta E , Takiyama K , Koike T (2006) Phosphate‐binding tag, a new tool to visualize phosphorylated proteins. Mol Cell Proteomics 5: 749–757 16340016 10.1074/mcp.T500024-MCP200

[embj201798369-bib-0033] Lopes M , Foiani M , Sogo JM (2006) Multiple mechanisms control chromosome integrity after replication fork uncoupling and restart at irreparable UV lesions. Mol Cell 21: 15–27 16387650 10.1016/j.molcel.2005.11.015

[embj201798369-bib-0034] Lopez‐Mosqueda J , Maas NL , Jonsson ZO , Defazio‐Eli LG , Wohlschlegel J , Toczyski DP (2010) Damage‐induced phosphorylation of Sld3 is important to block late origin firing. Nature 467: 479–483 20865002 10.1038/nature09377 PMC3393088

[embj201798369-bib-0035] Matos J , Blanco MG , West SC (2013) Cell‐cycle kinases coordinate the resolution of recombination intermediates with chromosome segregation. Cell Rep 4: 76–86 23810555 10.1016/j.celrep.2013.05.039

[embj201798369-bib-0036] Morawska M , Ulrich HD (2013) An expanded tool kit for the auxin‐inducible degron system in budding yeast. Yeast 30: 341–351 23836714 10.1002/yea.2967 PMC4171812

[embj201798369-bib-0037] Morin I , Ngo HP , Greenall A , Zubko MK , Morrice N , Lydall D (2008) Checkpoint‐dependent phosphorylation of Exo1 modulates the DNA damage response. EMBO J 27: 2400–2410 18756267 10.1038/emboj.2008.171 PMC2532783

[embj201798369-bib-0038] Nakada D , Hirano Y , Sugimoto K (2004) Requirement of the Mre11 complex and exonuclease 1 for activation of the Mec1 signaling pathway. Mol Cell Biol 24: 10016–10025 15509802 10.1128/MCB.24.22.10016-10025.2004 PMC525484

[embj201798369-bib-0039] Neecke H , Lucchini G , Longhese MP (1999) Cell cycle progression in the presence of irreparable DNA damage is controlled by a Mec1‐ and Rad53‐dependent checkpoint in budding yeast. EMBO J 18: 4485–4497 10449414 10.1093/emboj/18.16.4485 PMC1171523

[embj201798369-bib-0040] Nielsen I , Bentsen IB , Andersen AH , Gasser SM , Bjergbaek L (2013) A Rad53 independent function of Rad9 becomes crucial for genome maintenance in the absence of the Recq helicase Sgs1. PLoS One 8: e81015 24278365 10.1371/journal.pone.0081015 PMC3835667

[embj201798369-bib-0041] Nyberg KA , Michelson RJ , Putnam CW , Weinert TA (2002) Toward maintaining the genome: DNA damage and replication checkpoints. Annu Rev Genet 36: 617–656 12429704 10.1146/annurev.genet.36.060402.113540

[embj201798369-bib-0042] Ortiz‐Bazan MA , Gallo‐Fernandez M , Saugar I , Jimenez‐Martin A , Vazquez MV , Tercero JA (2014) Rad5 plays a major role in the cellular response to DNA damage during chromosome replication. Cell Rep 9: 460–468 25310987 10.1016/j.celrep.2014.09.005

[embj201798369-bib-0043] Paeschke K , Capra JA , Zakian VA (2011) DNA replication through G‐quadruplex motifs is promoted by the *Saccharomyces cerevisiae* Pif1 DNA helicase. Cell 145: 678–691 21620135 10.1016/j.cell.2011.04.015 PMC3129610

[embj201798369-bib-0044] Pages V , Santa Maria SR , Prakash L , Prakash S (2009) Role of DNA damage‐induced replication checkpoint in promoting lesion bypass by translesion synthesis in yeast. Genes Dev 23: 1438–1449 19528320 10.1101/gad.1793409 PMC2701570

[embj201798369-bib-0045] Pardo B , Crabbe L , Pasero P (2017) Signaling pathways of replication stress in yeast. FEMS Yeast Rev 17: fow101 10.1093/femsyr/fow101 27915243

[embj201798369-bib-0046] Rossi SE , Ajazi A , Carotenuto W , Foiani M , Giannattasio M (2015) Rad53‐mediated regulation of Rrm3 and Pif1 DNA helicases contributes to prevention of aberrant fork transitions under replication stress. Cell Rep 13: 80–92 26411679 10.1016/j.celrep.2015.08.073 PMC4597105

[embj201798369-bib-0047] Sabouri N , McDonald KR , Webb CJ , Cristea IM , Zakian VA (2012) DNA replication through hard‐to‐replicate sites, including both highly transcribed RNA Pol II and Pol III genes, requires the *S. pombe* Pfh1 helicase. Genes Dev 26: 581–593 22426534 10.1101/gad.184697.111 PMC3315119

[embj201798369-bib-0048] Sarkies P , Reams C , Simpson LJ , Sale JE (2010) Epigenetic instability due to defective replication of structured DNA. Mol Cell 40: 703–713 21145480 10.1016/j.molcel.2010.11.009 PMC3145961

[embj201798369-bib-0049] Schlacher K , Christ N , Siaud N , Egashira A , Wu H , Jasin M (2011) Double‐strand break repair‐independent role for BRCA2 in blocking stalled replication fork degradation by MRE11. Cell 145: 529–542 21565612 10.1016/j.cell.2011.03.041 PMC3261725

[embj201798369-bib-0050] Segurado M , Diffley JF (2008) Separate roles for the DNA damage checkpoint protein kinases in stabilizing DNA replication forks. Genes Dev 22: 1816–1827 18593882 10.1101/gad.477208 PMC2492668

[embj201798369-bib-0051] Shimada K , Pasero P , Gasser SM (2002) ORC and the intra‐S‐phase checkpoint: a threshold regulates Rad53p activation in S phase. Genes Dev 16: 3236–3252 12502744 10.1101/gad.239802 PMC187497

[embj201798369-bib-0052] Smolka MB , Albuquerque CP , Chen SH , Zhou H (2007) Proteome‐wide identification of *in vivo* targets of DNA damage checkpoint kinases. Proc Natl Acad Sci USA 104: 10364–10369 17563356 10.1073/pnas.0701622104 PMC1965519

[embj201798369-bib-0053] Tercero JA , Longhese MP , Diffley JF (2003) A central role for DNA replication forks in checkpoint activation and response. Mol Cell 11: 1323–1336 12769855 10.1016/s1097-2765(03)00169-2

[embj201798369-bib-0054] Travesa A , Kuo D , de Bruin RA , Kalashnikova TI , Guaderrama M , Thai K , Aslanian A , Smolka MB , Yates JR III , Ideker T , Wittenberg C (2012) DNA replication stress differentially regulates G1/S genes via Rad53‐dependent inactivation of Nrm1. EMBO J 31: 1811–1822 22333915 10.1038/emboj.2012.28 PMC3321207

[embj201798369-bib-0055] Tsang E , Miyabe I , Iraqui I , Zheng J , Lambert SA , Carr AM (2014) The extent of error‐prone replication restart by homologous recombination is controlled by Exo1 and checkpoint proteins. J Cell Sci 127: 2983–2994 24806966 10.1242/jcs.152678 PMC4075360

[embj201798369-bib-0056] Tsaponina O , Barsoum E , Astrom SU , Chabes A (2011) Ixr1 is required for the expression of the ribonucleotide reductase Rnr1 and maintenance of dNTP pools. PLoS Genet 7: e1002061 21573136 10.1371/journal.pgen.1002061 PMC3088718

[embj201798369-bib-0057] Ulrich HD (2009) Regulating post‐translational modifications of the eukaryotic replication clamp PCNA. DNA Rep 8: 461–469 10.1016/j.dnarep.2009.01.006 19217833

[embj201798369-bib-0058] Vanoli F , Fumasoni M , Szakal B , Maloisel L , Branzei D (2010) Replication and recombination factors contributing to recombination‐dependent bypass of DNA lesions by template switch. PLoS Genet 6: e1001205 21085632 10.1371/journal.pgen.1001205 PMC2978687

[embj201798369-bib-0059] Walter J , Newport J (2000) Initiation of eukaryotic DNA replication: origin unwinding and sequential chromatin association of Cdc45, RPA, and DNA polymerase alpha. Mol Cell 5: 617–627 10882098 10.1016/s1097-2765(00)80241-5

[embj201798369-bib-0060] Waters LS , Walker GC (2006) The critical mutagenic translesion DNA polymerase Rev1 is highly expressed during G(2)/M phase rather than S phase. Proc Natl Acad Sci USA 103: 8971–8976 16751278 10.1073/pnas.0510167103 PMC1482550

[embj201798369-bib-0061] Wysocki R , Javaheri A , Allard S , Sha F , Cote J , Kron SJ (2005) Role of Dot1‐dependent histone H3 methylation in G1 and S phase DNA damage checkpoint functions of Rad9. Mol Cell Biol 25: 8430–8443 16166626 10.1128/MCB.25.19.8430-8443.2005 PMC1265753

[embj201798369-bib-0062] Zegerman P , Diffley JF (2010) Checkpoint‐dependent inhibition of DNA replication initiation by Sld3 and Dbf4 phosphorylation. Nature 467: 474–478 20835227 10.1038/nature09373 PMC2948544

