# Supplementary material for: Spatial separation between replisome‐ and template‐induced replication stress signaling
Source: EMBO J. 2018 Mar 26;37(9):e98369. doi: 10.15252/embj.201798369 (PMC5920239; doi:10.15252/embj.201798369)
Supplement: Supplementary file 1 — Appendix [file EMBJ-37-e98369-s001.pdf]

APPENDIX

Spatial separation between replisome- and template-induced replication stress signaling

Néstor García-Rodríguez[1], Magdalena Morawska[1,2#], Ronald P. Wong[1], Yasukazu Daigaku[2$] and Helle D. Ulrich[1*]

Pg. 2: Appendix Table S1

Pg. 5: Appendix Figure Legends S1-S3

Pg. 6: Appendix References

Pg. 7-9: Appendix Figures S1-S3

Appendix Table S1: Yeast strains used in this study

Strain	Genotype	Source
WT (DF5)	*Mat a, his3-Δ200, leu2-3,112, lys2-801, trp1-1, ura3-52*	(Finley et al, 1987)
rad18Δ	DF5 *rad18Δ::TRP1*	(Stelter & Ulrich, 2003)
rad53Δ sml1Δ	DF5 *rad53Δ::HIS3MX, sml1Δ::hisG-URA3-hisG*	This study
mec1Δ sml1Δ	DF5 *mec1Δ::KanMX, sml1Δ::hisG-URA3-hisG, bar1Δ::HIS3MX*	This study
mrc1Δ	DF5 *mrc1Δ::natNT2*	This study
rad9Δ	DF5 *rad9Δ::URA3*	This study
mrc1Δ rad9Δ sml1Δ	DF5 *mrc1Δ::natNT2, rad9Δ::HIS3MX, sml1Δ::hisG-URA3-hisG,*	This study
ddc1Δ	DF5 *ddc1Δ::HIS3MX*	This study
rad18Δ rad53Δ sml1Δ	DF5 *rad18Δ::TRP1, rad53Δ::KanMX, sml1 Δ::hisG-URA3-hisG*	This study
rad18Δ mec1Δ sml1Δ	DF5 *rad18Δ::TRP1, mec1Δ::KanMX, sml1Δ::hisG-URA3-hisG*	This study
rad18Δ mrc1Δ	DF5 *rad18Δ::TRP1, mrcΔ1::natNT2*	This study
rad18Δ rad9Δ	DF5 *rad18Δ::TRP1, rad9Δ::hphNT1*	This study
rad18Δ mrc1Δ rad9Δ sml1Δ	DF5 *rad18Δ::TRP1, mrc1Δ::natNT2, rad9Δ::HIS3MX, sml1Δ::hisG-URA3-hisG*	This study
Tet-RAD18	DF5 *KanMX::TetO$_7$-RAD18, LEU2::TetR'-SSN6, TRP1::BrdU-inc*	This study
Tet-RAD18 rad53Δ sml1Δ	*Tet-RAD18, rad53Δ::HIS3MX, sml1Δ::hisG-URA3-hisG*	This study
Tet-RAD18 mec1Δ sml1Δ	*Tet-RAD18, mec1Δ::HIS3MX, sml1Δ::hisG-URA3-hisG*	This study
Tet-RAD18 mrc1Δ	*Tet-RAD18, mrc1Δ::natNT2*	This study
Tet-RAD18 rad9Δ	*Tet-RAD18, rad9Δ::HIS3MX*	This study
Tet-RAD18 mrc1Δ rad9Δ sml1Δ	*Tet-RAD18, mrc1Δ::natNT2, rad9Δ::HIS3MX, sml1Δ::hisG-URA3-hisG*	This study
Tet-RAD18 ^{His}POL30	*Tet-RAD18, URA3::YIp211-^{His6}POL30*	This study
Tet-RAD18 rad53Δ sml1Δ ^{His}POL30	*Tet-RAD18, rad53 Δ::HIS3MX, sml1Δ::hisG, URA3::YIp211-^{His6}POL30*	This study
Tet-RAD18 rad53-K227A sml1 Δ	*Tet-RAD18, sml1Δ::hisG, rad53-K227A*	This study
Tet-RAD18 rad53$^{AID*-9myc}$ sml1Δ	*Tet-RAD18, URA3:: ADH1-AtTIR1^{9myc}, sml1Δ::hisG-URA3-hisG, RAD53$^{AID*-9myc}$::hphNT1*	(Morawska & Ulrich, 2013)
Tet-RAD18 dun1Δ	*Tet-RAD18, dun1Δ::HIS3MX*	This study
Tet-RAD18 dun1Δ sml1Δ	*Tet-RAD18, dun1Δ::HIS3MX, sml1Δ::hisG-URA3-hisG*	This study
Tet-RAD18 dun11Δ sml1Δ crt1Δ	*Tet-RAD18, dun1Δ::HIS3MX, sml1Δ::hisG-URA3-hisG, crt1Δ::natNT2*	This study

Tet-RAD18 rad53Δ sml1Δ crt1Δ	*Tet-RAD18, rad53Δ::HIS3MX, sml1Δ::hisG-URA3-hisG, crt1Δ::natNT2*	This study
Tet-RAD18 dbf4-4A	*Tet-RAD18, HIS3::*P_{DBF4}*-Dbf4-4A, dbf4Δ::URA3*	This study
Tet-RAD18 sld3-A	*Tet-RAD18, sld3-38A*$^{-10his-13myc}$*::KanMX*	This study
Tet-RAD18 dbf4-4A sld3-A	*Tet-RAD18, HIS3::*P_{DBF4}*-Dbf4-4A, dbf4Δ::URA3, sld3-38A*$^{-10his-13myc}$*::KanMX*	This study
Tet-RAD18 nrm1Δ	*Tet-RAD18, nrm1Δ::hphNT1*	This study
Tet-RAD18 rad53Δ sml1Δ nrm1Δ	*Tet-RAD18, rad53Δ::HIS3MX, sml1Δ::hisG-URA3-hisG, nrm1Δ::hphNT1*	This study
Tet-RAD18 hht2-hhf2Δ	*Tet-RAD18, hht2-hhf2Δ::hphNT1*	This study
Tet-RAD18 rad53Δ sml1Δ hht2-hhf2Δ	*Tet-RAD18, rad53Δ::HIS3MX, sml1Δ::hisG-URA3-hisG, hht2-hhf2Δ::hphNT1*	This study
Tet-RAD18 mre11Δ	*Tet-RAD18, mre11Δ::hphNT1*	This study
Tet-RAD18 rad53Δ sml1Δ mre11Δ	*Tet-RAD18, rad53Δ::HIS3MX, sml1Δ::hisG-URA3-hisG, mre11Δ::hphNT1*	This study
Tet-RAD18 rad55Δ	*Tet-RAD18, rad55Δ::hphNT1*	This study
Tet-RAD18 rad53Δ sml1Δ rad55Δ	*Tet-RAD18, rad53Δ::HIS3MX, sml1Δ::hisG-URA3-hisG, rad55Δ::hphNT1*	This study
Tet-RAD18 mms4Δ	*Tet-RAD18, mms4Δ::hphNT1*	This study
Tet-RAD18 rad53Δ sml1Δ mms4Δ	*Tet-RAD18, rad53Δ::HIS3MX, sml1Δ::hisG-URA3-hisG, mms4Δ::hphNT1*	This study
Tet-RAD18 slx4Δ	*Tet-RAD18, slx4Δ::hphNT1*	This study
Tet-RAD18 rad53Δ sml1Δ slx4Δ	*Tet-RAD18, rad53Δ::HIS3MX, sml1Δ::hisG-URA3-hisG, slx4Δ::hphNT1*	This study
Tet-RAD18 yen1Δ	*Tet-RAD18, yen1Δ::hphNT1*	This study
Tet-RAD18 rad53Δ sml1Δ yen1Δ	*Tet-RAD18, rad53Δ::HIS3MX, sml1Δ::hisG-URA3-hisG, yen1Δ::hphNT1*	This study
Tet-RAD18 sgs1Δ	*Tet-RAD18, sgs1Δ::hphNT1*	This study
Tet-RAD18 rad53Δ sml1Δ sgs1Δ	*Tet-RAD18, rad53Δ::HIS3MX, sml1Δ::hisG-URA3-hisG, sgs1Δ::hphNT1*	This study
Tet-RAD18 srs2Δ	*Tet-RAD18, srs2Δ::natNT2*	This study
Tet-RAD18 rad53Δ sml1Δ srs2Δ	*Tet-RAD18, rad53Δ::HIS3MX, sml1Δ::hisG-URA3-hisG, srs2Δ::natNT2*	This study
Tet-RAD18 exo1Δ	*Tet-RAD18, exo1Δ::hphNT1*	This study
Tet-RAD18 rad53Δ sml1Δ exo1Δ	*Tet-RAD18, rad53Δ::HIS3MX, sml1Δ::hisG-URA3-hisG, exo11Δ::hphNT1*	This study
Tet-RAD18 EXO1^{9myc}	*Tet-RAD18, EXO1*^{9myc}*::hphNT1*	This study
Tet-RAD18 rad53Δ sml1Δ EXO1^{9myc}	*Tet-RAD18, rad53Δ::HIS3MX, sml1Δ::hisG-URA3-hisG, EXO1*^{9myc}*::hphNT1*	This study
Tet-RAD18 rad9Δ EXO1^{9myc}	*Tet-RAD18, rad9Δ::HIS3MX, EXO1*^{9myc}*::hphNT1*	This study

Tet-RAD18 mrc1Δ EXO1^{9myc}	*Tet-RAD18, mrc1Δ::natNT2, EXO1^{9myc}::hphNT1*	This study
Tet-RAD18 rad9Δ mrc1Δ sml1Δ EXO1^{9myc}	*Tet-RAD18, mrc1Δ::natNT2, rad9Δ::HIS3MX, sml1Δ::hisG-URA3-hisG, EXO1^{9myc}::hphNT1*	This study
Tet-RAD18 exo1-SA	*Tet-RAD18, exo1-SA::HIS3MX*	This study
Tet-RAD18 exo1-SA-ND	*Tet-RAD18, exo1-SA-ND::HIS3MX*	This study
Tet-RAD18 EXO1 + exo1-SA	*Tet-RAD18, URA3::YIp211-exo1-SA*	This study
Tet-RAD18 exo1-SA^{9myc}	*Tet-RAD18, exo1-SA^{9myc}::hphNT1*	This study
Tet-RAD18 rad53$^{AID*-9myc}$ sml1Δ EXO1^{6HA}	*Tet-RAD18, URA3:: ADH1-AtTIR1^{9myc}, sml1Δ::hisG-URA3-hisG, RAD53$^{AID*-9myc}$::hphNT1, EXO1^{6HA}::natNT2*	This study
EXO1^{9myc}	DF5 *EXO1^{9myc}::hphNT1*	This study
Tet-RAD18 rad53Δ sml1Δ rrm3Δ	*Tet-RAD18, rad53Δ::HIS3MX, sml1Δ::hisG-URA3-hisG, rrm3Δ::HIS3MX*	This study
Tet-RAD18 rad53Δ sml1Δ pif1Δ	*Tet-RAD18, rad53Δ::HIS3MX, sml1Δ::hisG-URA3-hisG, pif1Δ::hphNT1*	This study
Tet-RAD18 rad53Δ sml1Δ exo1Δ rrm3Δ	*Tet-RAD18, rad53Δ::HIS3MX, sml1Δ::hisG-URA3-hisG, exo1Δ::natNT2, rrm3Δ::HIS3MX*	This study
Tet-RAD18 rad53Δ sml1Δ exo1Δ pif1Δ	*Tet-RAD18, rad53Δ::HIS3MX, sml1Δ::hisG-URA3-hisG, exo1Δ::natNT2, pif1Δ::hphNT1*	This study
Tet-RAD18 pif1Δ	*Tet-RAD18, pif1Δ::hphNT1*	This study
Tet-RAD18 exo1Δ pif1Δ	*Tet-RAD18, exo1Δ::natNT2, pif1Δ::hphNT1*	This study
Tet-RAD18 PIF1^{6HA}	*Tet-RAD18, PIF1^{6HA}::natNT2*	This study
Tet-RAD18 rad53Δ sml1Δ PIF1^{6HA}	*Tet-RAD18, rad53Δ::HIS3MX, sml1Δ::hisG-URA3-hisG, PIF1^{6HA}::natNT2*	This study
PIF1^{6HA}	DF5 *PIF1^{6HA}::natNT2*	This study
Tet-RAD18 exo1Δ mre11Δ	*Tet-RAD18, exo1Δ::hphNT1, mre11Δ::natNT2*	This study
Tet-RAD18 pol1$^{AID*-9myc}$	*Tet-RAD18, URA3:: ADH1-AtTIR1^{9myc}, POL1$^{AID*-9myc}$::hphNT1*	(Morawska & Ulrich, 2013)
Tet-RAD18 pol1$^{AID*-9myc}$ exo1Δ	*Tet-RAD18, URA3:: ADH1-AtTIR1^{9myc}, POL1$^{AID*-9myc}$::hphNT1, exo1Δ::natNT2*	This study
BrdU Inc x7 (in W303 a)	*ade2-1, trp1-1, can1-100, leu2-3,112, his3-11,15, ura3, GAL, psi+, RAD5, URA::GPD-TK×7, phENT1-LEU2*	(Bianco et al, 2012)
BrdU Inc x7 exo1Δ	*ade2-1, trp1-1, can1-100, leu2-3,112, his3-11,15, ura3, GAL, psi+, RAD5, URA::GPD-TK×7, phENT1-LEU2 exo1Δ::hphNT1*	This study

APPENDIX FIGURE LEGENDS

Appendix Figure S1 - Rad53 is required during the S phase that precedes DNA damage bypass.

A Rad53$^{AID*-9myc}$ is rapidly degraded upon addition of auxin. Pgk1 was used as loading control.

B Recovery assay upon *RAD18* induction comparing *WT* and *rad53*$^{AID*-9myc}$, performed as described in Fig. 3A, but without auxin-induced degradation of Rad53 during synchronization. Error bars indicate SD derived from at least three independent experiments.

C Recovery assay upon *RAD18* induction comparing *WT* and *rad53*$^{AID*-9myc}$, performed as described in Fig. 3B, but without auxin-induced degradation of Rad53 at 4h. Error bars indicate SD derived from at least three independent experiments.

D Western blot analysis showing efficient degradation of Rad53$^{AID*-9myc}$ upon addition of auxin 4 h after release into S phase.

E Analysis of Rad53$^{AID*-9myc}$ degradation (during synchronization) and re-expression (upon auxin removal after 0 or 2 h) as described in Fig. 3C. Left: experimental scheme; right: western blot analysis of Rad53$^{AID*-9myc}$ and Rnr4 protein levels, indicative of checkpoint activation. Pgk1 served as loading control.

Appendix Figure S2 - Exo1 is required for robust checkpoint activation in response to MMS-induced replication stress.

A Rad53 phosphorylation in the indicated strains, synchronized in G1, treated with MMS (0.04%) for 30 min and released into S phase. Decline of Sic1 levels is shown as a marker for G1/S transition. Pgk1 served as loading control. Cell cycle profiles are shown below the blots.

B Percentage of budded cells of the indicated strains treated as described in panel (A) (n=100).

Appendix Figure S3 – ssDNA arises within replication tracts on damaged DNA.

A-C ssDNA does not accumulate within replication tracts in response to HU treatment. DNA combing was performed on total genomic DNA isolated from control and HU-treated cells as described for Fig. 6A, but HU treatment was with 60 mM HU, and cells were harvested after 30 min in EdU for control and after 60 min for HU treatment.

A Representative images showing YOYO-1 staining for total DNA (blue), Alexa Fluor 647 labelling of EdU tracts (red), and antibody staining of ssDNA (green). Scale bar = 20 kbp.

B Quantification of replication tract lengths. Number of EdU tracts analyzed: Control: 59; HU: 62. Significance was calculated by the Mann-Whitney test (ns: not significant). Black bar = mean.

C Quantification of ssDNA within the same set of EdU tracts analyzed in panel B, determined for individual tracts by measuring total tract length and total length of ssDNA within that tract.

D-E EdU-stained regions on DNA fibers arise in an S phase-dependent manner and thus represent replication tracts. G1-arrested cells treated with 0.02% MMS for 30 mins were either released into S phase or held in G1 phase for 30 mins in the presence of EdU. Lengths of EdU tracts were measured in 100 individual DNA fibers.

D Quantification of relative EdU tract length based on individual fibers, determined by dividing the total length of all EdU tracts within individual fibers by the total length of that fiber. Significance was calculated by the Mann-Whitney test (****: $p<0.001$). Black bar = mean.

E Quantification of relative EdU tract length in bulk, determined on the same set of fibers as in D by dividing the total length of all EdU tracts by the total length of all fibers.

APPENDIX REFERENCES

Bianco JN, Poli J, Saksouk J, Bacal J, Silva MJ, Yoshida K, Lin YL, Tourriere H, Lengronne A, Pasero P (2012) Analysis of DNA replication profiles in budding yeast and mammalian cells using DNA combing. *Methods* **57:** 149-157

Finley D, Ozkaynak E, Varshavsky A (1987) The yeast polyubiquitin gene is essential for resistance to high temperatures, starvation, and other stresses. *Cell* **48:** 1035-1046

Morawska M, Ulrich HD (2013) An expanded tool kit for the auxin-inducible degron system in budding yeast. *Yeast* **30:** 341-351

Stelter P, Ulrich HD (2003) Control of spontaneous and damage-induced mutagenesis by SUMO and ubiquitin conjugation. *Nature* **425:** 188-191

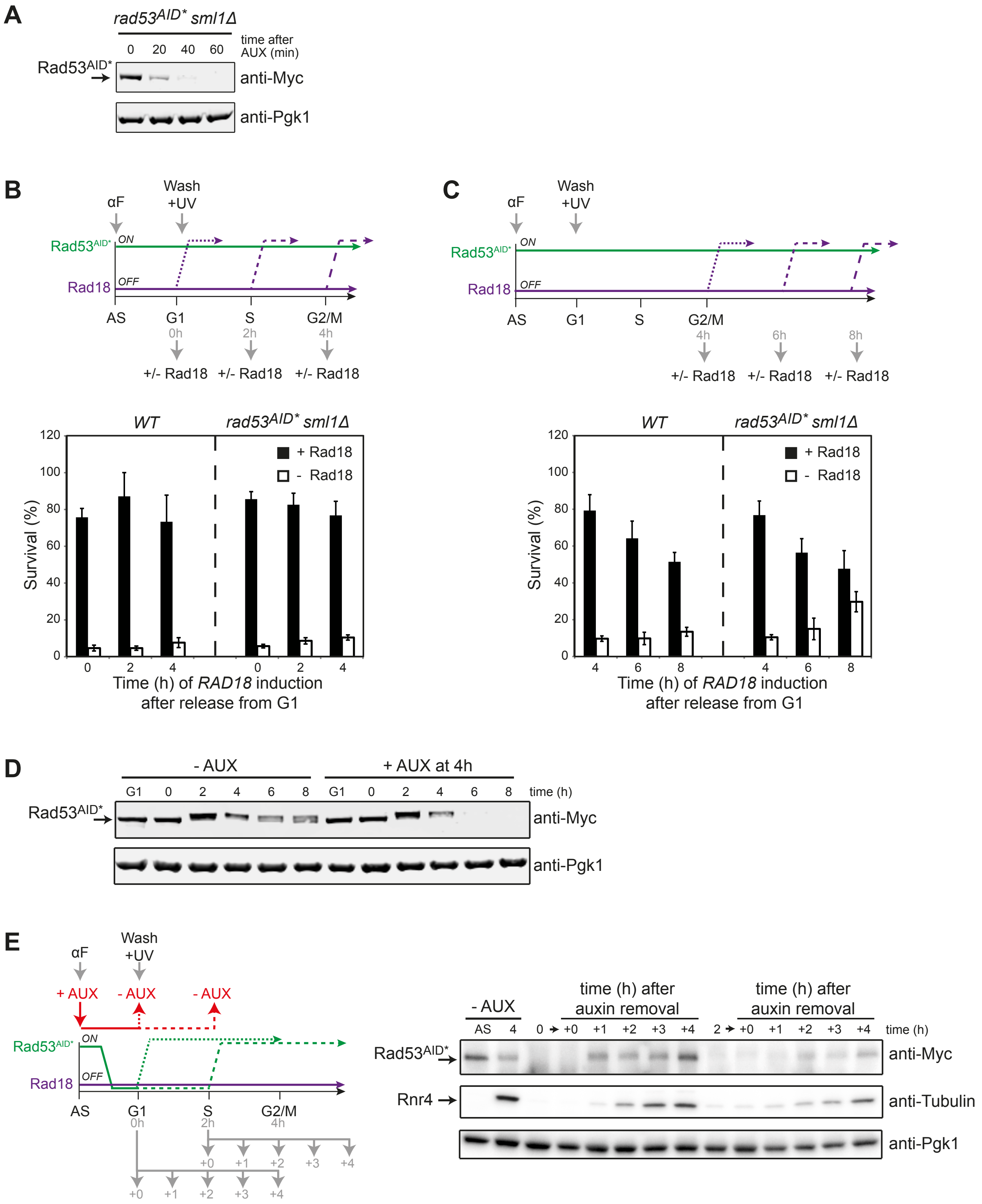
A
rad53AID* sml1Δ
0 20 40 60
time after AUX (min)
Rad53AID*
anti-Myc
anti-Pgk1
B
αF
Wash +UV
Rad53AID*
ON
Rad18
OFF
AS
G1
S
G2/M
0h
2h
4h
+/- Rad18
+/- Rad18
+/- Rad18
WT
rad53AID* sml1Δ
+ Rad18
- Rad18
Survival (%)
120
100
80
60
40
20
0
0 2 4 0 2 4
Time (h) of RAD18 induction after release from G1
C
αF
Wash +UV
Rad53AID*
ON
Rad18
OFF
AS
G1
S
G2/M
4h
6h
8h
+/- Rad18
+/- Rad18
+/- Rad18
WT
rad53AID* sml1Δ
+ Rad18
- Rad18
Survival (%)
120
100
80
60
40
20
0
4 6 8 4 6 8
Time (h) of RAD18 induction after release from G1
D
- AUX
+ AUX at 4h
G1 0 2 4 6 8 G1 0 2 4 6 8
time (h)
Rad53AID*
anti-Myc
anti-Pgk1
E
αF
Wash +UV
+ AUX
- AUX
- AUX
Rad53AID*
ON
Rad18
OFF
AS
G1
0h
S
2h
G2/M
4h
+0 +1 +2 +3 +4
+0 +1 +2 +3 +4
- AUX
AS 4
time (h) after auxin removal
0 → +0 +1 +2 +3 +4
time (h) after auxin removal
2 → +0 +1 +2 +3 +4
time (h)
Rad53AID*
anti-Myc
Rnr4
anti-Tubulin
anti-Pgk1

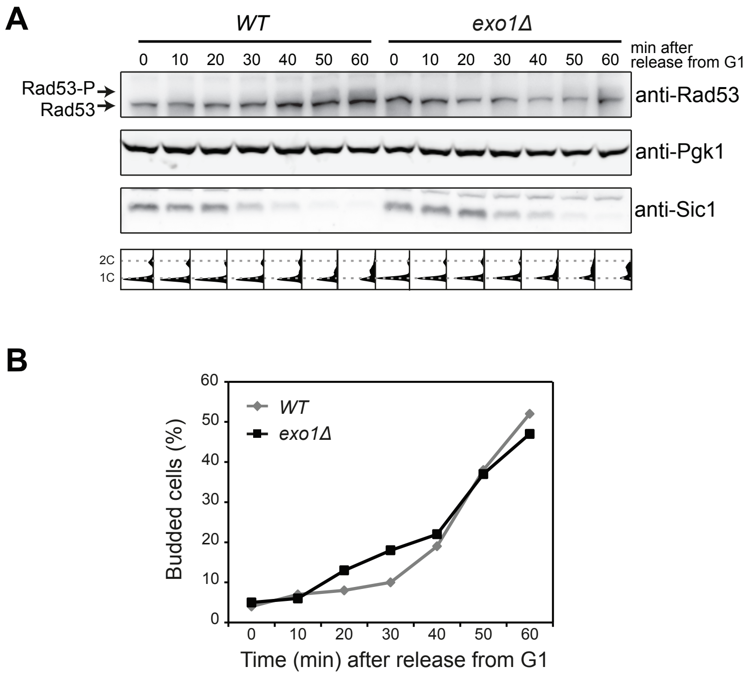
A
WT
exo1Δ
min after release from G1
0 10 20 30 40 50 60 0 10 20 30 40 50 60
Rad53-P
Rad53
anti-Rad53
anti-Pgk1
anti-Sic1
2C
1C
B
Budded cells (%)
WT
exo1Δ
Time (min) after release from G1
0 10 20 30 40 50 60

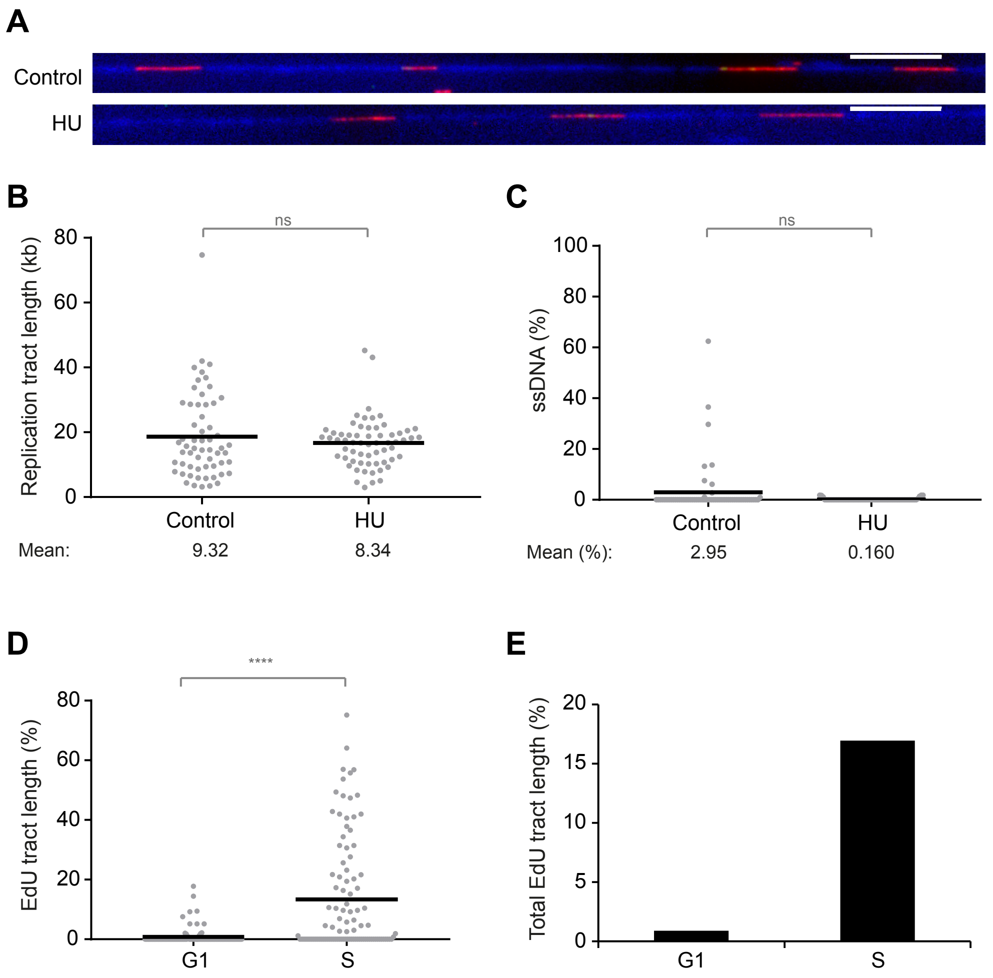
A
Control
HU
B
ns
Replication tract length (kb)
80
60
40
20
0
Control
HU
Mean:
9.32
8.34
C
ns
ssDNA (%)
100
80
60
40
20
0
Control
HU
Mean (%):
2.95
0.160
D
****
EdU tract length (%)
80
60
40
20
0
G1
S
E
Total EdU tract length (%)
20
15
10
5
0
G1
S
