# Supplementary material for: Spatial separation between replisome‐ and template‐induced replication stress signaling
Source: EMBO J. 2018 Mar 26;37(9):e98369. doi: 10.15252/embj.201798369 (PMC5920239; doi:10.15252/embj.201798369)
Supplement: Supplementary file 2 — Expanded View Figures PDF [file EMBJ-37-e98369-s002.pdf]

Expanded View Figures

Figure EV1. Contribution of checkpoint factors to DNA damage bypass.

A DNA damage sensitivities of yeast strains carrying the indicated gene deletions, determined by growth assays.
B Re-expression of Rad18 (top) and ^{His}PCNA ubiquitylation (bottom) after removing doxycycline in UV-irradiated cells of the indicated strains. Ubiquitylation of His_6-tagged PCNA was detected as described previously (Daigaku *et al*, 2010).
C Experimental scheme for measuring viability after UV irradiation (20 J/m^2) at the indicated times after release into S phase, performed as described in Fig 1B, but under conditions of continuous *Tet-RAD18* expression (AS: asynchronous; αF: alpha-factor).
D Survival of the indicated strains, relative to unirradiated controls. Survival above 100% reflects cell division within 4 h.
E Recovery of viability is abolished in a catalytically inactive *rad53* mutant.
F Magnification of selected panels from Fig 1D.
G Rad53 phosphorylation in the specified strains upon release into S phase after UV irradiation in the absence of Rad18, monitored by Western blotting.

Data information: (D–F) Error bars indicate SD derived from at least three independent experiments.
Source data are available online for this figure.

© 2018 The Authors

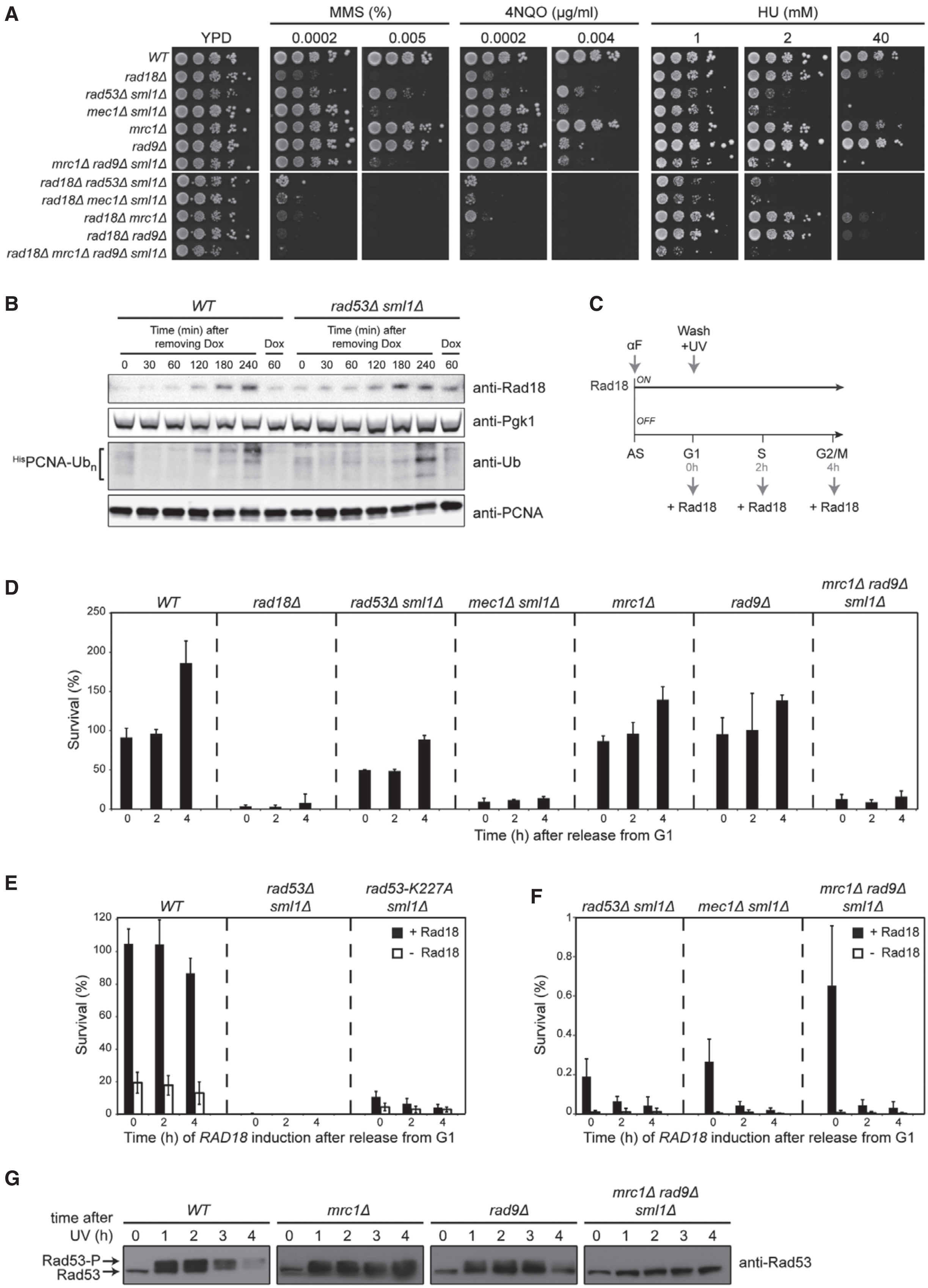
A
MMS (%)
4NQO (µg/ml)
HU (mM)
YPD
0.0002
0.005
0.0002
0.004
1
2
40
WT
rad18Δ
rad53Δ sml1Δ
mec1Δ sml1Δ
mrc1Δ
rad9Δ
mrc1Δ rad9Δ sml1Δ
rad18Δ rad53Δ sml1Δ
rad18Δ mec1Δ sml1Δ
rad18Δ mrc1Δ
rad18Δ rad9Δ
rad18Δ mrc1Δ rad9Δ sml1Δ
B
WT
rad53Δ sml1Δ
Time (min) after removing Dox
Dox
0 30 60 120 180 240 60
anti-Rad18
anti-Pgk1
HisPCNA-Ubn
anti-Ub
anti-PCNA
C
αF
Wash +UV
Rad18
ON
OFF
AS
G1 0h
S 2h
G2/M 4h
+ Rad18
D
WT
rad18Δ
rad53Δ sml1Δ
mec1Δ sml1Δ
mrc1Δ
rad9Δ
mrc1Δ rad9Δ sml1Δ
Survival (%)
Time (h) after release from G1
E
WT
rad53Δ sml1Δ
rad53-K227A sml1Δ
+ Rad18
- Rad18
Survival (%)
Time (h) of RAD18 induction after release from G1
F
rad53Δ sml1Δ
mec1Δ sml1Δ
mrc1Δ rad9Δ sml1Δ
+ Rad18
- Rad18
Survival (%)
Time (h) of RAD18 induction after release from G1
G
time after UV (h)
WT
mrc1Δ
rad9Δ
mrc1Δ rad9Δ sml1Δ
0 1 2 3 4
Rad53-P
Rad53
anti-Rad53

Figure EV1.

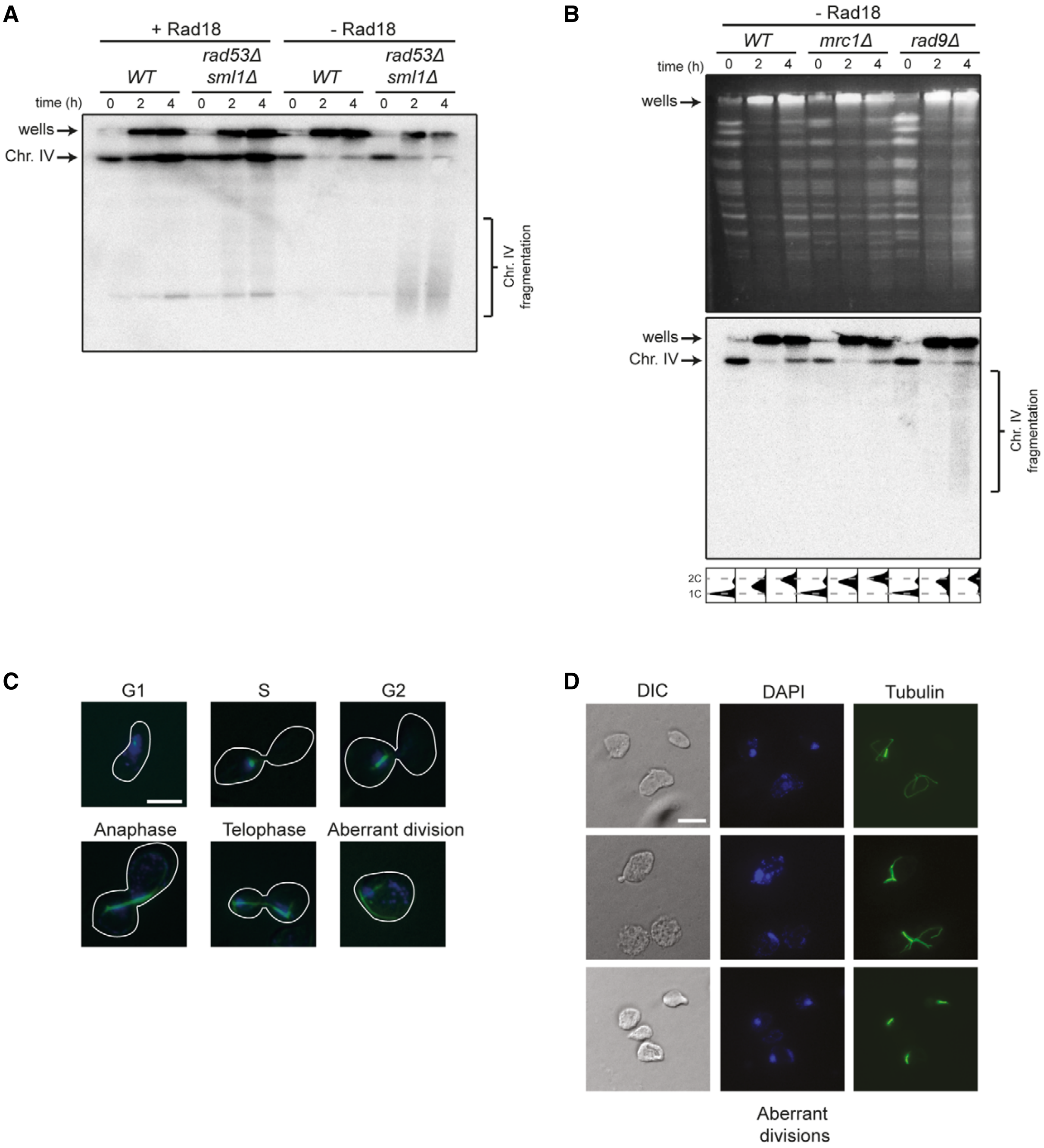

Figure EV2. Consequences of delaying damage bypass in checkpoint mutants.

A Pulsed-field gel electrophoresis and Southern blotting analysis of chromosome IV in *WT* and *rad53Δ* released into S phase after UV irradiation in the presence or absence of Rad18. Replication intermediates accumulate in the wells.

B Pulsed-field gel electrophoresis analyzed by ethidium bromide staining (top) and Southern blotting (middle) for chromosome IV in *WT*, *mrc1Δ*, and *rad9Δ* in the absence of Rad18, treated as above. Cell cycle profiles are shown at the bottom.

C Classification of cells into cell cycle stages according to spindle morphology (blue: DAPI: green: tubulin). Scale bar = 5 μm.

D Examples of cells undergoing aberrant divisions.

© 2018 The Authors

Figure EV3. Analysis of downstream targets of Rad53.
Recovery assays were performed according to the scheme in Fig 1B, by releasing UV-irradiated G1 cells into S phase in the absence of Rad18 and inducing *Tet-RAD18* expression at the indicated times.

A In response to replication stress, Rad53 upregulates dNTP production through phosphorylation of Dun1, which in turn targets the transcriptional repressor Crt1 and the protein inhibitors Sml1 and Dif1, thereby relieving ribonucleotide reductase (RNR) inhibition and boosting dNTP levels (Hustedt *et al*, 2013) (left). Deletion of *DUN1* reduces the capacity of cells to recover, and this effect is suppressed partially by deletion of *SML1* and almost completely by a concomitant deletion of *SML1* and *CRT1* (middle). Hence, upregulation of dNTP levels is required for efficient damage bypass. However, *sml1Δ* and *crt1Δ* do not compensate for loss of *RAD53* (right). Therefore, upregulation of dNTP levels is required, but not sufficient to maintain bypass competence.

B In response to replication stress, Rad53 inhibits late origin firing through phosphorylation of Dbf4 and Sld3 (Lopez-Mosqueda *et al*, 2010; Zegerman & Diffley, 2010) (left). Cells expressing non-phosphorylatable alleles of *DBF4* and *SLD3* (*dbf4-4A sld3-A*) (Zegerman & Diffley, 2010) recover virtually as efficiently as *WT* cells (middle), even though they progress through S phase as rapidly as the *rad53Δ* mutant (right). Hence, accelerated progression through S phase or firing of late origins does not preclude efficient postreplicative gap filling.

C Delay of mitosis by nocodazole treatment is insufficient to restore viability in *rad53Δ*. Middle: recovery assays performed with or without addition of nocodazole (added twice: 15 μg/ml at 0 h and 10 μg/ml at 2 h). Right: magnification of the graphs showing *rad53Δ*.

D Rad53 is responsible for induction of a set of MBF-regulated genes during S phase in response to DNA damage, mediated via inactivation of the transcriptional co-repressor Nrm1 (left) (Travesa *et al*, 2012). Hence, *NRM1* deletion suppresses *rad53Δ*-associated lethality (de Bruin *et al*, 2006). However, *nrm1Δ* does not restore Rad18-dependent recovery of viability in *rad53Δ* (right), indicating that Nrm1 is not a relevant target of Rad53 in this context.

E Rad53 is required for degradation of excess histones upon DNA damage (Gunjan & Verreault, 2003) (left). Reduction in histone gene dosage therefore suppresses HU and MMS sensitivities of *rad53Δ* (Gunjan & Verreault, 2003). However, reducing histone dosage by deletion of *HHT2* and *HHF2* does not restore damage bypass competence in a *rad53Δ* background (right), thus excluding histone dosage as a critical factor.

Data information: (A–E) Error bars indicate SD derived from three independent experiments.

© 2018 The Authors

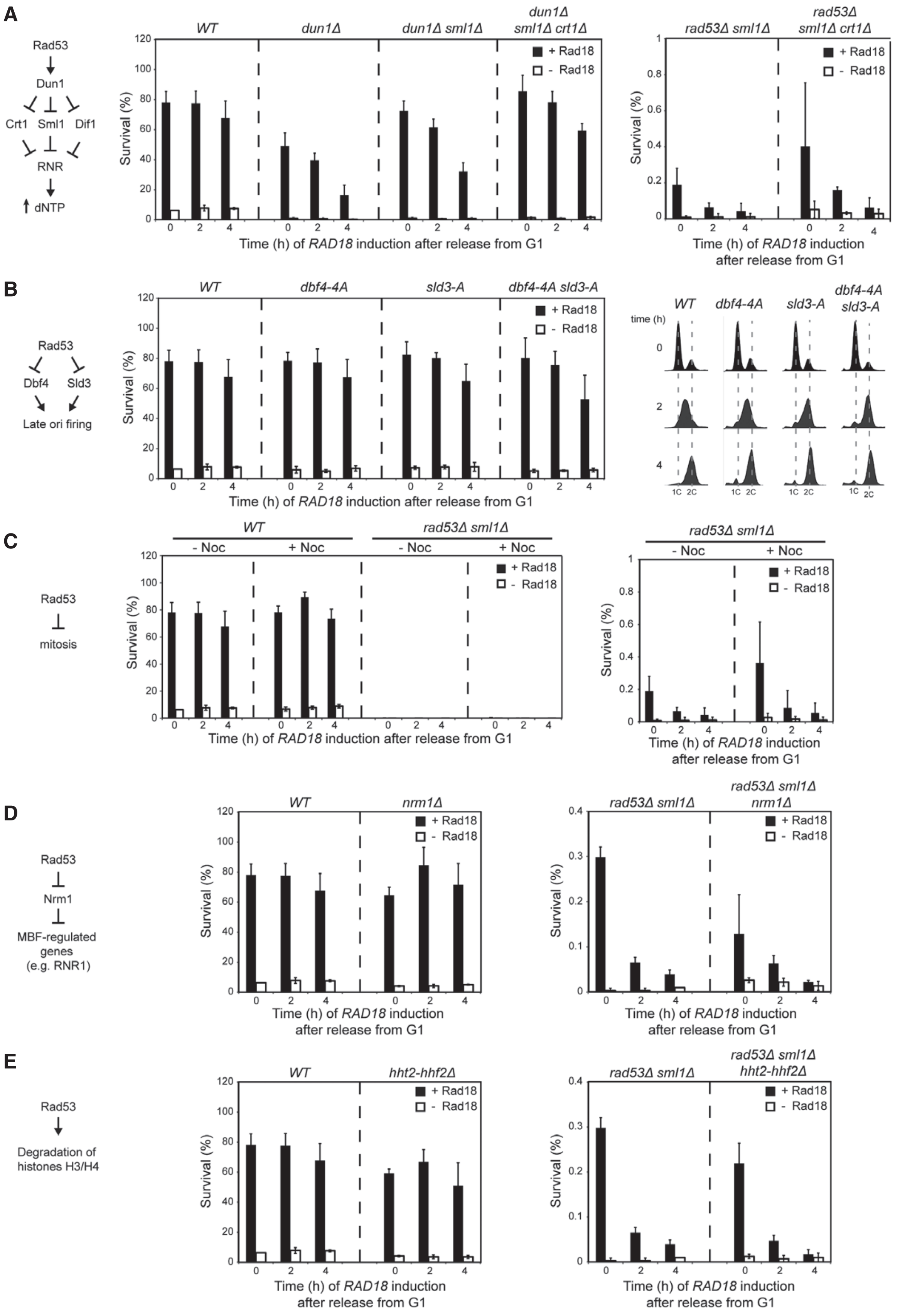

Figure EV3.

© 2018 The Authors

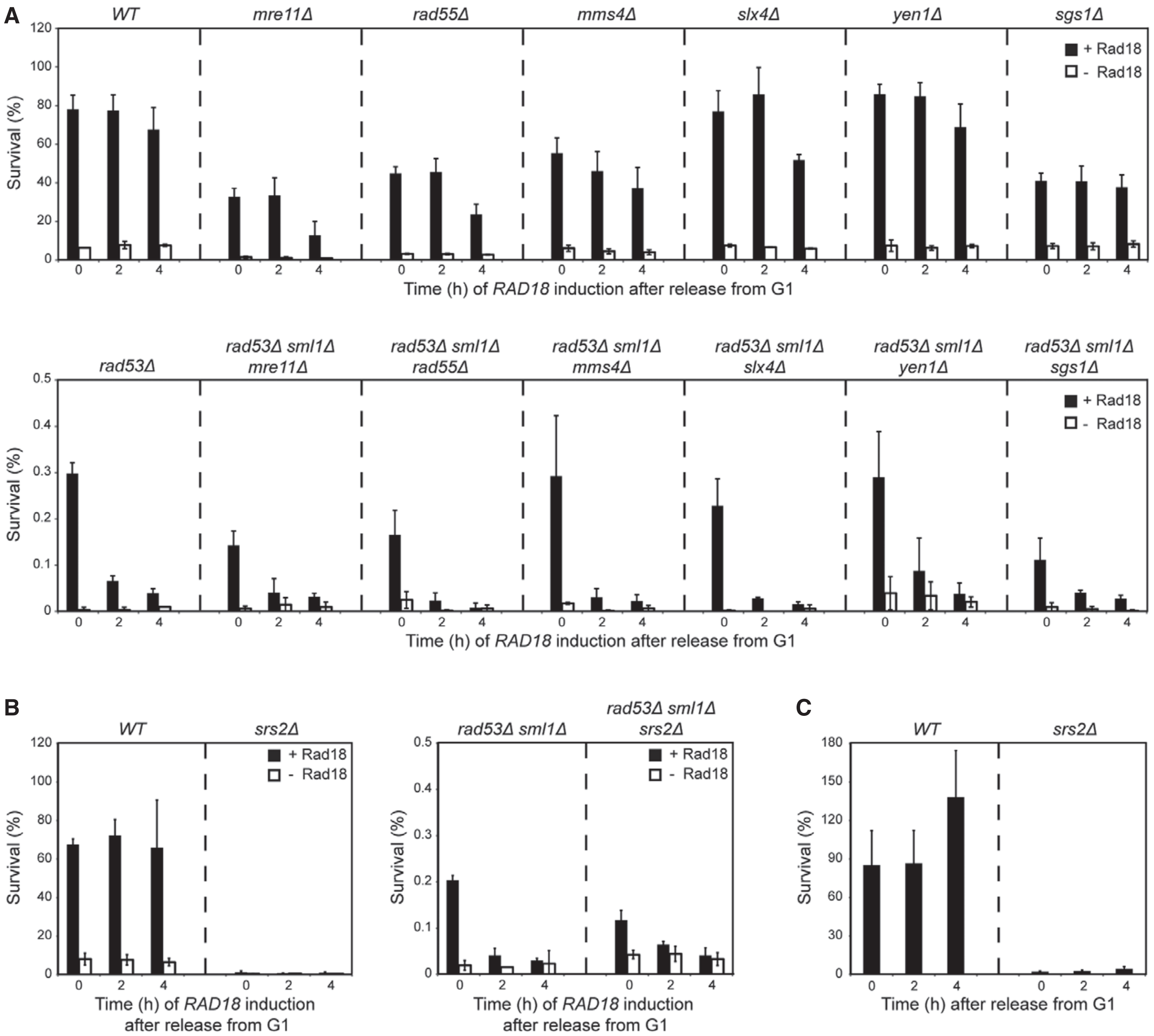

Figure EV4. Loss of DNA damage bypass competence in *rad53Δ* is not due to excessive homologous recombination.
Rad18-dependent recovery assays were performed according to the scheme in Fig 1B.

A Recovery of viability in strains defective in distinct stages of homologous recombination: *mre11Δ* (affecting DNA resection), *rad55Δ* (disabled in the formation of recombinogenic filaments), *mms4Δ*, *slx4Δ*, *yen1Δ*, and *sgs1Δ* (defective in the resolution or dissolution of joint molecules). Top: control assays performed in a *RAD53* background. Bottom: recovery assays of recombination mutants in combination with *rad53Δ*.

B Recovery assays of *srs2Δ* (left) and *srs2Δ rad53Δ* (right) indicate that elevated homologous recombination in the *srs2Δ* mutant (Aguilera & Klein, 1988) does not rescue the recovery defect of *rad53Δ*.

C Poor survival of UV-irradiated *srs2Δ* cells grown in the continuous presence of Rad18 indicates that the recovery defect in the *srs2Δ* background is independent of Rad18.

Data information: (A–C) Error bars indicate SD derived from three independent experiments.

© 2018 The Authors

Figure EV5. Exo1 and Pif1 are controlled by the DNA damage checkpoint and the cell cycle.

A As a control for the experiment shown in Fig 4A, recovery of viability upon *RAD18* induction was measured in the indicated mutants in a *RAD53* background.

B Exo1 and Rad53 phosphorylation is largely dependent on Rad9, with Mrc1 acting as a backup. Phosphorylation was assayed in the indicated strains upon release into S phase after UV irradiation (20 J/m^2) in the absence of Rad18. Exo1^{9myc} and Rad53 were detected by Western blotting. Pgk1 served as loading control.

C As a control for the experiment shown in Fig 4C, survival of UV-irradiated *exo1-SA* was measured in the continuous presence of Rad18.

D Exo1 phosphorylation is not completely abolished in *exo1-SA*. Exo1 phosphorylation was assayed in *WT* and *exo1-SA* cells released into S phase after UV irradiation in the absence of Rad18.

E Exo1 levels decline in a Rad53-independent manner in G2/M, but without significant dephosphorylation. Left: experimental scheme; right: time course of Rad53$^{AID*-9myc}$ and Exo1^{6HA} phosphorylation and protein levels in *rad53*$^{AID*-9myc}$ cells released into S phase after UV irradiation in the absence of Rad18. At 4 h after release, Rad53$^{AID*-9myc}$ degradation was induced by adding auxin to part of the culture. Pgk1 was used as loading control. Cell cycle profiles are shown at the bottom.

F Time course of Exo1^{9myc} levels in *WT* cells released into S phase in the absence of damage or after UV irradiation. Re-entry into the next S phase was prevented by adding αF after 60 or 90 min, respectively. Exo1^{9myc} protein levels, relative to G1 (0 min) and normalized to the Pgk1 signal, are plotted on the right.

G Time course of Pif1^{6HA} levels in *WT* cells performed as in panel (F). Pif1^{6HA} protein levels, relative to G1 (0 min) and normalized to the Pgk1 signal, are plotted on the right.

Data information: (A, C) Error bars indicate SD derived from three independent experiments.

Source data are available online for this figure.

 © 2018 The Authors

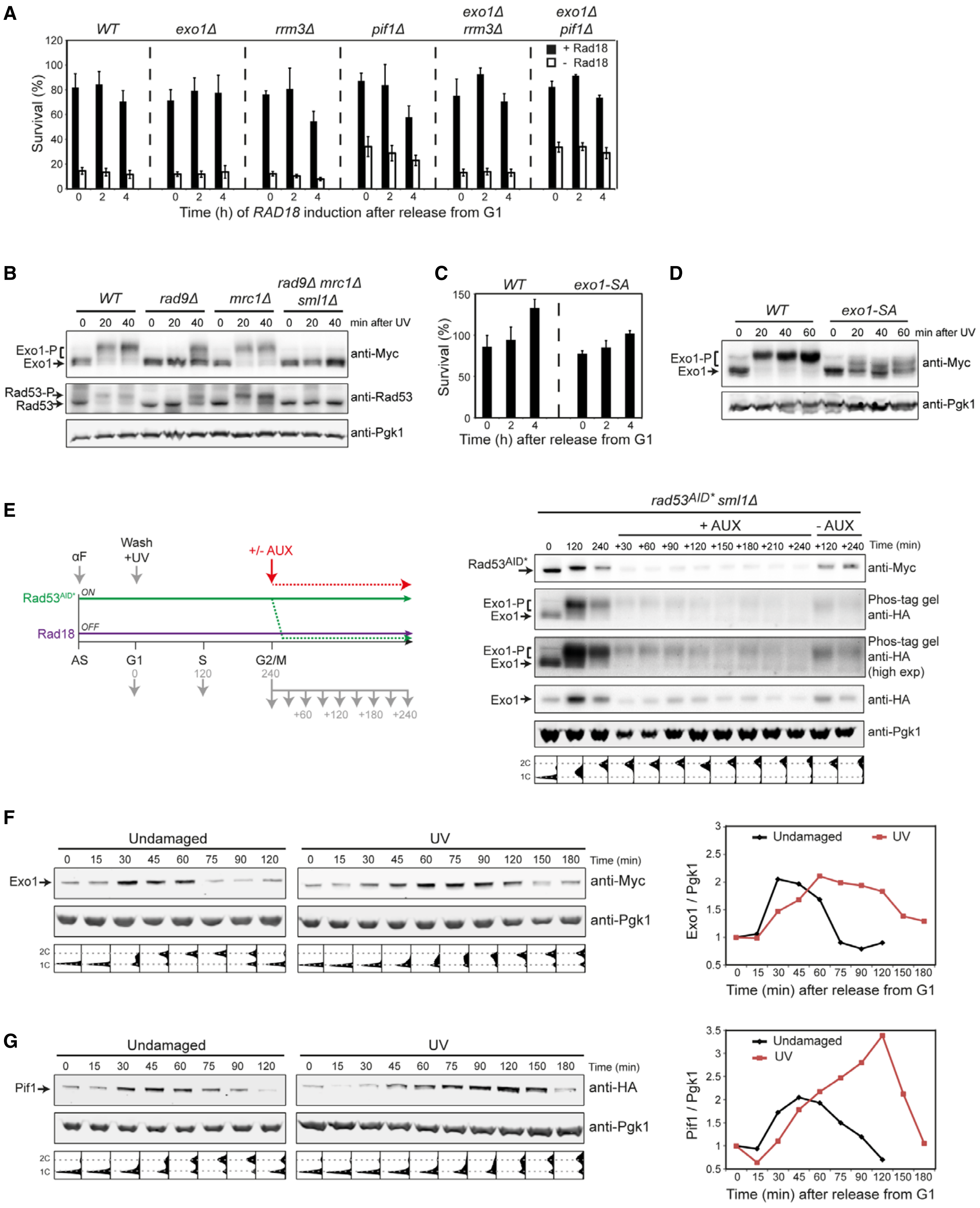
A
WT
exo1Δ
rrm3Δ
pif1Δ
exo1Δ rrm3Δ
exo1Δ pif1Δ
+ Rad18
- Rad18
Survival (%)
Time (h) of RAD18 induction after release from G1
B
WT
rad9Δ
mrc1Δ
rad9Δ mrc1Δ sml1Δ
min after UV
Exo1-P
Exo1
Rad53-P
Rad53
anti-Myc
anti-Rad53
anti-Pgk1
C
WT
exo1-SA
Survival (%)
Time (h) after release from G1
D
WT
exo1-SA
min after UV
Exo1-P
Exo1
anti-Myc
anti-Pgk1
E
αF
Wash +UV
+/- AUX
Rad53AID*
ON
Rad18
OFF
AS
G1
S
G2/M
rad53AID* sml1Δ
+ AUX
- AUX
Time (min)
Rad53AID*
anti-Myc
Exo1-P
Exo1
Phos-tag gel anti-HA
Phos-tag gel anti-HA (high exp)
anti-HA
anti-Pgk1
2C
1C
F
Undamaged
UV
Time (min)
Exo1
anti-Myc
anti-Pgk1
Exo1 / Pgk1
Time (min) after release from G1
G
Undamaged
UV
Time (min)
Pif1
anti-HA
anti-Pgk1
Pif1 / Pgk1
Time (min) after release from G1

Figure EV5.
