# Supplementary figures and images for: Spatial separation between replisome‐ and template‐induced replication stress signaling
Source: EMBO J. 2018 Mar 26;37(9):e98369. doi: 10.15252/embj.201798369 (PMC5920239; doi:10.15252/embj.201798369)

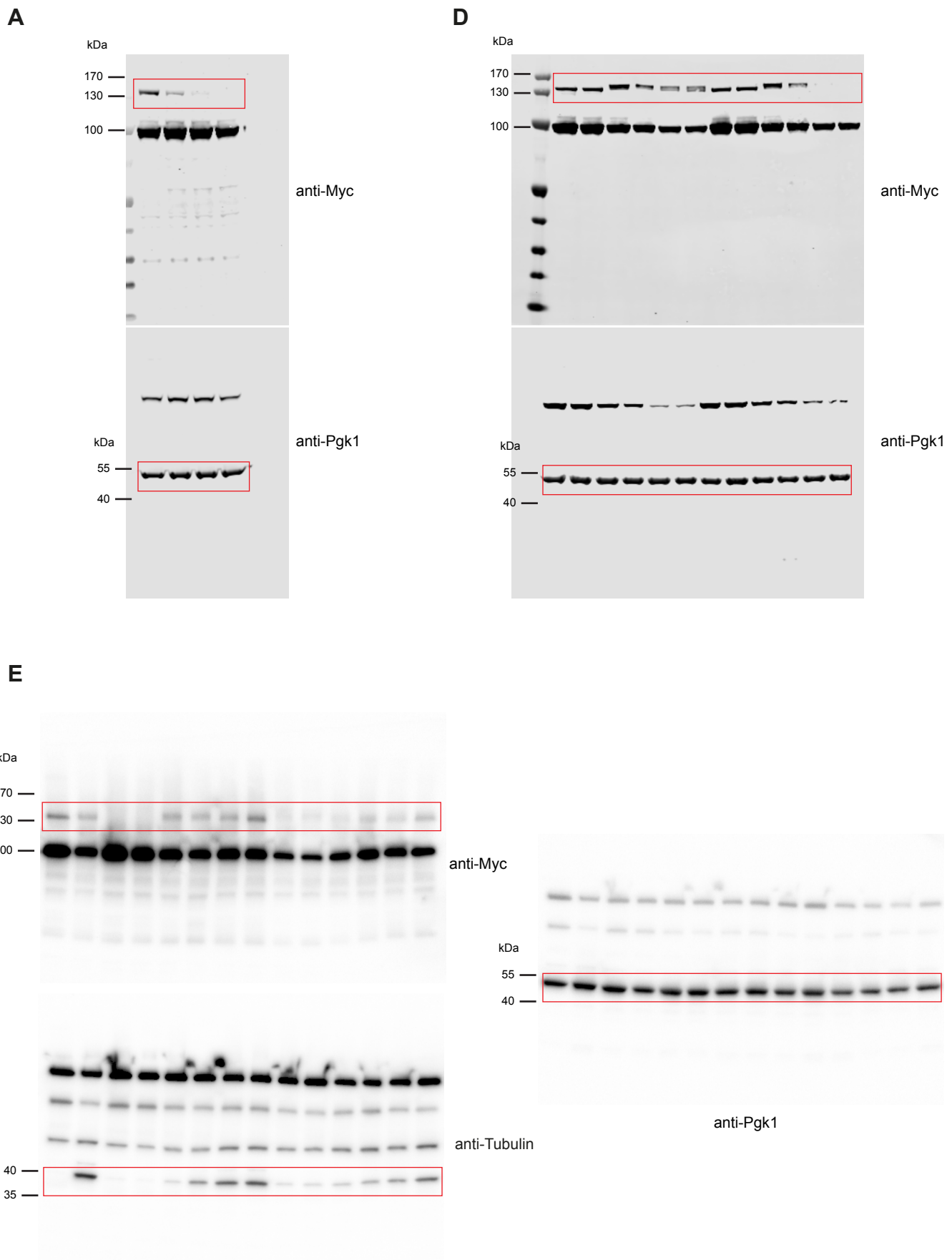

Supplement: Supplementary file 3 — Source Data for Expanded View [file EMBJ-37-e98369-s005.zip › EMBOJ_98369_source_data_for_Appendix_Figures/EMBOJ_98369_source_data_for_Appendix_Fig_S1.pdf]

**A**

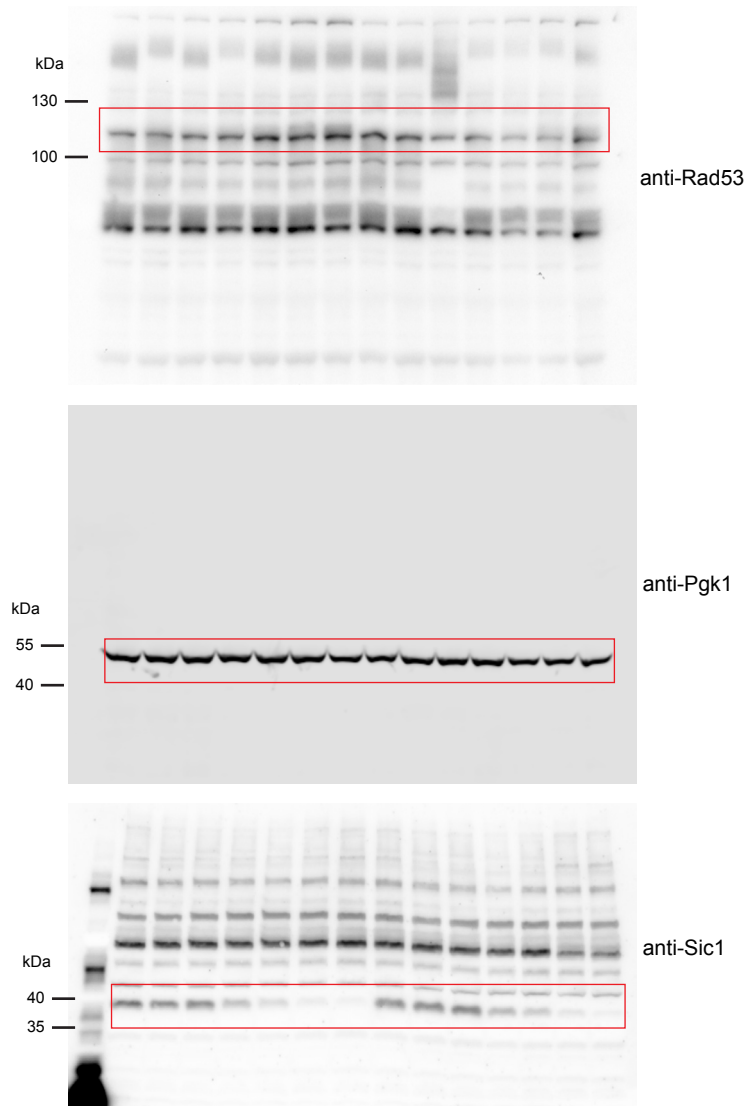

Supplement: Supplementary file 3 — Source Data for Expanded View [file EMBJ-37-e98369-s005.zip › EMBOJ_98369_source_data_for_Appendix_Figures/EMBOJ_98369_source_data_for_Appendix_Fig_S2.pdf]

**B**

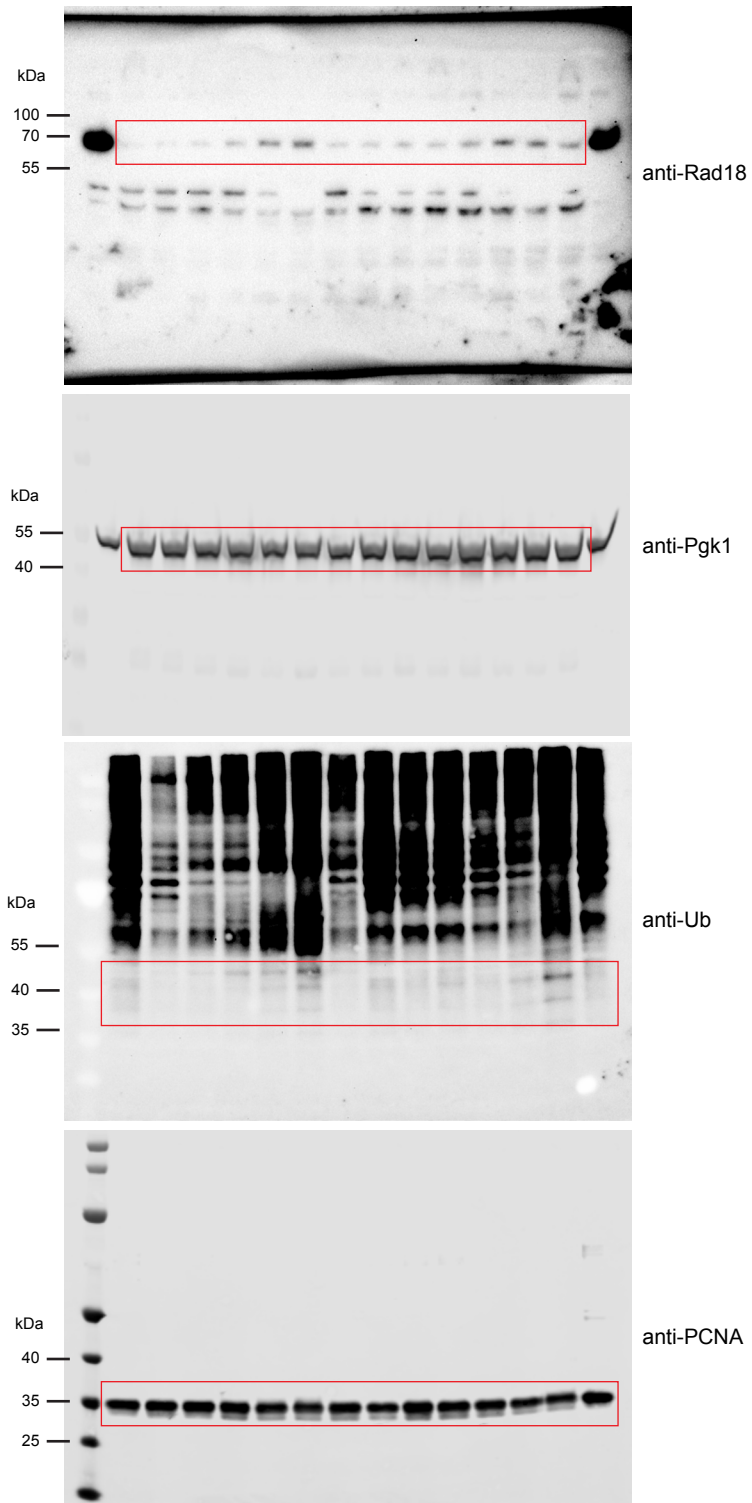

**G**

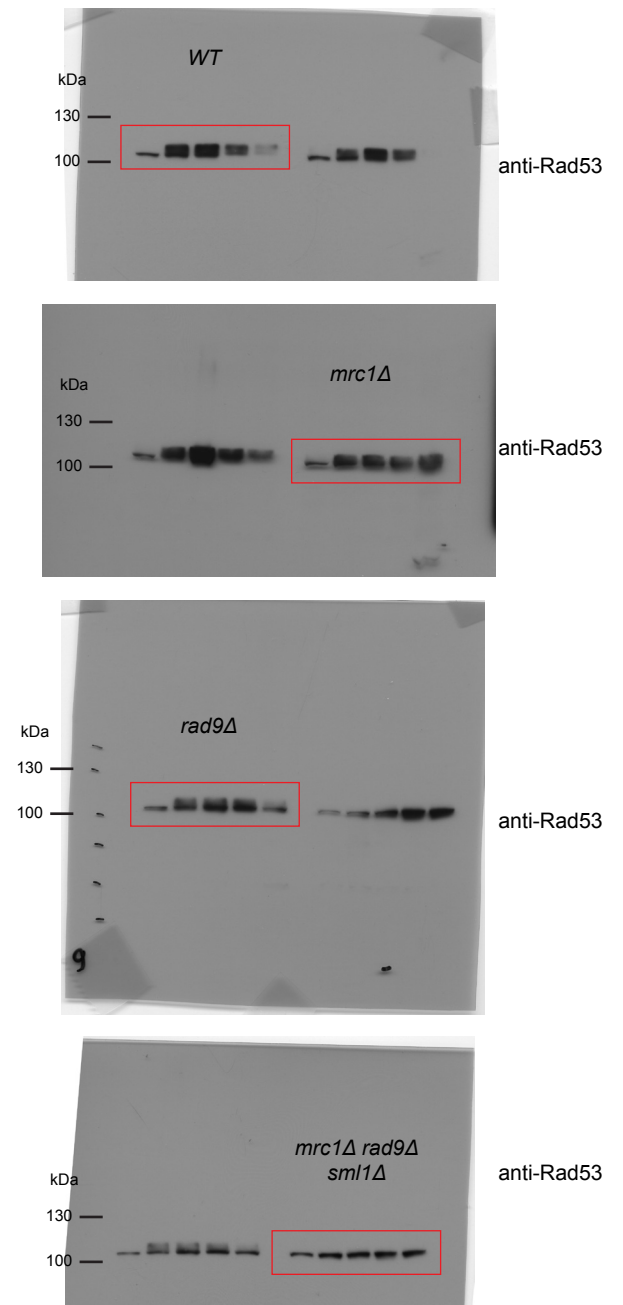

Supplement: Supplementary file 3 — Source Data for Expanded View [file EMBJ-37-e98369-s005.zip › EMBOJ_98369_source_data_for_EV_figures/EMBOJ_98369_source_data_for_Fig_EV1.pdf]

**B**

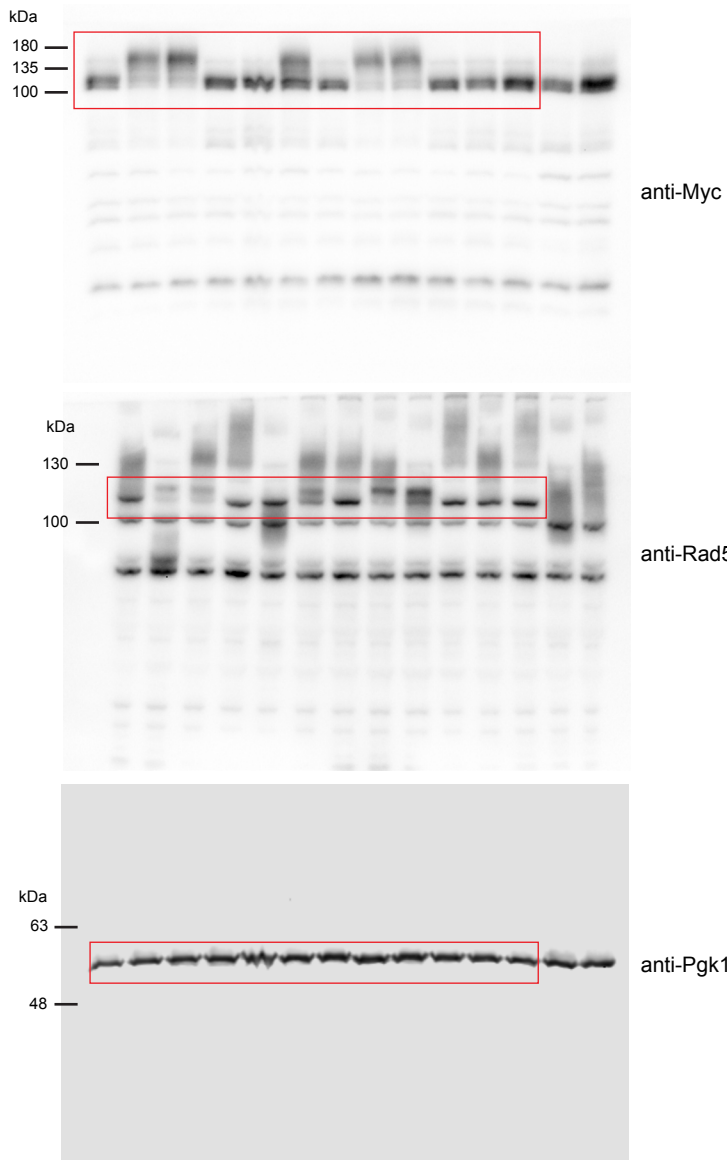

**D**

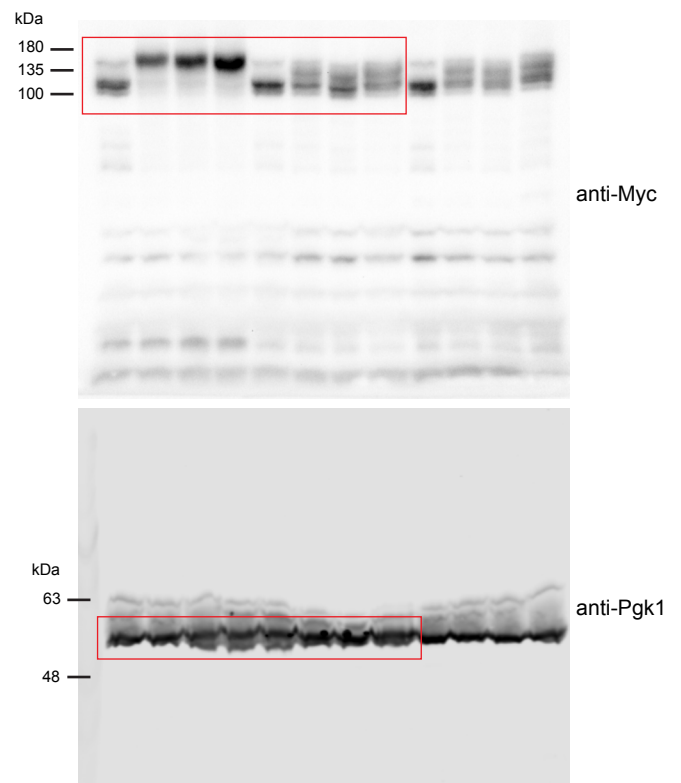

**E**

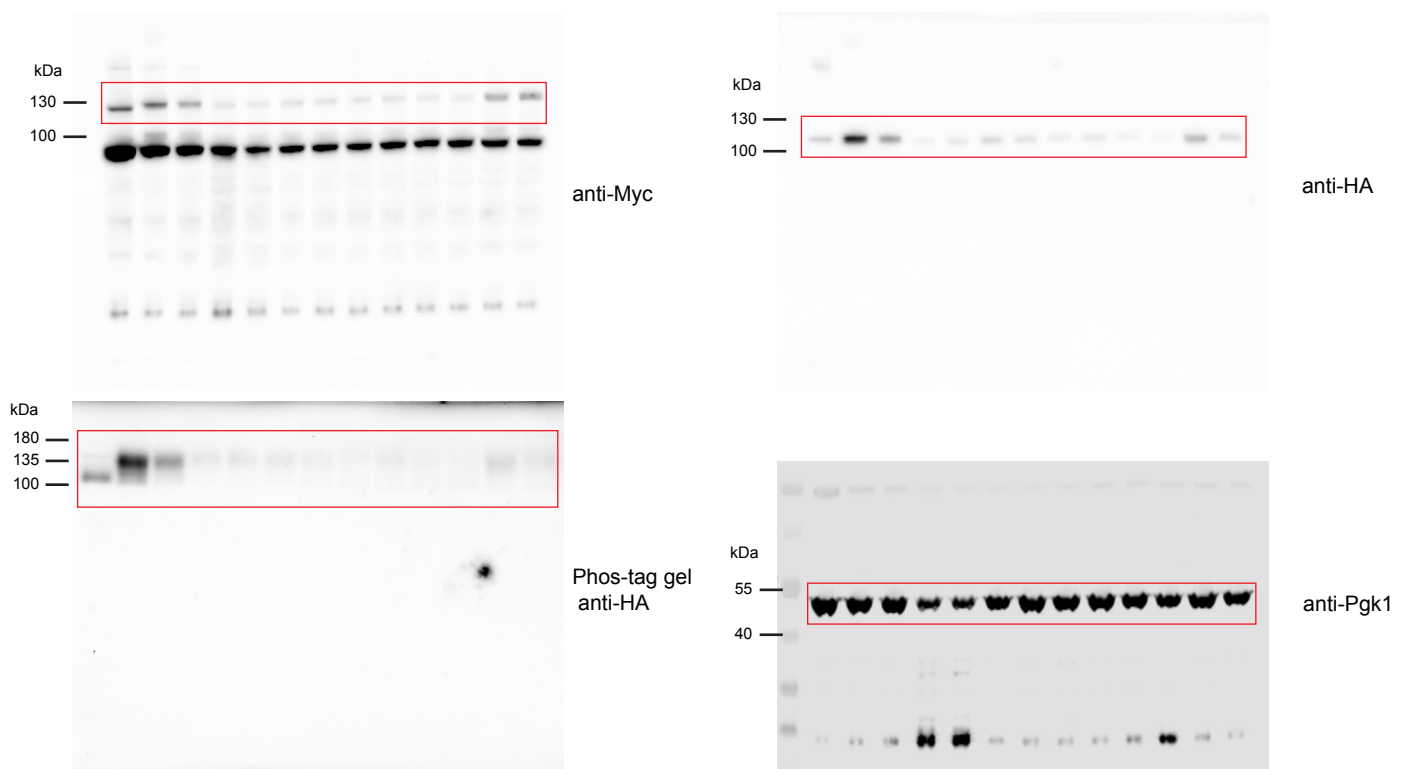

**F**

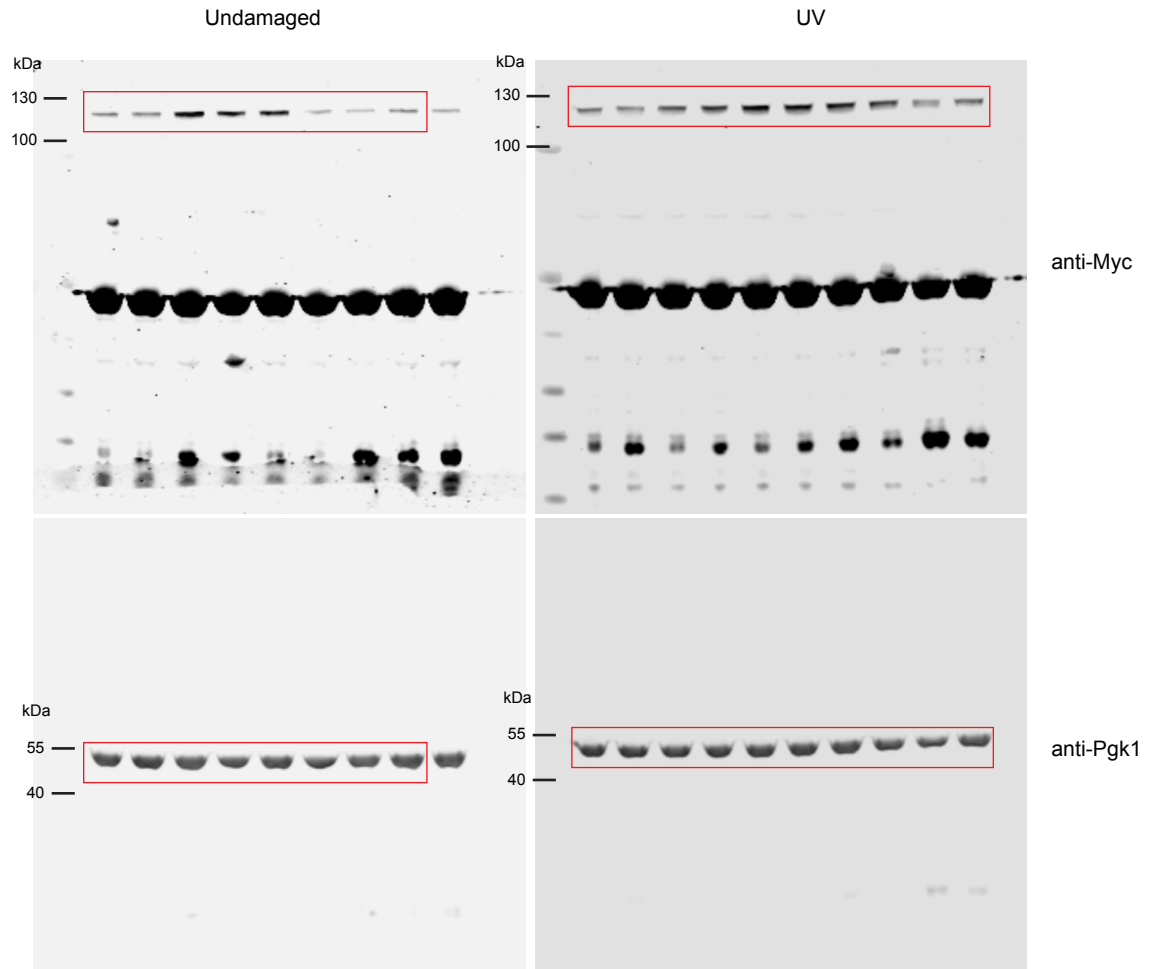

**G**

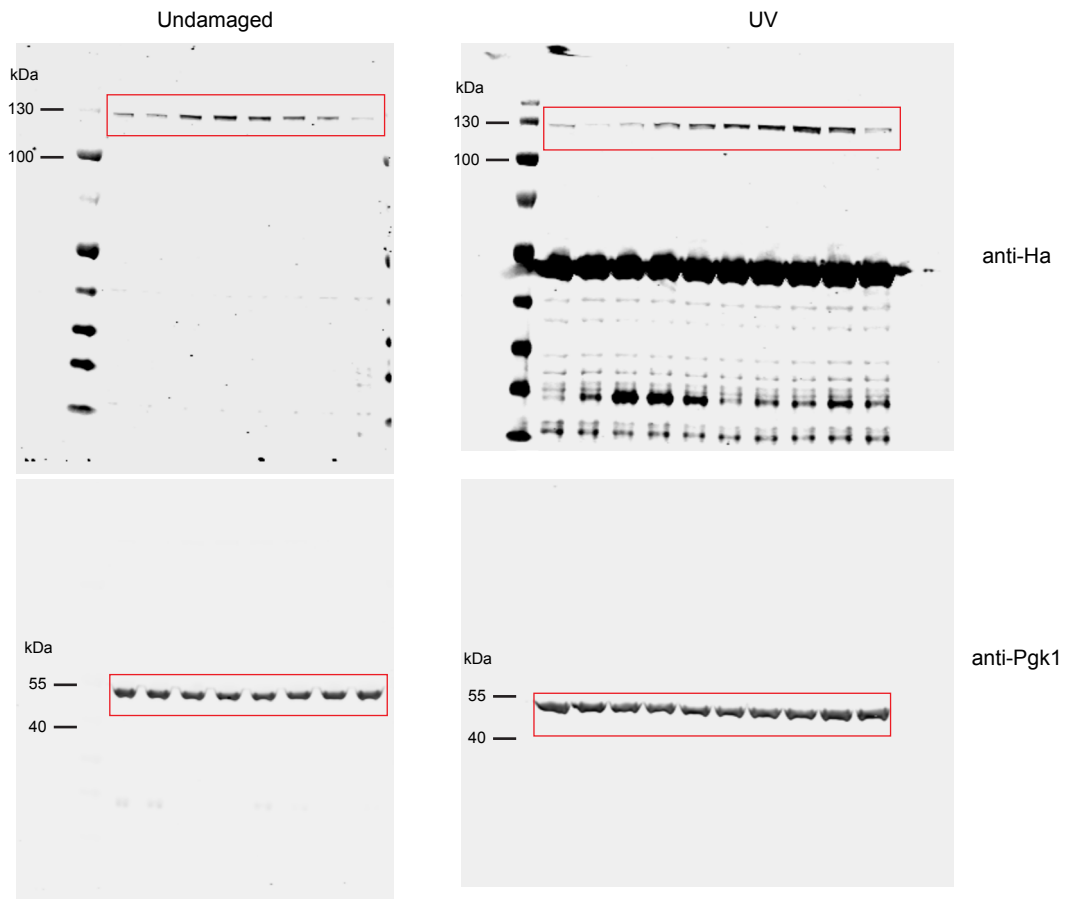

Supplement: Supplementary file 3 — Source Data for Expanded View [file EMBJ-37-e98369-s005.zip › EMBOJ_98369_source_data_for_EV_figures/EMBOJ_98369_source_data_for_Fig_EV5.pdf]

**B**

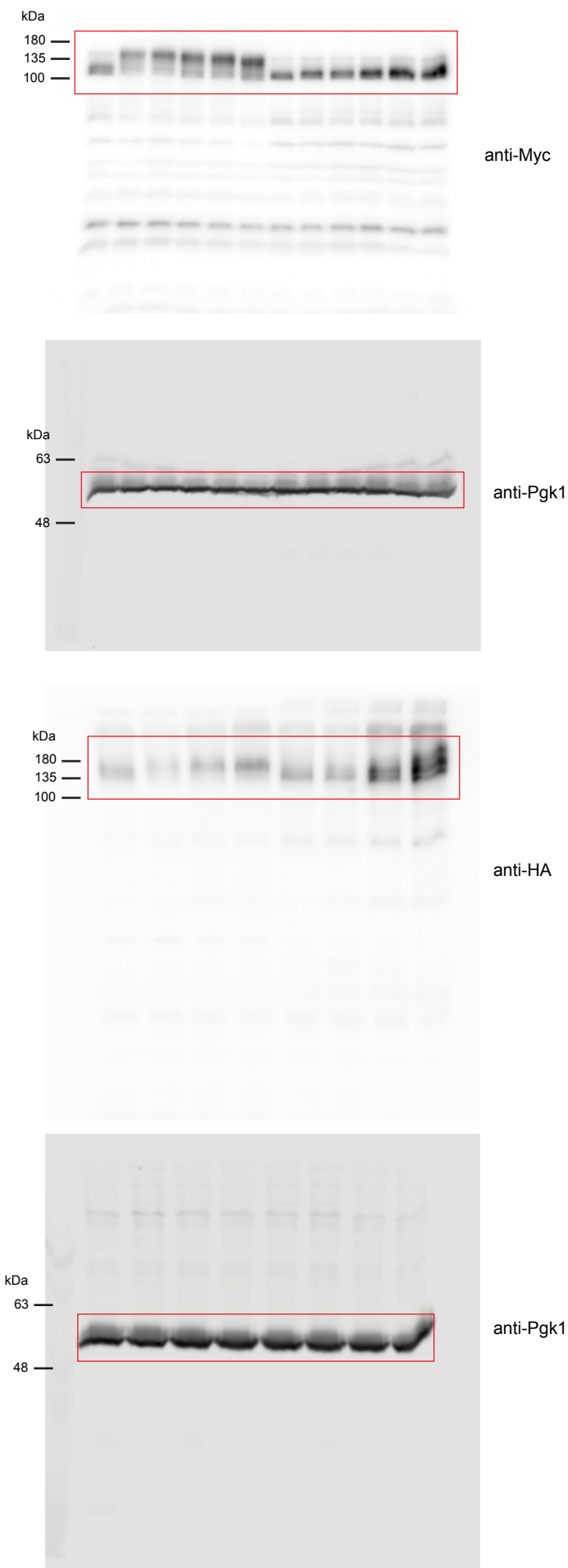

Supplement: Supplementary file 5 — Source Data for Figure 4 [file EMBJ-37-e98369-s003.pdf]

**A**

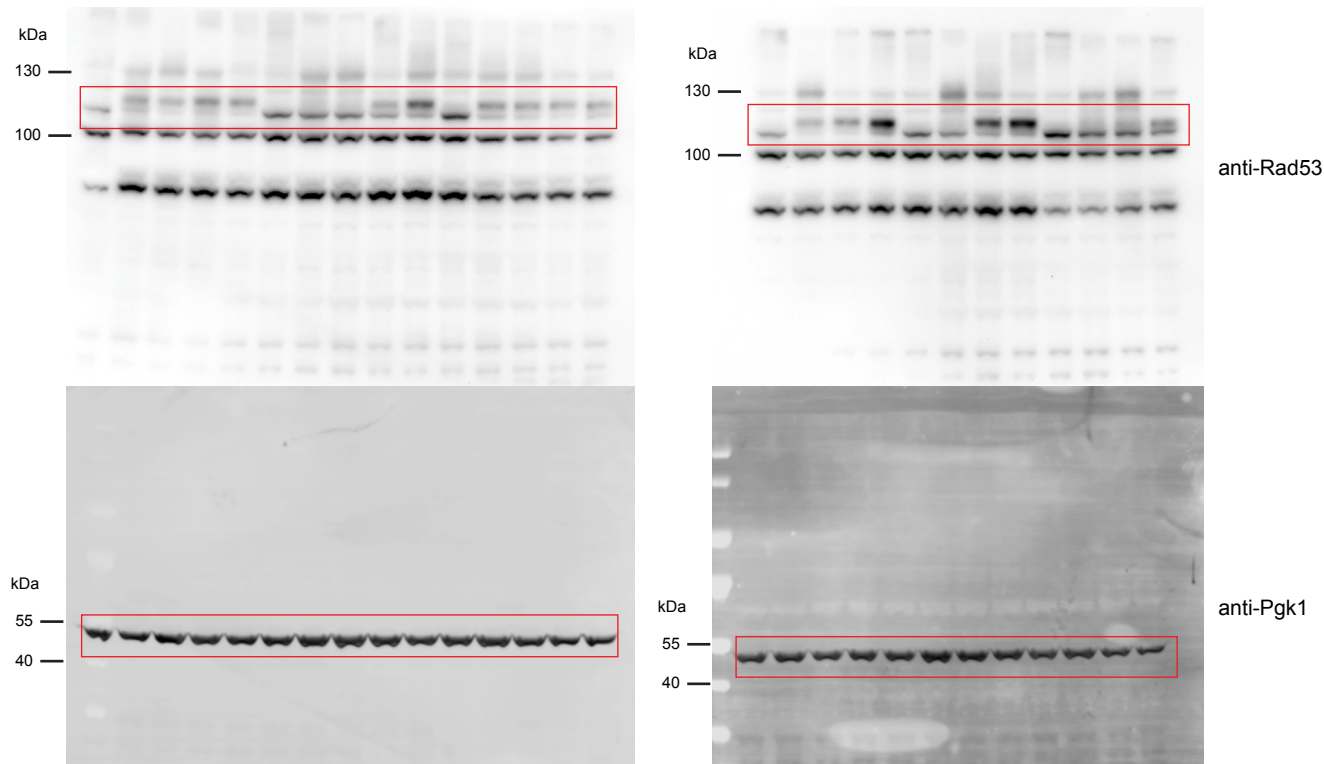

**B**

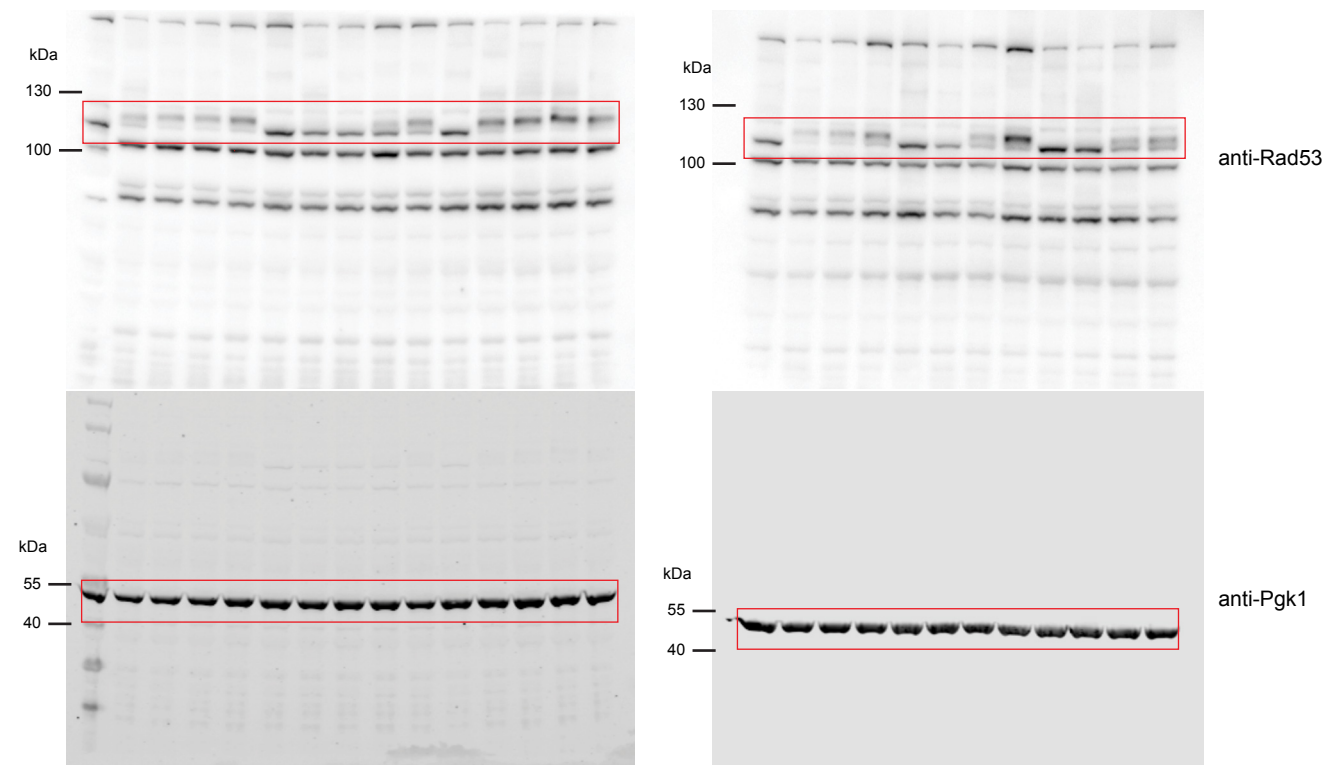

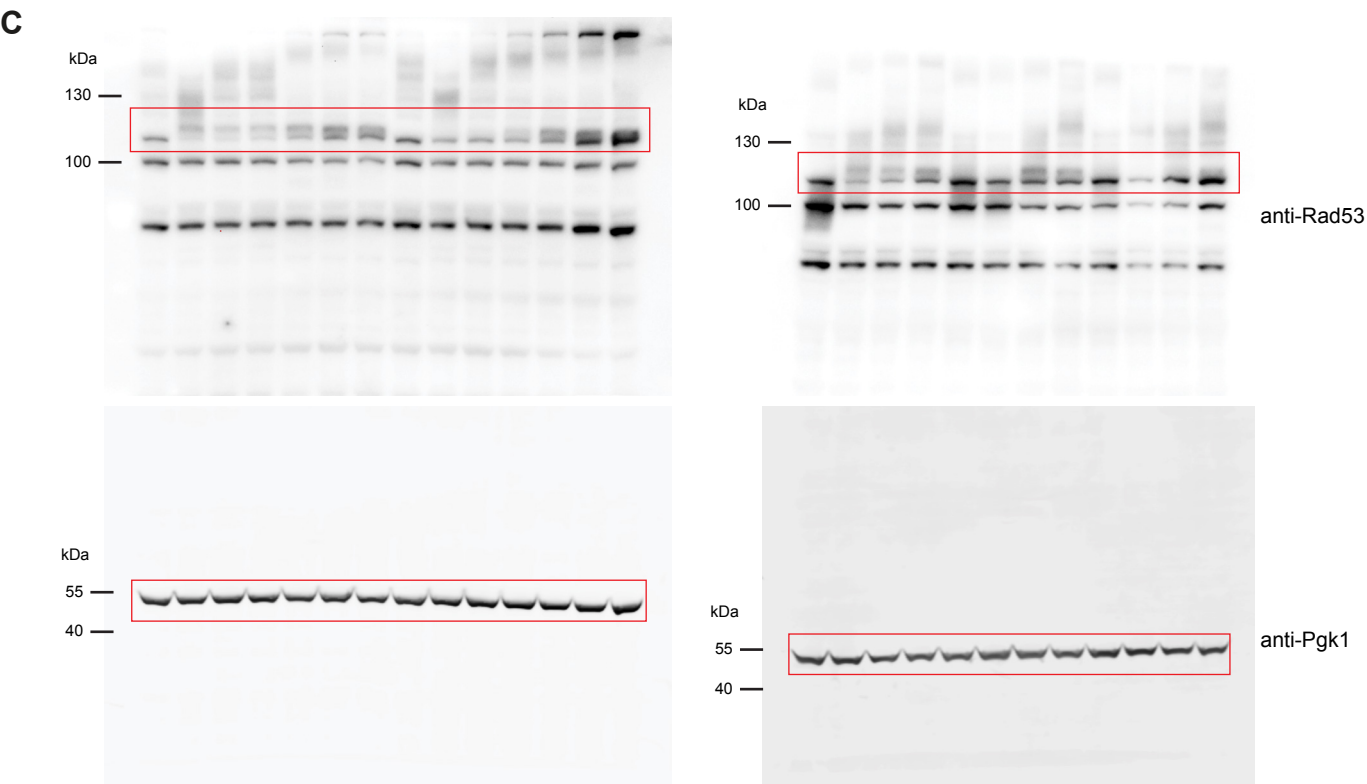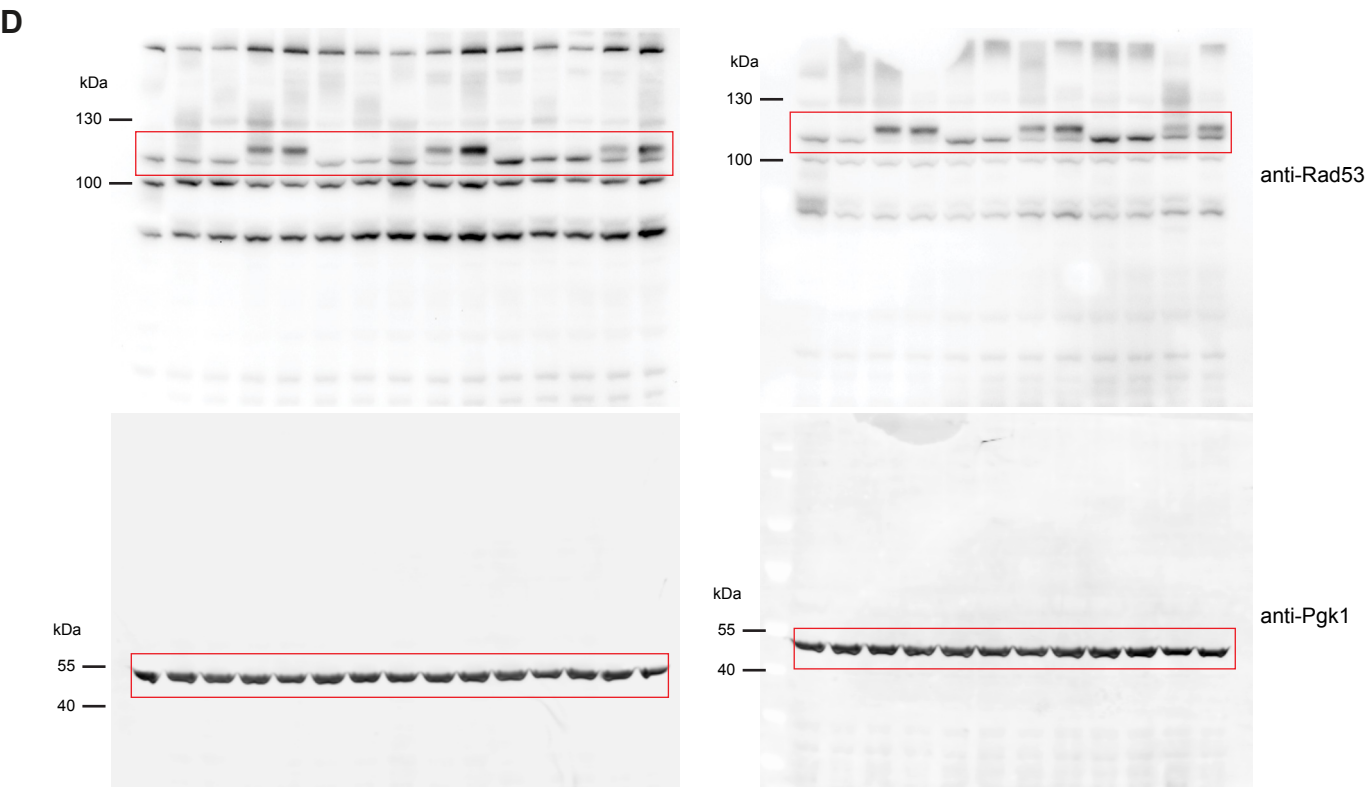

**E**

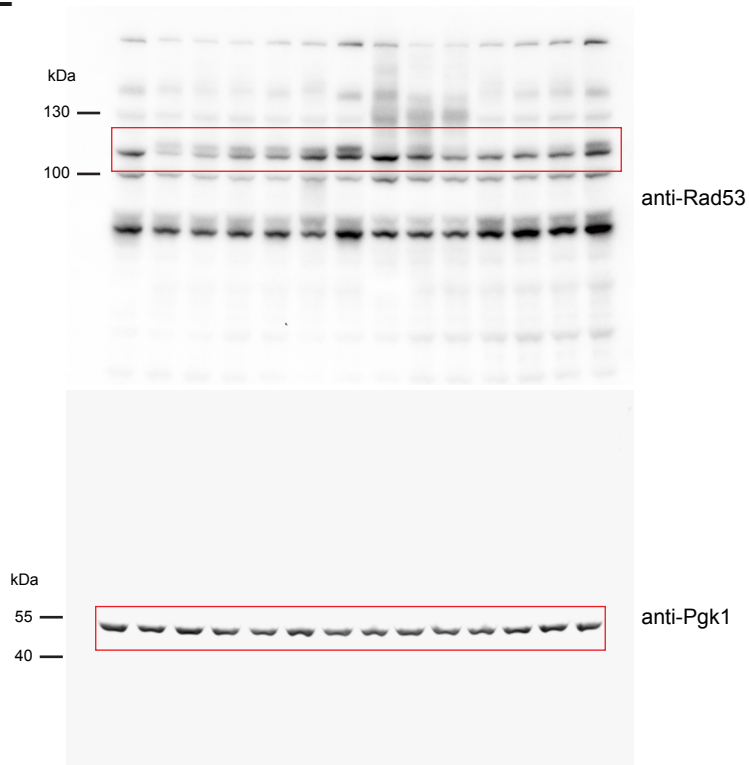

**G**

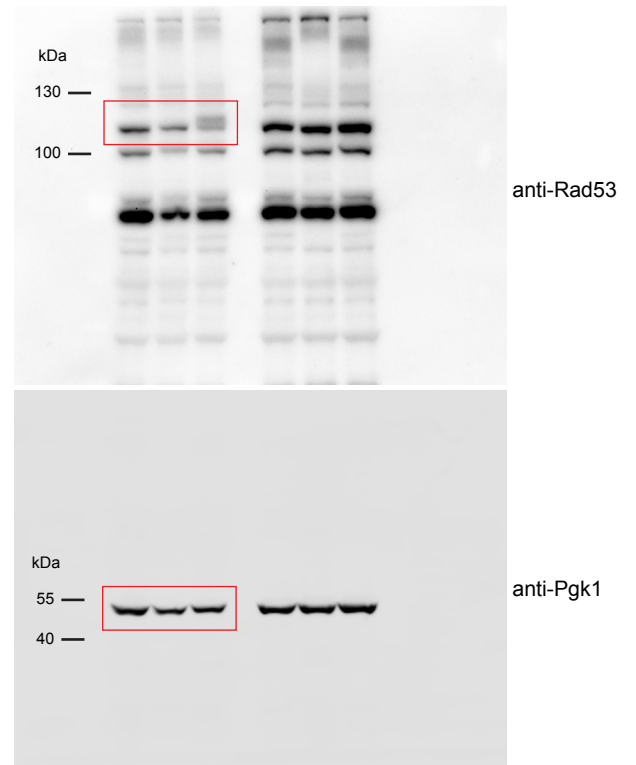

**F**

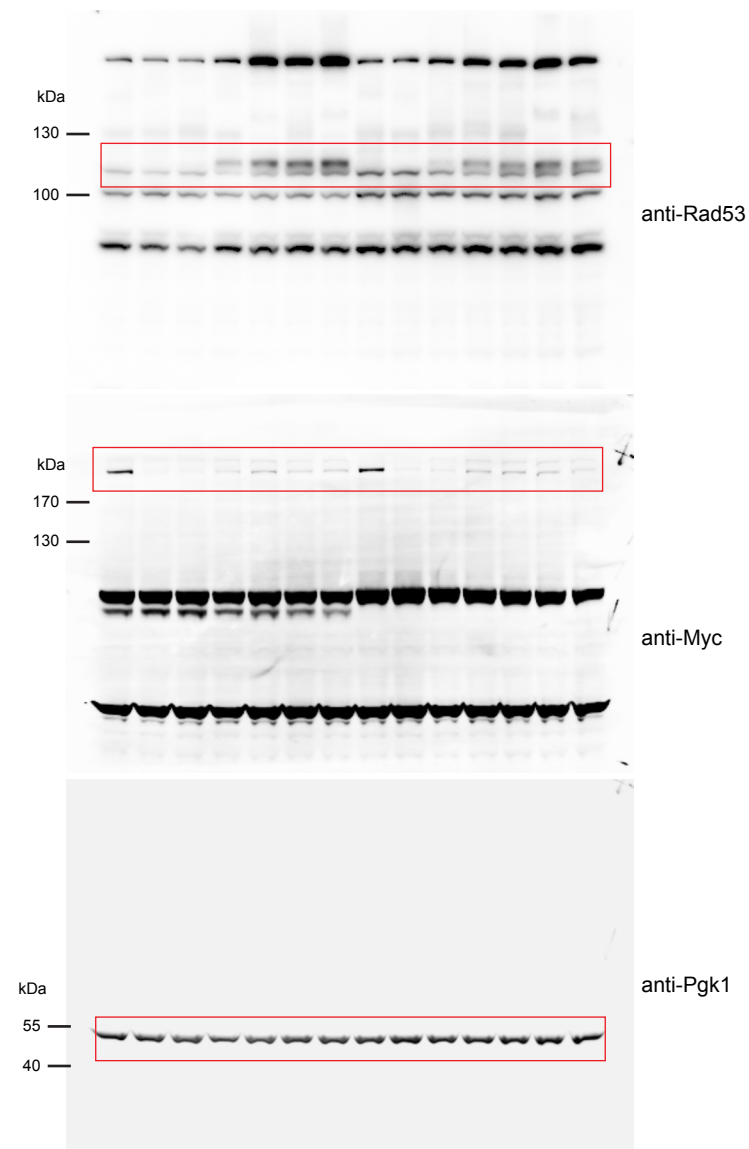

**H**

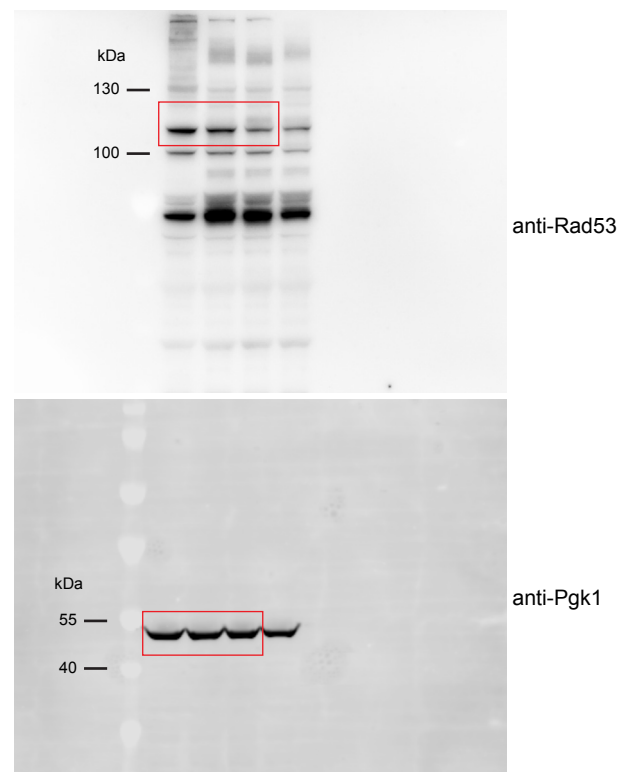

Supplement: Supplementary file 6 — Source Data for Figure 5 [file EMBJ-37-e98369-s004.pdf]
